# Phylogeny-Aware Chemoinformatic Analysis of Chemical Diversity in Lamiaceae Enables Iridoid Pathway Assembly and Discovery of Aucubin Synthase

**DOI:** 10.1093/molbev/msac057

**Published:** 2022-03-17

**Authors:** Carlos E. Rodríguez-López, Yindi Jiang, Mohamed O. Kamileen, Benjamin R. Lichman, Benke Hong, Brieanne Vaillancourt, C. Robin Buell, Sarah E. O'Connor

**Affiliations:** 1 Department of Natural Product Biosynthesis, Max Planck Institute for Chemical Ecology, 07745 Jena, Germany; 2 Department of Biology, University of York, YO10 5DD York, UK; 3 Center for Applied Genetic Technologies, University of Georgia, Athens, GA 30602, USA; 4 Department of Crop & Soil Sciences, University of Georgia, Athens, GA 30602, USA

**Keywords:** iridoids, chemical diversity, cytochrome P450, pathway reconstruction, cheminformatics, comparative biochemistry

## Abstract

Countless reports describe the isolation and structural characterization of natural products, yet this information remains disconnected and underutilized. Using a cheminformatics approach, we leverage the reported observations of iridoid glucosides with the known phylogeny of a large iridoid producing plant family (Lamiaceae) to generate a set of biosynthetic pathways that best explain the extant iridoid chemical diversity. We developed a pathway reconstruction algorithm that connects iridoid reports via reactions and prunes this solution space by considering phylogenetic relationships between genera. We formulate a model that emulates the evolution of iridoid glucosides to create a synthetic data set, used to select the parameters that would best reconstruct the pathways, and apply them to the iridoid data set to generate pathway hypotheses. These computationally generated pathways were then used as the basis by which to select and screen biosynthetic enzyme candidates. Our model was successfully applied to discover a cytochrome P450 enzyme from *Callicarpa americana* that catalyzes the oxidation of bartsioside to aucubin, predicted by our model despite neither molecule having been observed in the genus. We also demonstrate aucubin synthase activity in orthologues of *Vitex agnus-castus*, and the outgroup *Paulownia tomentosa*, further strengthening the hypothesis, enabled by our model, that the reaction was present in the ancestral biosynthetic pathway. This is the first systematic hypothesis on the *epi*-iridoid glucosides biosynthesis in 25 years and sets the stage for streamlined work on the iridoid pathway. This work highlights how curation and computational analysis of widely available structural data can facilitate hypothesis-based gene discovery.

## Introduction

**Fig. 1.**
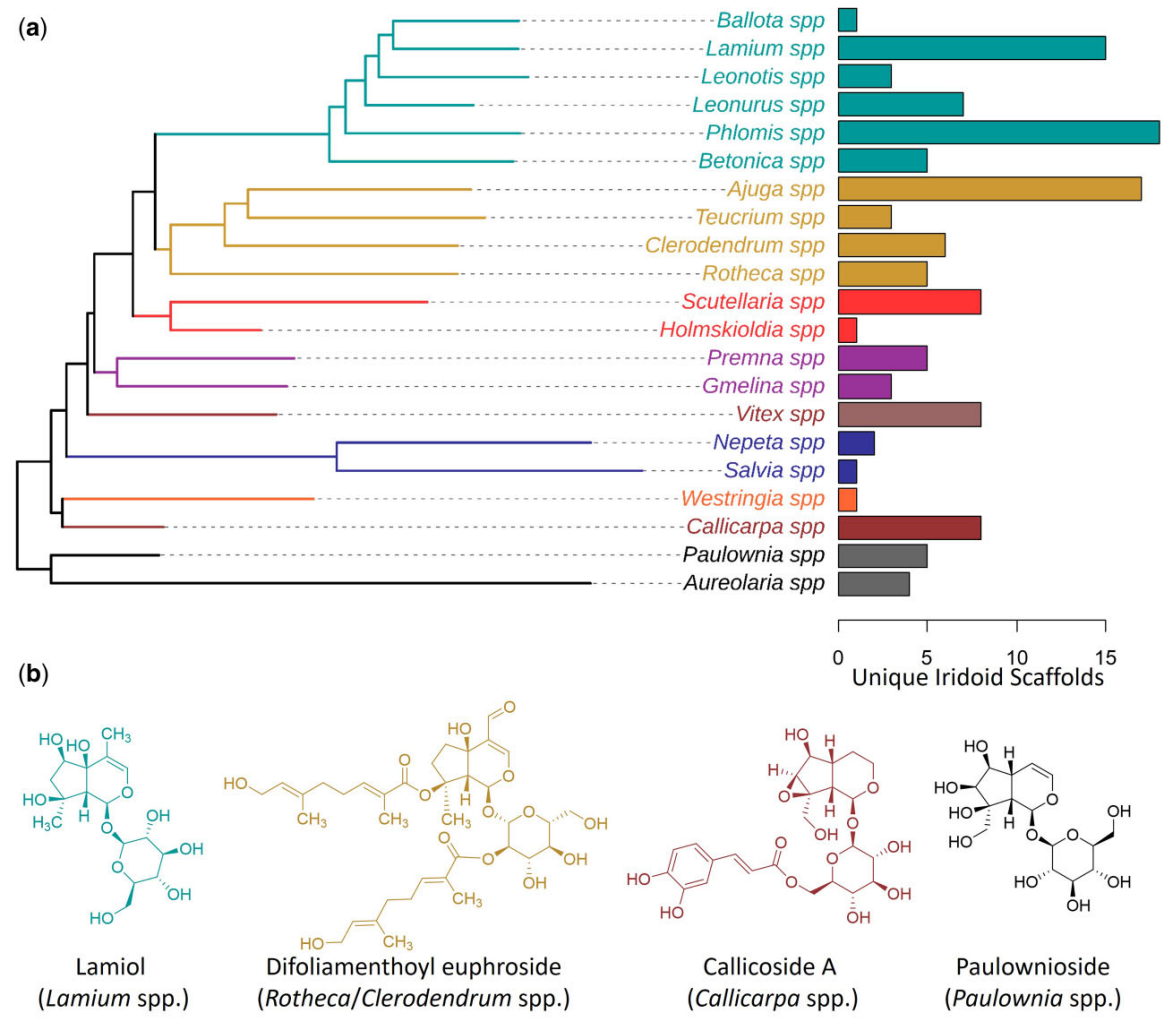
Reported chemical diversity of the Lamiaceae. (*a*) Maximum likelihood tree of 19 selected genera from Lamiaceae and two Lamiales outgroups, as inferred by the Mint Evolutionary Genomics Consortium (2018), pruned from genera with no reports of iridoid glucosides, along with unique iridoid scaffold reports (after removing decorations) for each genus. Branches, labels, and bars are color-coded by clade, from top to bottom: Lamioideae in Persian green, Ajugoideae in tussock yellow, Scutellaroideae in pomegranate red, Premnoideae in plum violet, Viticoideae in copper rose, Nepetoideae in sapphire blue, Prostantheroideae in vermilion, Callicarpoideae in crail red, and the outgroups (Paulowniaceae and Orobanchaceae) in dark gray. (*b*) Example of chemotaxonomically important iridoid glucosides in four selected clades.

In the last 100 years, there has been a myriad of reports describing the isolation and structural characterization of natural products from plants. This has yielded a plethora of information on chemical diversity in the literature that is dispersed and underutilized. This is exemplified by iridoid glucosides, plant-derived natural products classified as noncanonical monoterpenes. Iridoid glucosides are found throughout the Asterids (Stull et al. 2018) and are characterized by a distinctive cyclopentanopyran (nepetalactol) fused ring system (fig. 1). Iridoid chemical diversity is derived from oxidation and decoration of this iridoid core scaffold, with the variation likely serving as a means of counteracting herbivore detoxification mechanisms (Dobler et al. 2011).

Iridoids’ vast chemical diversity is of chemotaxonomic interest, and their numerous reports have been periodically aggregated and reviewed for decades (El-Naggar and Beal 1980; Boros and Stermitz 1990, 1991; Dinda et al. 2007a, 2007b, 2009, 2011; Wang et al. 2020). However, the biosynthetic pathways that generate these highly decorated iridoid glucosides—like many plant-derived natural products—remain largely unknown. Elucidating the genetic components of plant natural product pathways is a difficult and time-consuming process, requiring an accurate biochemical hypothesis to base functional characterization assays of biosynthetic gene candidates. Although essential for discovering the genetic components of biosynthetic pathways, an accurate hypothesis for biochemical transformations is often one of the most challenging aspects of the gene discovery process, relying on chemical intuition, isolation of biosynthetic intermediates, and labeling experiments.

Here, we use a large set of chemical data accumulated for the iridoid glucosides, along with the known phylogeny of a large iridoid producing plant family (Lamiaceae), to computationally generate an accurate set of biosynthetic pathways that best explain the currently observed chemical diversity of iridoids in Lamiaceae. These pathways were used to then select and screen biosynthetic enzyme candidates. We demonstrate the application of our computational model through the discovery and functional characterization of a *Callicarpa americana* CYP72 and orthologues in *Vitex agnus-castus* and *Paulownia tomentosa* in which we demonstrate biosynthesis of the iridoid glucoside aucubin using bartsioside as a substrate. Notably, neither bartsioside nor aucubin had been previously reported in *Callicarpa* spp., yet both molecules, as well as the reaction connecting them, are predicted by our model for the genus iridoid pathway.

## Results and Discussion

### Section 1: Pathway Reconstruction Algorithms

As a first step toward reconstructing the network of iridoid glucoside biosynthetic pathways in Lamiaceae, we compiled literature reports, consisting of 274 structurally distinct iridoids from 19 Lamiaceae genera and 2 outgroups from the Lamiales (*Paulownia* and *Aureolaria*; supplementary file 1, Supplementary Material online); for simplicity, observations were grouped at the genus level (fig. 1*a*). Iridoid glucosides consist of a core nepetalactol scaffold, modified by oxidation and decorated with methyl groups, sugars, phenolics, or terpenes, among other decorations (fig. 1*b*). The undecorated oxidized scaffold is the basis of iridoid glucosides diversity in Lamiaceae; thus, we removed the decorations from the structures prior to analysis. In total, 64 unique oxidized iridoid scaffolds are identified in Lamiaceae, distributed in 131 unique scaffold-species pairs, as shown in fig. 1*a*, which also shows representative iridoid glucosides from selected species (fig. 1*b*).

**Fig. 2.**
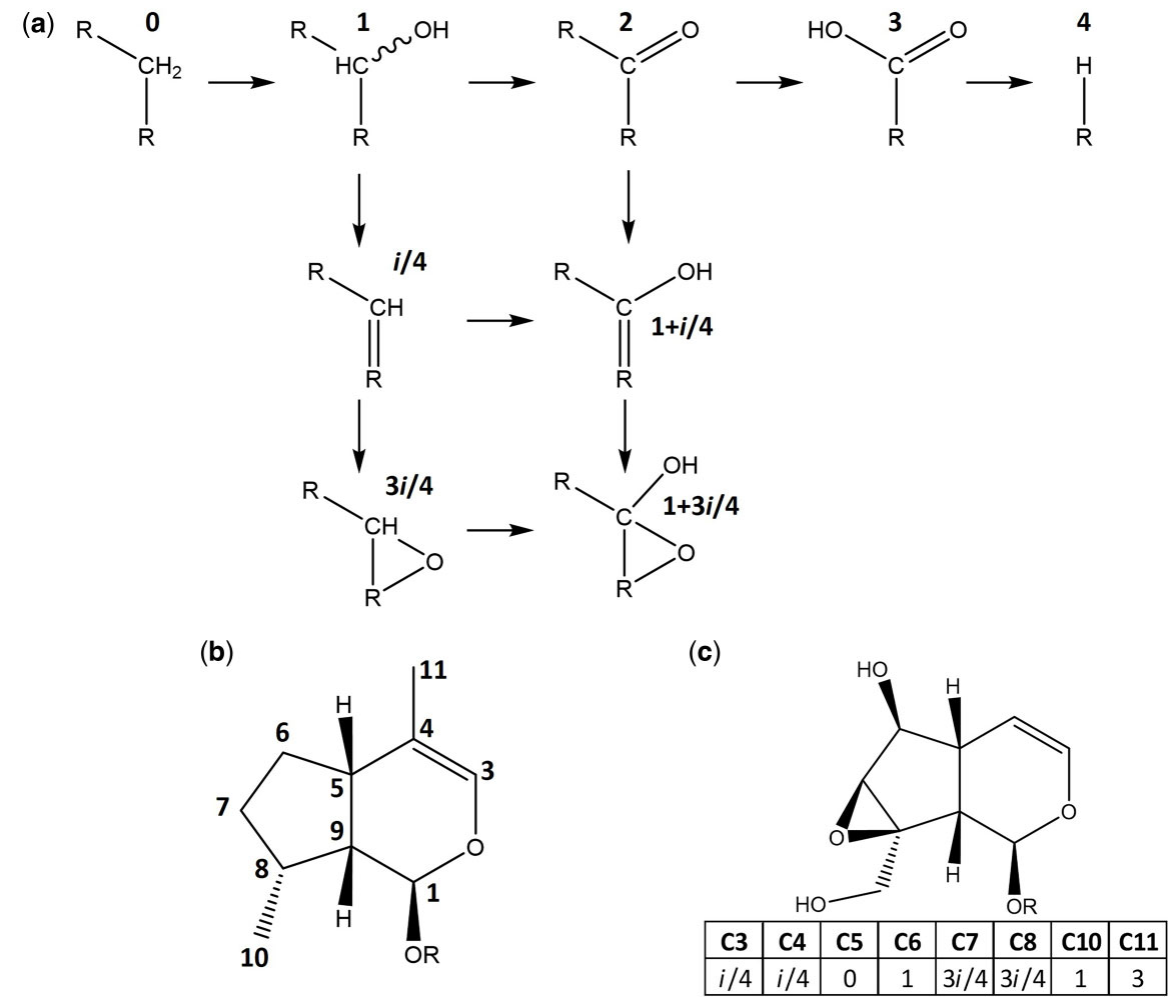
Mathematical representation of iridoid glucosides. (*a*) Individual carbons are assigned a value according to their oxidation state. (*b*) Each carbon has a number assigned, and thus, each iridoid scaffold can be represented in a coordinate system. (*c*) Example of a codified molecule, a catalpol scaffold, shown in both graphical (top) and vectorial (bottom) forms. Note that carbon C11 has a value of 3 since it is known that iridoid oxidase directly oxidizes C11 to an aldehyde without an alcohol intermediary, and thus, the aldehyde has a value of 1, carboxylic acid has a value of 2, and decarboxylation has a value of 3.

**Fig. 3.**
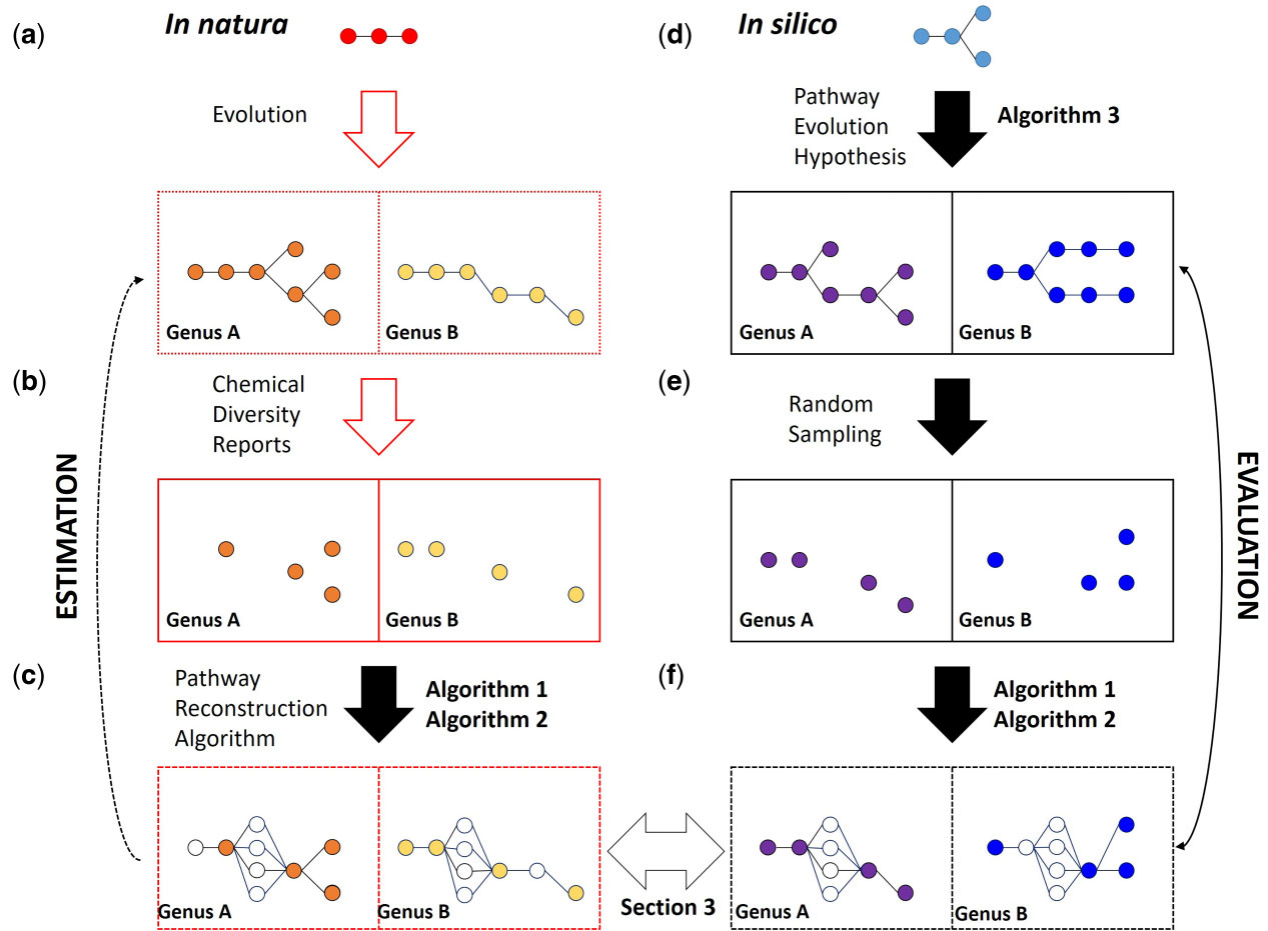
Overview of the strategy. (*a*) Metabolism in an ancestral organism, through evolution, expands, and differentiates into specialized pathways in different genera. In the case of iridoids, the sequence of biochemical steps of these pathways is unknown (dotted box). (*b*) Through natural product isolation and characterization, an incomplete subset of these molecules has been reported (color circles) in different genera. (*c*) Our objective, through Algorithms S1 and S2, Supplementary Material online (explained in Section 1), is to accurately estimate these pathways represented in panel (a) using reported (color) and predicted molecules (white circles). For that, we need five parameters for Algorithms S1 and S2, Supplementary Material online that must be calculated. We cannot optimize these parameters because we cannot evaluate performance: even in the few characterized pathways, typically in just one species, it is virtually impossible to determine if an enzyme or metabolite is truly absent or just has not been reported. Thus, we simulate these processes in silico. (*d*) We take an initial pathway in the iridoid chemical space and apply the evolutionary processes that we hypothesize explain the emergence of iridoid chemical diversity (outlined in Section 2). Algorithm S3, Supplementary Material online then outputs a computed pathway for each genus (solid box). (*e*) We take a random sample of the molecules in these calculated pathways. (*f*) We then apply Algorithms S1 and S2, Supplementary Material online with a wide range of parameters to reconstruct the pathways. We can compare these results to the original pathways in (*d*) and choose the parameters that offer the best reconstruction (as we do in Section 4). In Section 5, we apply these parameters to the original data set in (*b*) to get the pathway hypotheses that best estimate the original, unknown pathways in (*a*). Finally, in Section 6, we use these hypotheses to facilitate enzyme discovery.

To mathematically represent these oxidation reactions such that the biochemical steps can be represented in the pathway reconstruction algorithm, we codified the iridoid scaffold using an ordinal system to describe the oxidation step of each of the carbons. As shown in fig. 2*a*, this notation starts at a saturated carbon (value of 0), followed by alcohol (1), then aldehyde (2), carboxylic acid (3), and finally decarboxylation (4), with the exception of C11, which is known to bypass the alcohol intermediate (see C11 in fig. 2*c*; Miettinen et al. 2014). In this representation, each carbon is an independent dimension (fig. 2*b*), and the Manhattan distance between pairs of molecules is directly related to the minimal number of reactions needed to convert one into the other. Double bonds and epoxide rings, however, affect more than one carbon and can coexist with further modifications as long as valence is respected (fig. 2*a*). Thus, we developed an expanded notation to represent these possibilities: a carbon having a value of *i*/4 if it is involved in a double bond (see C3 and C4 in fig. 2*c*) and 3*i*/4 if it is involved in an epoxide ring (see C7 and C8 in fig. 2*c*). With this complete representation, a generalized calculation of Manhattan differences, which becomes vectorial in the complex plane, yields an approximate value of 1 in reaction–adjacent molecules (Appendix S1, Supplementary Material online). Whereas this function loses the properties of topological distance, by exclusively connecting molecules with differences of 1 in a graph, the geodesic distance is related to biosynthetic distance. This representation allows us to define a space of 6.24 × 10^4^ iridoid scaffolds, connected by 8.22 × 10^5^ reactions, in which we can place the vast majority of reported iridoid scaffolds (supplementary file 2, Supplementary Material online).

The pathway reconstruction algorithm works by taking the 64 unique chemical scaffolds that have been reported in Lamiaceae as points in space, applying to each a number *n* of reactions and then calculating the distance between all these points, connecting pairs of molecules with a distance of one by an edge in a graph (see Algorithm S1 and supplementary fig. S1, Supplementary Material online). We call this set of networks, one for each genus, the *naïve prediction*, as it does not consider the phylogenetic relationships of the genus in which the molecules were reported (Algorithm S1, Supplementary Material online).

By considering phylogeny, we can refine this naïve prediction by eliminating implausible connections between the calculated chemical structures. We do this by calculating scores to each one of the metabolites in the naïve prediction, by multiplying the report or prediction matrix of the molecules by genus, to either the phylogenetic correlation (Martins and Hansen 1997; Garland and Ives 2000) or Felsenstein’s weights at the root (Felsenstein 1985a, 1985b) (Appendix S2, Supplementary Material online). This concept is extended verbatim to weigh the edges in the graph or both the metabolites and the edges. The pathways within a percentage of the maximum sum of scores (set by the user as *tolerance*) are then chosen, and the rest are discarded (Algorithm S2, Supplementary Material online). We call this set of phylogenetically pruned biosynthetic route predictions *pathway hypotheses*.

Therefore, to arrive at a biosynthetic pathway hypothesis from a set of reported molecules, we need to provide the algorithms with five parameters: the number of reactions to apply to reported molecules; whether to calculate scores based on the reported or the predicted metabolites; whether to use Felsenstein’s weights or phylogenetic correlation to weight vertices (metabolites), edges (reactions) or both; and which tolerance to use as a cutoff.

In nature, metabolic pathways expand and diverge in different genera, creating chemical diversity (fig. 3*a*). This diversity has been proved by natural product isolation and characterization, creating an incomplete subset of reports of molecules in different species (fig. 3*b*). For the pathway reconstruction algorithm to accurately predict biosynthetic pathways from these reports, values for five parameters must be determined (fig. 3*c*). To model natural phenomena when only sparse measurements are available, synthetic data sets are typically used to optimize reconstruction parameters. This practice has been used, for example, to train plant phenotyping algorithms (Ubbens et al. 2018; Toda et al. 2020), model transcriptional regulatory networks (Van den Bulcke et al. 2006), and alternative RNA splicing (Rosenberg Alexander et al. 2015); the latter outperforms models trained with real data in predicting in vivo experimental results (Nikolenko 2021). We thus set to develop a synthetic data set to calculate optimal parameters for Algorithms S1 and S2, Supplementary Material online and model the evolution of chemical diversity in silico (fig. 3*d–f*). For that, we first set the basis of an actionable pathway evolution hypothesis (Section 2) and select the outputs that are closer to the in natura pathways (Section 3). We can then optimize the parameters of the pathway reconstruction algorithm using that data set (Section 4). We predict the iridoid pathways using these optimized parameters in Section 5 and use these predictions to facilitate gene discovery in Section 6.

### Section 2: Pathway Evolution Hypothesis

First, we need to develop a model for iridoid pathway evolution. Iridoids act on generalist herbivores (Dobler et al. 2011), suggesting that the selective pressures impacting their diversification are not directed against a specific molecular target and thus do not follow structure refinement. Instead, they suffer extensive derivatizations to escape degradation and sequestration (Dobler et al. 2011; Kries et al. 2017). In other words, since there is no singular target, the entire iridoid set is responsible for the overall bioactivity and likely follows “interaction diversity”-driven evolution (Berenbaum and Zangerl 1996; Iason et al. 2011; Gershenzon et al. 2012) in a similar fashion to phenolic compounds (Whitehead et al. 2021). We hypothesize that this diversity releases singular iridoids from strong selective pressure, allowing inactive intermediaries to exist without an immediate evolutionary advantage, analogous to the neutral theory of molecular evolution.

We developed an actionable pathway evolution hypothesis to recreate biosynthetic pathway evolution of iridoid biosynthesis. The pathway evolution hypothesis follows three practical constraints: random uniform exploration of the chemical space by novel enzyme activity followed by fixation on the substrate present, analogous to Granick’s hypothesis (Granick 1957); an enzyme diversity threshold as the main constraint to pathway expansion; and, finally, neutral selective pressure, leading to “molecular clock” dependency.

For the first constraint, we assume that there is a “biochemical reservoir” of enzymes that generates different catalytic activities that appear and disappear in evolutionary time and is only fixed in a species when they have a substrate to act upon (Granick 1957; Huang et al. 2016; O'Donnell et al. 2021). We assume that no immediate evolutionary pressure is exerted in this process, by virtue of the diversity-driven nature of iridoids, and thus, our algorithm samples the edges (reactions) of the iridoid chemical space randomly and uniformly. The second constraint of the algorithm assumes that the main limiting factor to the expansion of chemical space is enzyme diversity. In our hypothesis on pathway evolution, we assume that organisms have an arbitrary upper limit on the number of enzymes, so once a new enzyme is added, an existing enzyme, selected at random, must disappear. Finally, due to our neutral selective pressure hypothesis, we assume that new functions appear at a constant rate that depends on the “evolutionary clock”. Thus, the furthest away a species is from the common ancestor, the more the rounds of catalytic exploration happen, proportional to the length of the branch in the phylogenetic tree.

The implementation of these assumptions is shown in Algorithm S3, Supplementary Material online, where we establish a function that applies the pathway evolution hypothesis to the phylogenetic tree shown in fig. 1 and an “ancestral pathway” and outputs a pathway for each genus in the tree that has evolved from a common ancestor, following the above-mentioned constraints. The user-determined parameters are as follows: the number of reactions (edges) that the ancestral pathway has, the maximum number of enzymes allowed in the pathway, and the number of new catalytic activities (rounds of exploration) per unit of distance in the phylogenetic tree.

### Section 3: Choosing Nature-like Pathways

If the hypothesis on pathway evolution reasonably mimics the evolution of iridoid diversity in the Lamiaceae, Algorithm S3, Supplementary Material online should generate, with the right constrains, in silico pathways that resemble extant iridoid pathways. Since iridoid biosynthesis has not been elucidated, we cannot compare the iridoid pathways (fig. 3*a*) directly with the in silico pathways generated by Algorithm S3, Supplementary Material online (fig. 3*d*). However, by applying Algorithm S1, Supplementary Material online to *natural* data (fig. 3*b*), we can get networks within which we expect the real biosynthetic pathways to be (fig. 3*c*). We can then comparethese to the results of Algorithm S1, Supplementary Material online applied to the in silico pathways (fig. 3*f*; *hollow arrow*). If the outputs using Algorithm S1, Supplementary Material online on the in natura data are similar to the outputs of the same algorithm using the in silico data, we can reasonably assume that their inputs (fig. 3*a* and *d*) are similar.

The user-determined constraints of Algorithm S3, Supplementary Material online are as follows: the number of reactions of the ancestral pathway, the maximum number of enzymes allowed, and the number of rounds of exploration per unit of distance in the phylogenetic tree. We estimated the initial values of these parameters as stated in Appendix S3, Supplementary Material online and used them as inputs of Algorithm S3, Supplementary Material online to generate 50 in silico pathway evolutions, each with 21 genera, for each of the combinations of parameters. Only a subset of the iridoids present in each genus is reported; consequently, to emulate this, we sampled the in silico pathway sets randomly. We estimated the natural data set sampling rate by dividing the number of reported metabolites over the number of metabolites in the naïve prediction (Appendix S3, Supplementary Material online). We then sampled the in silico pathways by randomly selecting 10–60% of the simulated metabolites, in 10% increments (Appendix S3, Supplementary Material online); this translates to each one of the in silico pathways being sampled 6 times. We then applied Algorithm S1, Supplementary Material online to each one of the sampled in silico pathways independently. We then calculated for each result a list of 16 network descriptors (supplementary table S1, Supplementary Material online), selected as stated in Appendix S4, Supplementary Material online. We concatenated these descriptors by genus, so each row represents all the genus pathways, with 336 columns. We deduplicated, log_10_-transformed, and *z*-scaled the matrix and applied a Uniform Manifold Approximation and Projection (UMAP) to reduce the dimensionality of this data set (supplementary fig. S2, Supplementary Material online).

If we project the *natural* naïve model to the UMAP space, the in silico sets that are the closest should be the ones that have the most similar pathway topologies. Since UMAP is not an exact, but a stochastic process, we projected the in natura naïve model 10,000 times and used the Mahalanobis distance to choose the closest ($\chi_{2}^{2}$ < 0.99) in silico sets (supplementary fig. S3, Supplementary Material online). This yielded a sample of 320 pathway evolution sets, each one consisting of 21 in silico pathways, one per genus, and the sampling percentage to which it was subjected.

### Section 4: Selection of Optimal Parameter Values for the Pathway Reconstruction Algorithms

We next use the in silico pathways that are most similar to the experimentally observed iridoid pathways to perform a model-based selection of optimal parameters of the pathway reconstruction algorithms, as first outlined in Section 1. To get a phylogenetically pruned model from a set of reported molecules, we need to provide Algorithms S1 and S2, Supplementary Material online with five parameters. We tried a combination of metabolite extension by 0, 1, and 2 reactions, and a tolerance from 0% to 100% in increments of 10%, as well as phylogenetic correlation and Felsenstein’s weights, and weighing metabolites, reactions or both, in predicted or measured events. This yields a total of 396 combinations evaluated in 320 surrogates, each with 21 genera. We resampled each combination 10 times, following their respective sampling percentage, for a total of 2.6 × 10^7^ evaluations. In this step, we compared the output in fig. 3*f* with the input shown in fig. 3*d*, as explained in Appendix S5, Supplementary Material online.

As shown in supplementary fig. S4, Supplementary Material online all the models have good performance estimators, and the model with no extra reactions added (supplementary fig. S4, Supplementary Material online black) has consistently better estimators in both enzymes and metabolites. To choose the best parameters to use with Algorithms S1 and S2, Supplementary Material online and the experimentally observed 274 iridoid structures, we decided to use Matthew’s correlation coefficient (Matthews 1975) to predict enzymes and metabolites (supplementary fig. S5, Supplementary Material online). It is important to note that the models with one and two extra reactions are often the same (supplementary fig. S5, Supplementary Material online blue and red) as for the vast majority of the surrogates, as well for in natura data, metabolites are no more than two reactions away from a connected node. Thus, as long as some form of pruning is applied to the model (tolerance < 100%), the result of the models are effectively the same. The best performance was obtained by weighing reported metabolites by phylogenetic correlation, and a tolerance of 10%, for the no extra reaction model, and Felsenstein’s weights at the root, with no pruning tolerance, to expansions by one and two reactions. The performance parameters are shown in table 1 for the prediction of metabolites and table 2 for enzyme predictions.

**Table 1.** Diagnostic Ability of the Selected Models on Metabolite Predictions (on the left are shown the optimized parameters of Algorithms S1 and S2, Supplementary Material online, and on the right are shown the results on their predictions. The values of the evaluation are shown as the median with the lower and upper quartiles [25th and 75th percentile] shown below, in parentheses. TPR, true positive rate [also called sensitivity or recall]; FPR, false positive rate [specificity]; FOR, false omission rate; PPV, positive predictive value [precision]; and MCC, Matthew’s correlation coefficient).

Chosen parameters					Evaluation of the predictions				
Reactions added	Calculate scores on	Weights	Applied on	Tolerance (%)	TPR	FPR	FOR	PPV	MCC
0	Reported metabolites	Phylogenetic correlation	Metabolites	10	0.875 (0.75,1)	0.025 (0,0.0477)	0.0149 (0,0.04)	0.8 (0.667,1)	0.808 (0.697,0.903)
1	Reported metabolites	Felsenstein’s weights	Metabolites	0	0.8 (0.667,1)	0.03125 (0,0.0652)	0.0261 (0,0.0556)	0.75 (0.6,1)	0.739 (0.576,0.877)
2	Reported metabolites	Felsenstein’s weights	Metabolites	0	0.8 (0.667,1)	0.03125 (0,0.0654)	0.0263 (0,0.0556)	0.75 (0.6,1)	0.739 (0.575,0.877)

**Table 2.** Diagnostic Ability of the Selected Models on Enzyme Predictions (on the left are shown the optimized parameters of Algorithms S1 and S2, Supplementary Material online, and on the right are shown the results on their predictions; the values of the evaluation are shown as the median [top] with the lower and upper quartiles [25th and 75th percentile] shown below, in parentheses. TPR, true positive rate [also called sensitivity or recall]; FPR, false positive rate [specificity]; FOR, false omission rate; PPV, positive predictive value [precision]; and MCC, Matthew’s correlation coefficient).

Chosen parameters					Evaluation of the predictions				
Reactions added	Calculate scores on	Weights	Applied on	Tolerance (%)	TPR	FPR	FOR	PPV	MCC
0	Reported metabolites	Phylogenetic correlation	Metabolites	10	0.824 (0.714,0.92)	0.0507 (0.0286,0.0847)	0.0259 (0.0102,0.0537)	0.706 (0.615,0.789)	0.714 (0.603, 0.806)
1	Reported metabolites	Felsenstein’s weights	Metabolites	0	0.706 (0.591, 0.81)	0.0472 (0.0247,0.0818)	0.0437 (0.0231,0.0769)	0.692 (0.563,0.813)	0.64 (0.498,0.765)
2	Reported metabolites	Felsenstein’s weights	Metabolites	0	0.706 (0.591,0.81)	0.0476 (0.025, 0.0824)	0.0437 (0.0231, 0.0769)	0.692 (0.563,0.813)	0.64 (0.497,0.765)

As shown in tables 1 and 2, in 75% of the sampled in silico cases, more than two-thirds of the metabolites and more than 61.5% of the enzymes predicted by the pathway hypotheses with the chosen parameters are indeed true positives (PPV). Similarly, in 75% of the in silico cases, less than one-quarter of metabolites and less than 28.6% of the enzymes that should be in the pathway were not predicted by the pathway hypothesis (tables 1 and 2, 1-TPR). Thus, if our model of evolution and sample selection reasonably mimics our data set on iridoids, we still expect a small percentage of metabolites and enzymes to be left out of the pathway hypotheses, which would still contain a significant amount of spurious results. However, the latter is expected to be further reduced when an ancestral pathway is calculated, to the detriment of the former.

### Section 5: Hypothesis on the Evolution of Iridoid Glucoside Diversity in Lamiaceae

**Fig. 4.**
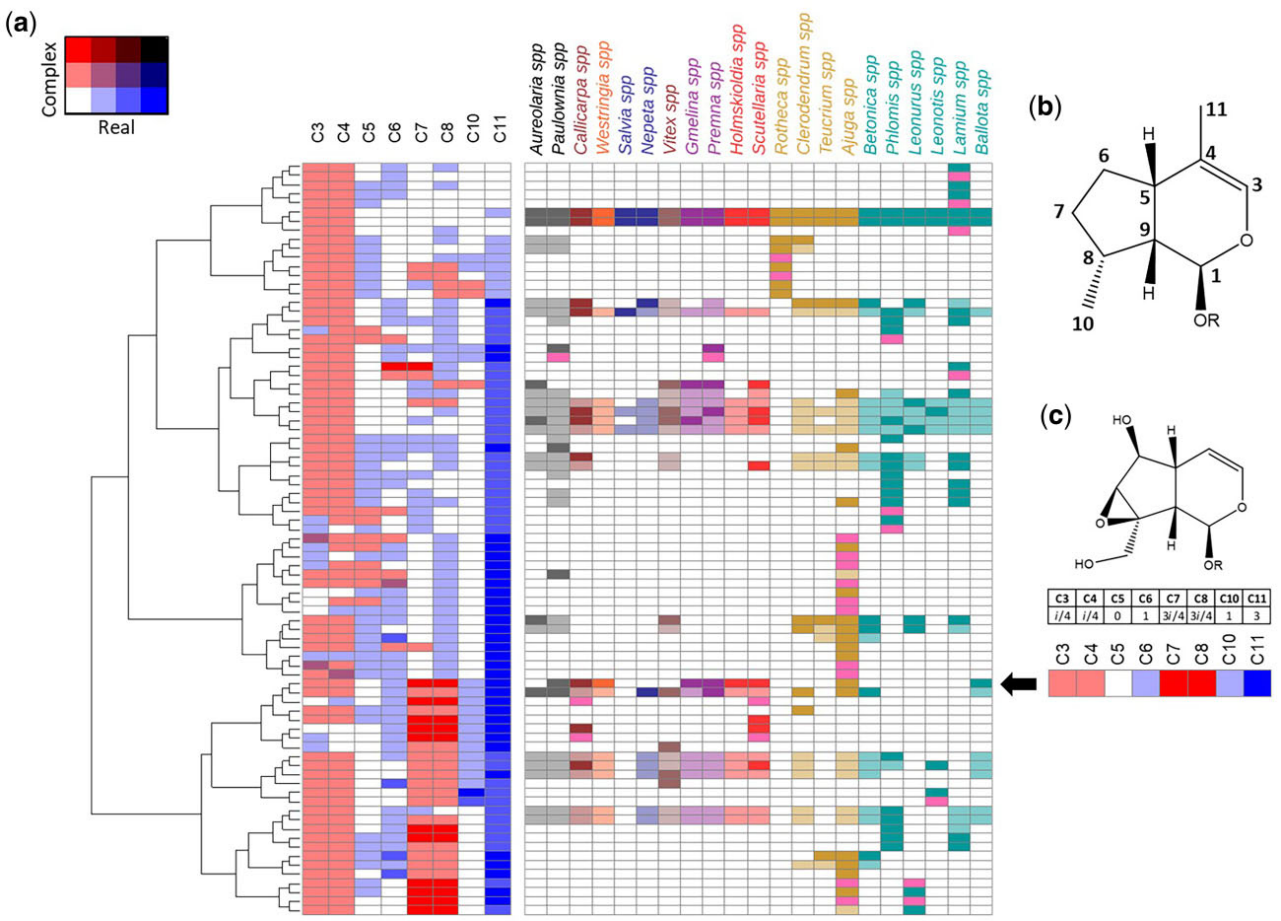
Iridoid scaffold chemical diversity. (*a*) Heatmap showing the clustering by the chemical distance of iridoids (left) and their reports in the selected genera (right). In solid colors, reported metabolites for the corresponding genus are shown; in semitransparent colors, predicted metabolites, reported in Lamiaceae, are shown; in pink, theoretical metabolites, not reported in Lamiaceae, are shown. The color code corresponds to the clade each genus belongs to, as specified in Fig. 1. (*b*) Numbering of the carbons in the scaffold, corresponding to the columns of the heatmap. (*c*) Example of how catalpol looks like a row in the heatmap; the arrow points at its position. Supplementary figs. S6–S24, Supplementary Material online show the chemical representations and predicted biosynthetic pathways.

We applied the best performing model with no expanded reactions, as described in Section 4, to the reports of iridoid diversity in natura (274 experimentally reported structures) and only applied the expansion by one reaction, along with the corresponding best parameters mentioned in Section 4, to include molecules with no immediate connection to another reported molecule. The resulting model contains an individual pathway hypothesis for each of the 19 Lamiaceae genera considered (shown in detail in supplementary figs. S6–S24, Supplementary Material online), which adds 23 predicted intermediaries to the 64 unique scaffolds and reduces the solution space to 122 putative reactions. By placing all predicted and reported scaffolds in the iridoid chemical space and clustering by geodesic distance, certain chemotaxonomic patterns become apparent (fig. 4). Namely, the Lamioideae clade has a high diversity of hydroxylated, nondecarboxylated iridoids, including a unique set of iridoids with a saturated C11 (*Lamium spp.*) In contrast, Ajugoideae has highly oxidized decarboxylated iridoids (*Ajuga spp.*) and a unique set of aldehyde iridoids (*Rotheca spp.* and *Clerodendrum spp.*; fig. 4). These two clades, Ajugoideae and Lamioideae, hold the majority of the iridoid scaffold chemical diversity.

**Fig. 5.**
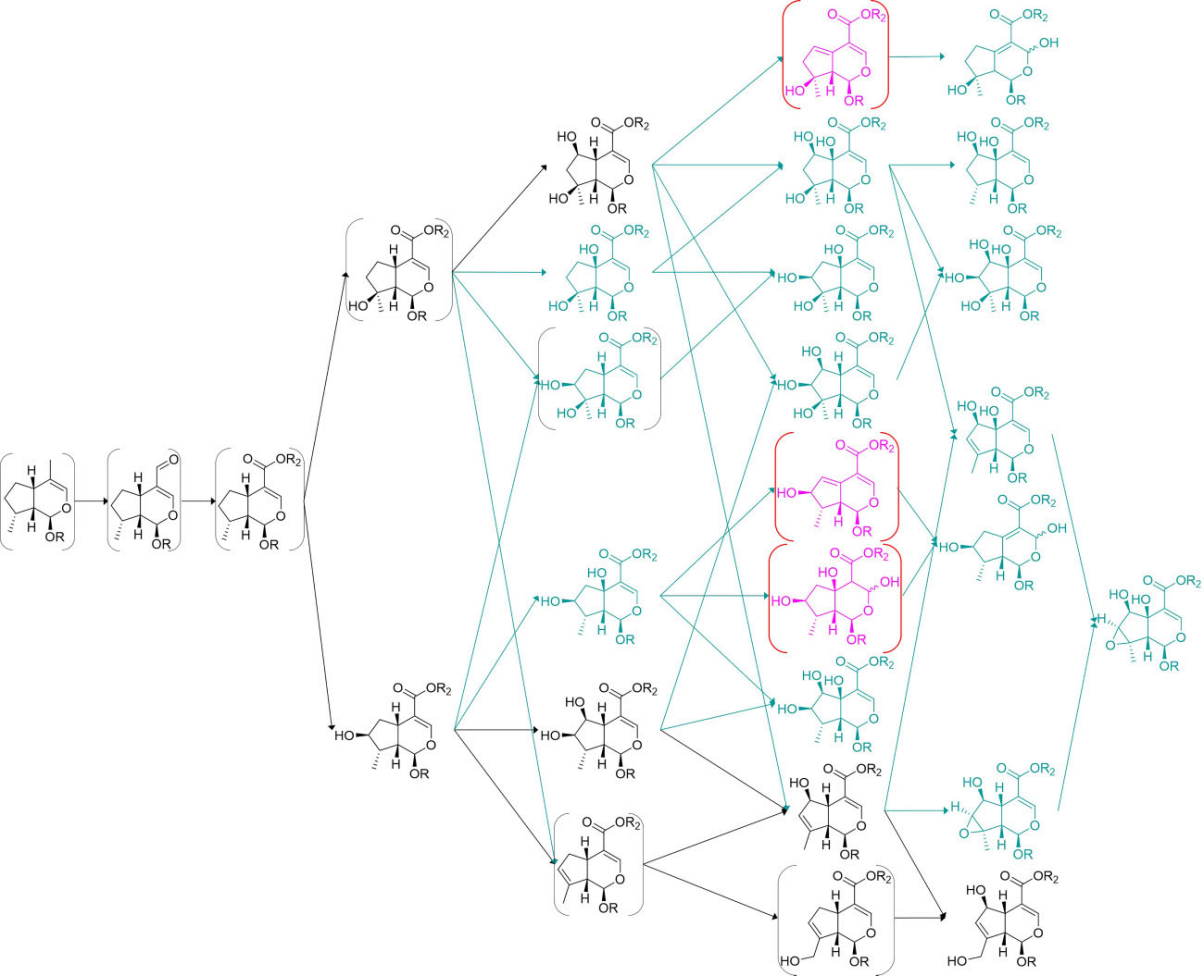
Iridoid pathway hypothesis for *Phlomis* spp. (Lamioideae). The metabolites and reactions expected to be present in the ancestral pathway are shown in black; metabolites reported in this genus, but not expected to be ancestral, are shown in Persian green; and completely theoretical metabolites are shown in pink. Metabolites predicted by our model, but not reported in the genus, are shown in brackets.

**Fig. 6.**
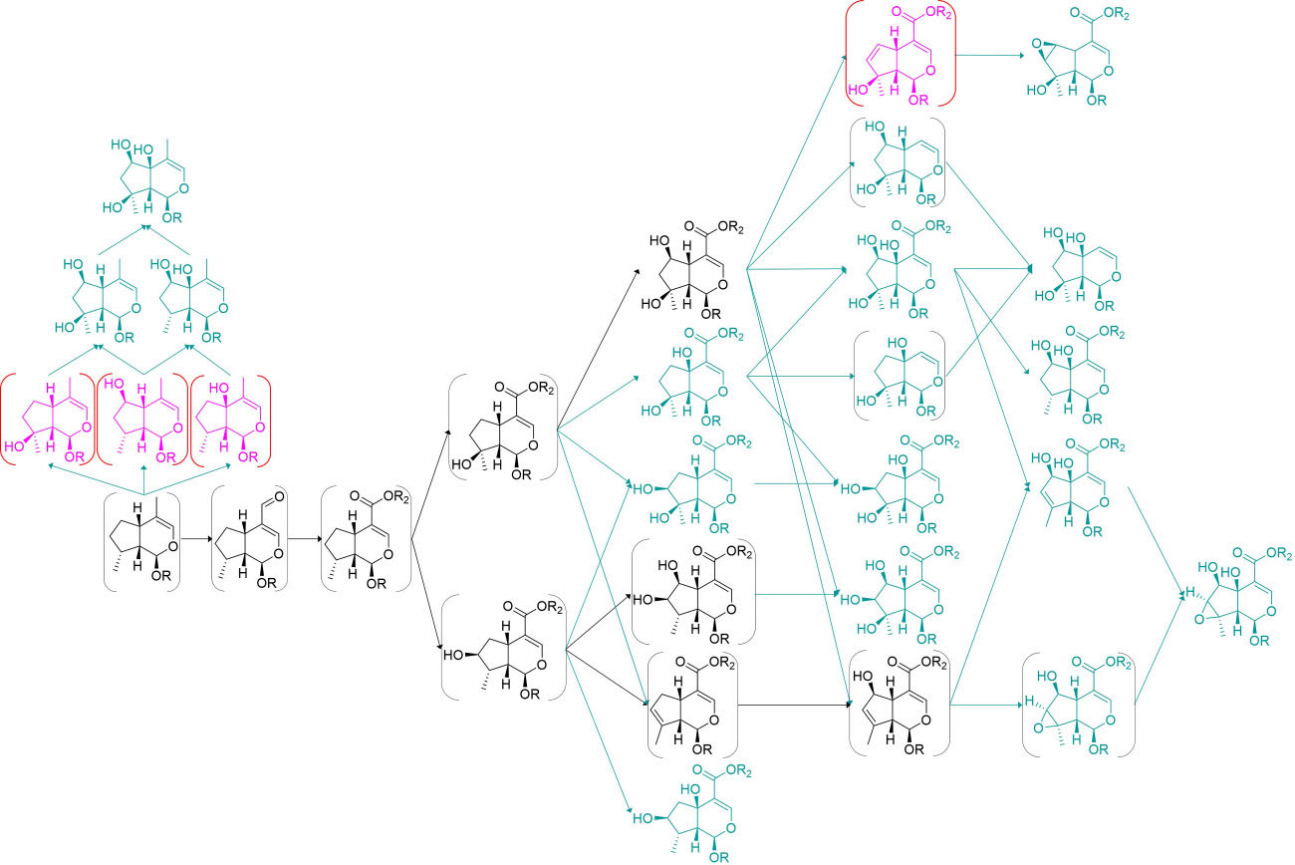
Iridoid pathway hypothesis for *Lamium* spp. (Lamioideae). The metabolites and reactions expected to be present in the ancestral pathway are shown in black; metabolites reported in this genus, but not expected to be ancestral, are shown in Persian green; and completely theoretical metabolites are shown in pink. Metabolites predicted by our model, but not reported in the genus, are shown in brackets.

**Fig. 7.**
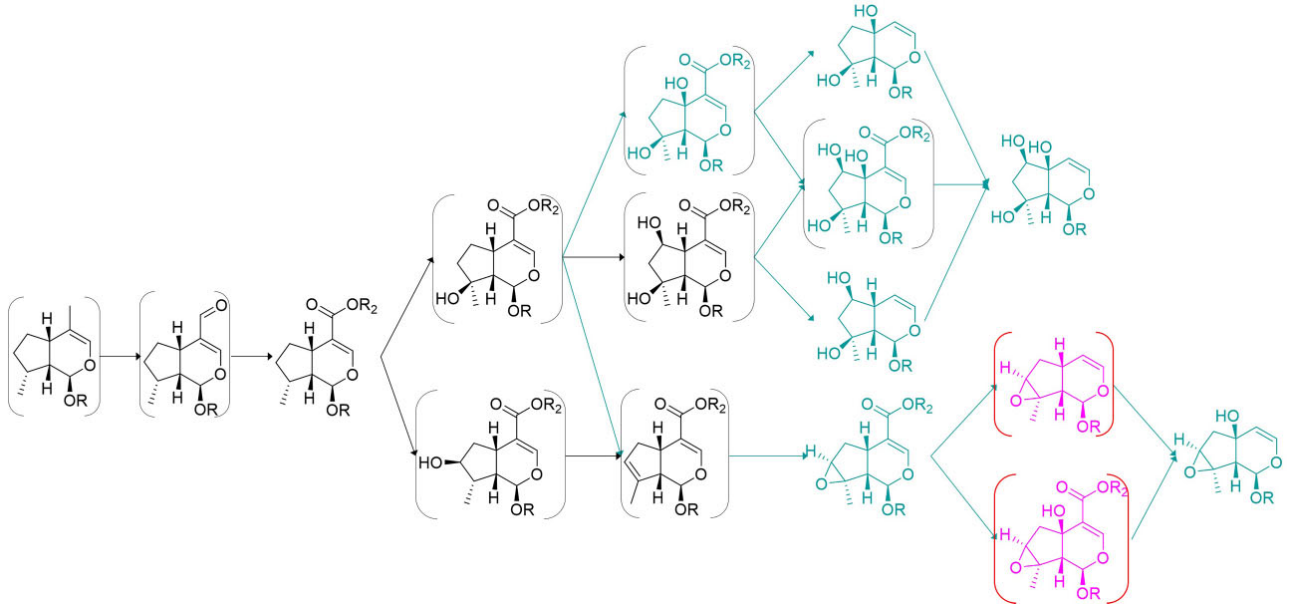
Iridoid pathway hypothesis for *Leonurus* spp. (Lamioideae). The metabolites and reactions expected to be present in the ancestral pathway are shown in black; metabolites reported in this genus, but not expected to be ancestral, are shown in Persian green; and completely theoretical metabolites are shown in pink. Metabolites predicted by our model, but not reported in the genus, are shown in brackets.

**Fig. 8.**
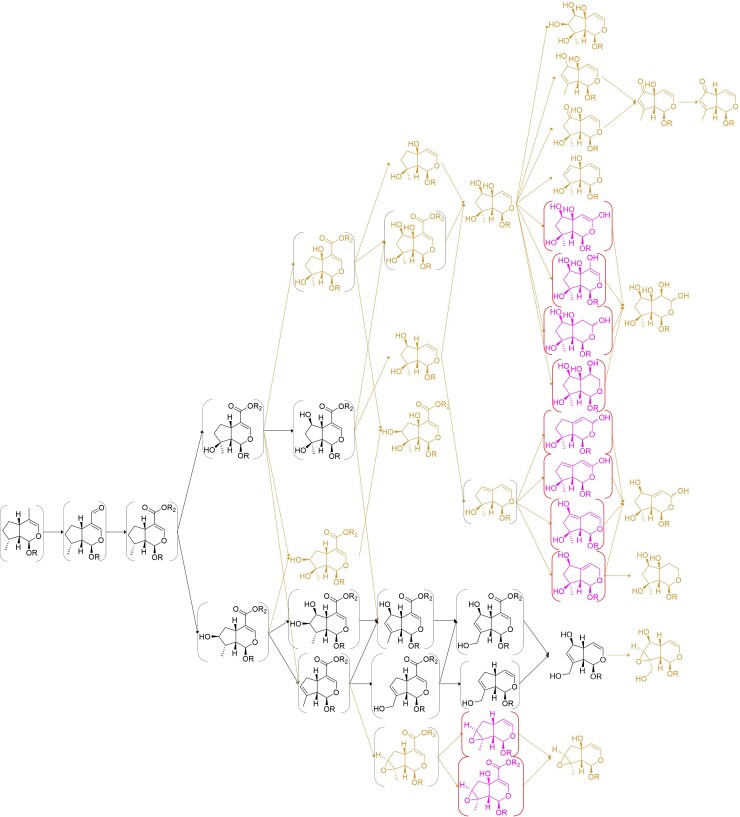
Iridoid pathway hypothesis for *Ajuga* spp. (Ajugoideae). The metabolites and reactions expected to be present in the ancestral pathway are shown in black; metabolites reported in this genus, but not expected to be ancestral, are shown in tussock yellow; and completely theoretical metabolites are shown in pink. Metabolites predicted by our model, but not reported in the genus, are shown in brackets.

Analyzing the pathway hypotheses for each species in these two clades, the diversity of carboxylate iridoids in the Lamioideae is predicted to be mostly due to emergent oxidation of the mussaenosidic and 8-*epi*-loganic acid scaffolds in *Phlomis, Lamium*, and *Leonurus* genera (Figs. 5–7). In contrast, the diversity of decarboxylated iridoid scaffolds in *Ajuga spp.* is predicted to derive almost entirely from modifications of the harpagide scaffold (fig. 8). Interestingly, the pathways of saturated iridoids in *Lamium* spp. and aldehyde iridoids in the Ajugoideae are not predicted to be reincorporated in either genus to the main iridoid pathway (derived from the 7-deoxyloganic acid scaffold), which implies that once they enter these pathways, iridoid oxidase cannot act on them.

**Fig. 9.**
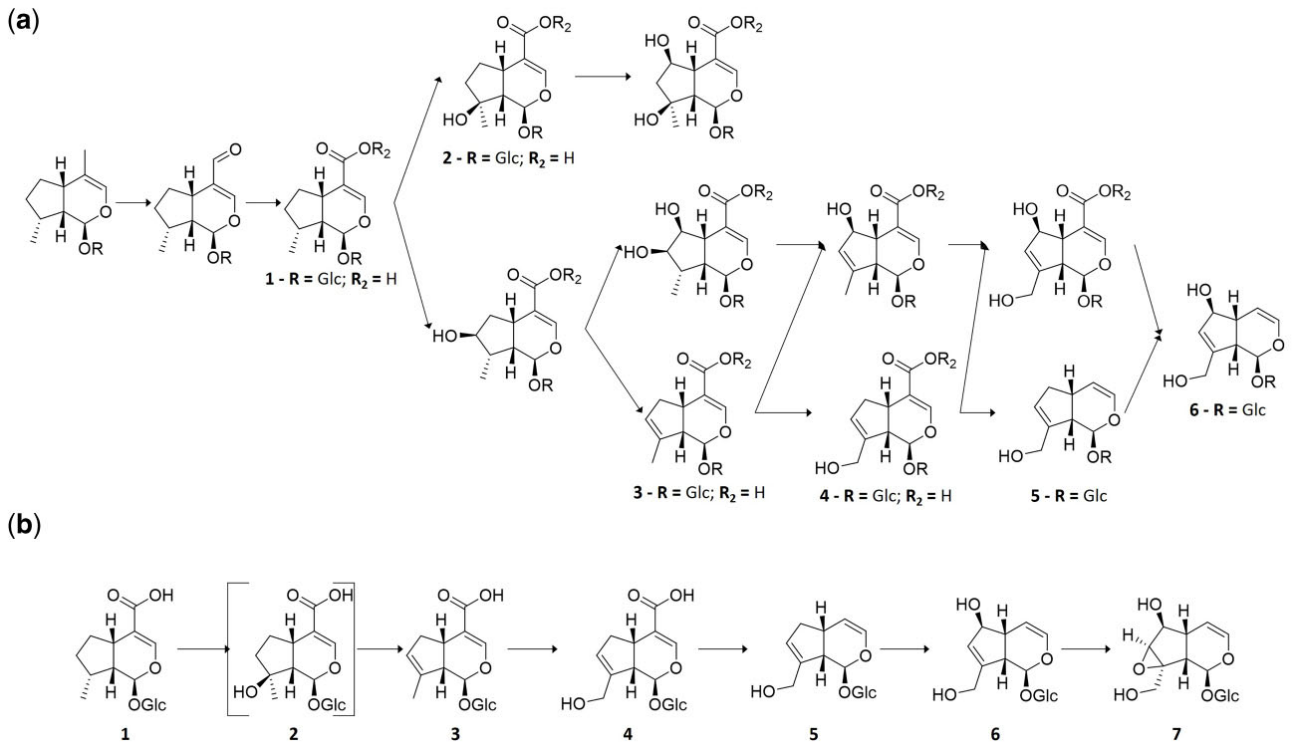
Pathway hypotheses. (*a*) Ancestral pathway predicted in the current work. (*b*) Damtoft–Jensen hypothesis (route II) is largely contained within this prediction. Mussaenosidic acid (in square brackets) is not predicted by our algorithm but is not definitively indicated by the labeling studies (Damtoft et al. 1993; Damtoft 1994). (**1**) 8-*epi*-7-deoxyloganic acid, (**2**) mussaenosidic acid, (**3**) 10-deoxygeniposidic acid, (**4**) geniposidic acid, (**5**) bartsioside, (**6**) aucubin, and (**7**) catalpol.

As shown in fig. 4, there is a subset of molecules that is either reported or predicted to be in most of Lamiaceae, which points to a highly conserved core iridoid pathway in the diverse species. Given the nature of the data set, in which lack of reports of a molecule does not indicate that the molecule is absent in the species, we decided to apply Felsenstein’s weights at the root to the individual pathways (edges and nodes) and kept those that had a cumulative score greater than 0.9. This method of estimation does not evaluate negatively unreported molecules and prioritizes a robust estimate of the ancestral pathway rather than a comprehensive one (fig. 9*a*).

Rigorous isotope labeling experiments led to the hypothesis of a proposed biosynthesis of aucubin and catalpol (Damtoft–Jensen hypothesis or “route II”) in Lamiaceae as well as Scrophulariaceae and families that has not been tested in 25 years (Damtoft 1981, 1994; Damtoft et al. 1983, 1993; Jensen 1991; fig. 9*b*). The results of these labeling experiments were not included in our algorithms, but the biochemical steps of the Damtoft–Jensen hypothesis are predicted by our model in all the Lamiaceae species that are predicted to have either aucubin or catalpol.

### Section 6: Discovery of *C. americana* CYP72 as an Aucubin Synthase

**Fig. 10.**
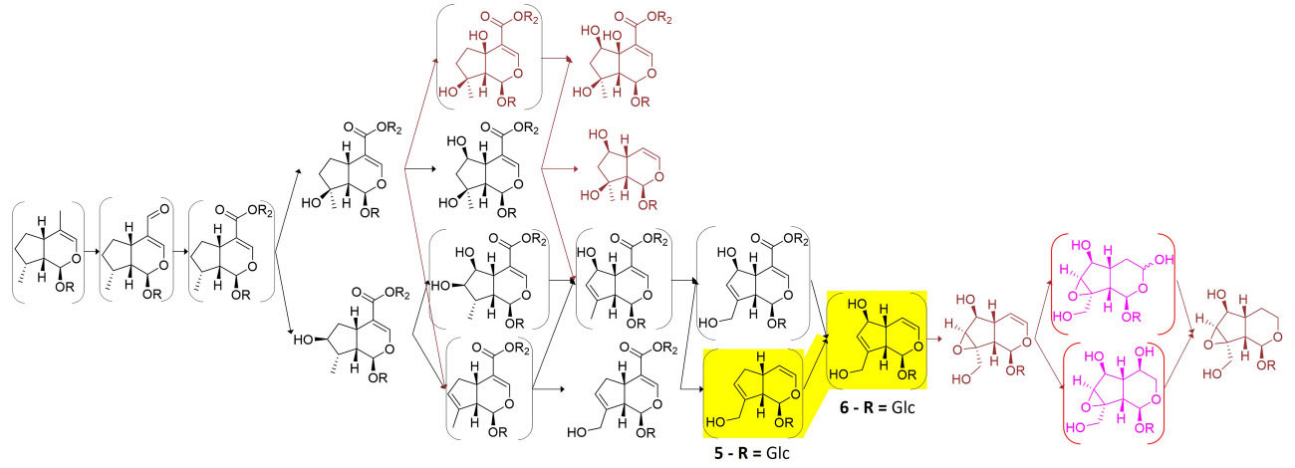
Iridoid pathway hypothesis for *Callicarpa* spp. (Callicarpoideae). The metabolites and reactions expected to be present in the ancestral pathway are shown in black; metabolites reported in this genus, but not expected to be ancestral, are shown in crail red; and completely theoretical metabolites are shown in pink. Metabolites predicted by our model, but not reported in the genus, are shown in brackets. Highlighted in yellow, the enzyme activity we discovered (CaAS), oxidizing (**5**) bartsioside to (**6**) aucubin, none of which have been reported in *Callicarpa* but were predicted to be in the ancestral pathway.

One of the most important applications for these predicted pathways is to use them as the basis for biosynthetic gene discovery. Most plant pathway elucidation relies on the careful functional characterization of hundreds of gene candidates, all of which must be evaluated for biochemical function. Having a hypothetical pathway in place against which to evaluate gene candidate function is an essential first step. As a proof of concept to evaluate our biosynthetic models, we chose *C. americana*, which has the highest impact on the prediction of ancestral conditions (supplementary fig. S25b, Supplementary Material online; fig. 10). We also chose *Lamium album* as a contrast to *C. americana*, as it is in the group with the highest phylogenetic correlation and the lowest Felsensteins weights (supplementary fig. S25a, Supplementary Material online).

To compile a list of enzyme candidates, we followed a “guilt-by-association” approach, in which we assume that genes in the same biosynthetic pathway should have similar expression profiles. For that, we analyzed RNA-seq expression profiles for both species, consisting of mature and young leaves, stems, petioles, roots, closed buds, and open flowers for *L. album* and *C. americana*, the latter also having expression from fruit tissue (Mint Evolutionary Genomics Consortium 2018; Hamilton et al. 2020). We then identified enzyme candidates for the MEP and early iridoid pathway (from DXS to iridoid oxidase) by blasting known genes encoding known enzymes against each transcriptome. Via a self-organizing map (SOM), we assigned all transcripts to 400 coalescing neurons and clustered these neurons via Ward’s algorithm of hierarchical clustering using the Manhattan distance between their type expressions. Finally, we generated the gene candidate list by choosing the contiguous cluster that has most of the early iridoid biosynthetic genes, as shown in supplementary fig. S26a, Supplementary Material online for *C. americana* (4,542 candidates in 2,850 orthologous groups) and supplementary fig. S26b, Supplementary Material online for *L. album* (4,613 candidates in 2,769 orthogroups). Within these candidate genes, only 520 orthologous groups were shared (supplementary fig. S26c, Supplementary Material online), less than 25% in each species, greatly reducing the number of candidates. We reasonably assume that enzymes that catalyze reactions in the ancestral pathways should belong to the same orthogroups, even if these enzymes have been repurposed.

**Fig. 11.**
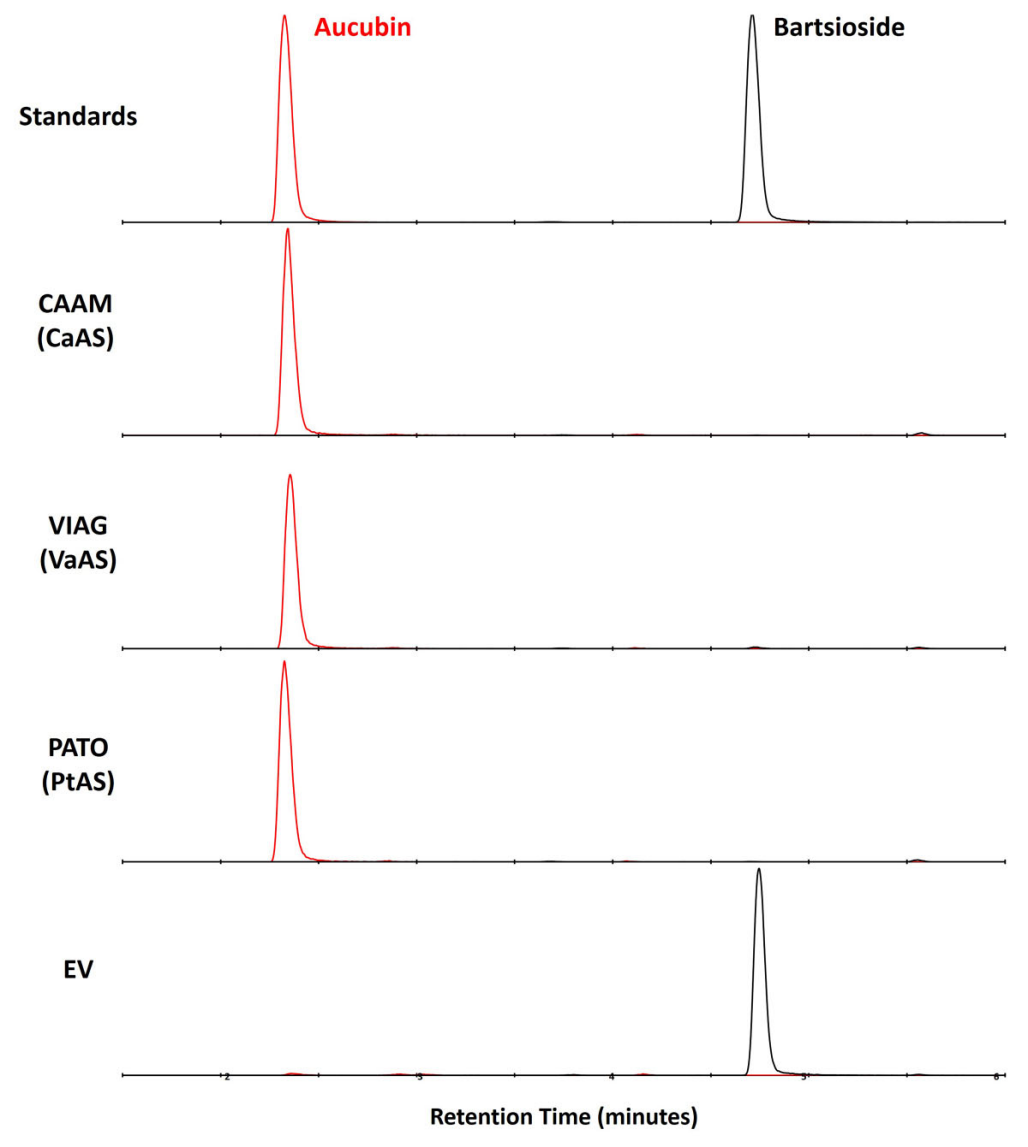
XICs of microsome incubations with bartsioside. Two channels are depicted, corresponding to the most abundant adducts of bartsioside (black, [M + FA-H]^−^ = 375.1291 ± 0.05) and aucubin (red, [M + FA-H]^−^ = 391.1241 ± 0.05). Intensities are scaled to the highest intensity of the corresponding channel in all incubations. Chromatograms are shown for microsomes containing the enzyme candidates of *C. americana* (CAAM), *V. agnus-castus* (VIAG), and *P. tomentosa* (PATO), as well as the empty vector (EV) control. Since enzyme candidates oxidize bartsioside to aucubin, they were renamed as aucubin synthase for each species (CaAS, VaAS, and PtAS).

We further assumed that hydroxylation is catalyzed by cytochrome p450s, so we further cleaned these candidates and kept only ten orthogroups, which had at least one member assigned as a p450 by *pfam* annotation (supplementary fig. S26d, Supplementary Material online). This reduced the list to 24 candidates from *C. americana* and 12 from *L. album*. Upon visual inspection of UniProt annotations of the transcripts belonging to each of these orthogroups, we noticed that one orthogroup (OG0001031) contained four enzymes (three in *C. americana*) that had Uniprot annotations belonging to the CYP72 family. Since previously reported enzymes that act on iridoid substrates, albeit with different C8 stereochemistry, have been reported to have high homology or belong to the CYP72 family (Irmler et al. 2000; Salim et al. 2013; Rodríguez-López et al. 2021), we decided to focus our efforts in this orthogroup. We tested these enzymes with a selected battery of metabolites predicted to be part of the ancestral pathway (see Materials and Methods section), even if they had not been reported in *Callicarpa* spp., and found that one of them, Calam.02G160400.1, produced aucubin when incubated with bartsioside (fig. 11). This enzyme was thus renamed *Callicarpa americana* aucubin synthase (CaAS). Neither bartsioside nor aucubin is reported in *Callicarpa* spp., yet both molecules, as well as the reaction connecting them, are predicted by our model to be in the genus and the ancestral pathway.

Notably, bartsioside has only been reported twice in Lamiaceae, in *Vitex grandifolia* (Bello et al. 2018) and *Sideritis romama* (Venditti et al. 2016), the latter being absent in our data set. Nevertheless, the clade in which we found CaAS (supplementary fig. S27, Supplementary Material online bold, red color) seems to be enriched in genera that are predicted by our model to produce aucubin from bartsioside (bold). To find further support for our hypothesis, we assayed orthologues from this clade belonging to *V. agnus-castus* (supplementary fig. S27 Supplementary Material online, VIAG_c41279_g1_i4), which belongs to the same genus as the only report of bartsioside in our data set, and the outgroup *P. tomentosa* (supplementary fig. S27, Supplementary Material online, PATO_c46653_g6_i1). In these species, aucubin is also predicted by the model to be produced from bartsioside. As shown in fig. 11, the orthologues from these species catalyzed the predicted reaction, further supporting the model and strengthening the hypothesis of this reaction being present in the ancestral pathway.

Here, we demonstrated the power of taking scattered phytochemical reports and, through an evolutionary framework based cheminformatics model, transformed them into robust metabolic pathway hypotheses. These are the first systematic hypotheses on the biosynthesis of *epi*-iridoid glucosides in 25 years and set the stage for streamlined work on the iridoids pathway. Particularly, these predictive models facilitated the discovery of an aucubin synthase, which is only the second enzyme reported for the *epi*-iridoid pathway. Notable work in the field has used comparative genomics (Edger et al. 2015), molecular evolution (Huang et al. 2016), information theory (Li et al. 2020), and experimental bioassays (Whitehead et al. 2021) to test hypotheses on the evolution of chemical diversity. The current work builds on the bases set by this research to develop a new approach, combining an evolutionary hypothesis with chemical information as means of formulating and training predictive models on metabolism.

## Conclusions and Limitations

The current approach to generate pathway hypotheses can be summarized in three steps: collect metabolite reports for different genera, connect these molecules via reactions, and prune the solution space by considering phylogenetic relationships between genera. This approach is explained in Section 1, and applied in Section 5, with a successful demonstration of its functionality in Section 6 with the discovery of aucubin synthase. Although this general approach can be applied to reconstruct different pathways, some aspects presented here are specific for iridoids and are not directly applicable to all metabolism. Namely, the mathematical representation of iridoids using an ordinal system with complex numbers takes advantage of the limited modifications that can occur to each carbon in the iridoids scaffold, partly as a result of the cyclopentanopyran fused ring system. Pathways that involve substantial rearrangements—such as those catalyzed by terpene synthases—would require a significantly modified model. Additionally, these iridoids contain only carbon, hydrogen, and oxygen; nitrogen-containing compounds would also require a modified model. Each specialized metabolic pathway will present its own peculiarities that will require different functional representations and may require different tools for connecting them via feasible reactions. Correspondingly, grouping by genus masks individual species chemotype variability: for example, although a pathway hypothesis is shown for the Nepeta genus, only *N. septemcrenata* has been reported to have iridoid glucosides with the stereochemistry considered in this manuscript; *N. cataria* and *N. musinnii* have completely different stereoisomers (Lichman et al. 2020).

Modeling requires a selection of parameters that fit the predictions to the natural phenomenon as closely as possible. Published data are sparse and only useful to evaluate true positives since the lack of a report does not provide any information on the absence of a metabolite or enzyme: they may exist in nature but are yet to be discovered. To avoid the drawbacks of using incomplete, published data, the current work generated a synthetic data set to select the best parameters of pathway reconstruction. A critical assumption is that iridoids follow an “interaction diversity”-driven evolution, which may be reasonably extended to many pathways, such as phenolic compounds (Whitehead et al. 2021), but is unlikely for other pathways, like glucosinolates (Edger et al. 2015). Similarly, our simulations rely on the exploration of a small (6.24 × 10^4^ scaffolds) defined chemical space, which is a property only found in certain pathways, like xanthine alkaloids (O'Donnell et al. 2021). A reformulation of the exploration steps of the algorithm would be needed for pathways with larger chemical spaces generated by substantial molecular rearrangements. Finally, within the explored parameters, Algorithm S3, Supplementary Material online yields pathways with meshed network topology, as expected for iridoids; for other pathways, like canonical monoterpenes (Mint Evolutionary Genomics Consortium 2018) or sesterterpenes (Huang et al. 2017), whose pathways are expected to have a radial topology, a different model would need to be developed.

In this work, we present a pathway reconstruction algorithm that, using evolutionary hypotheses and literature reports, generates predictive models of metabolism. In these pathway hypotheses, we still expect a small but significant percentage of false positives, as well as metabolites and enzymes being left out. As stated by Brown and Thomson (2018), “modeling is an exercise in explanation”: models simplify reality, provide insights, and generate testable predictions. The pathway hypotheses are models that organize numerous reports of iridoids into simplified biosynthetic routes, offer insights into the evolution of iridoid scaffolds diversity, and are a set of testable predictions that were successfully applied for enzyme discovery. In a similar fashion to phylogenetic trees, we expect this model to be continuously validated and revised by the scientific community, as new evidence is generated and novel methods are developed.

## Materials and Methods

### Expression and Transcriptome Data

*Lamium album* tissues (closed buds, mature leaves, open flowers, petioles, roots, stems, and young leaves) were ground in liquid nitrogen, and total RNA was extracted using the Qiagen Plant RNeasy kit (Qiagen, Germantown, MD, USA) following manufacturer’s instructions. RNA samples were treated with Ambion TURBO DNase (Thermo Fischer Scientific, Waltham, MA, USA) to remove any residual DNA in the sample. RNA integrity was assessed using an Agilent 2100 Bioanalyzer System (Agilent Technologies, Santa Clara, CA, USA) followed by Illumina TruSeq Stranded mRNA Library Preparation (Illumina, San Diego, CA, USA) using the Sciclone G3 NGS workstation (PerkinElmer, Waltham, MA, USA) by the Research Technology Support Facility at Michigan State University. Libraries were sequenced on an Illumina HiSeq 4000 (Illumina, San Diego, CA, USA) in 150 nt paired-end mode. Read quality was assessed using FastQC v0.11.5 (Andrews 2016) followed by removal of low-quality bases and adapters using Cutadapt v1.16 (Martin 2011) with the following parameters: –times 2, -m 20, –trim-n, and -q 20,20. Another round of Cutadapt was run to remove polyA tails using the parameters: -a/-A “A 100”, -g/-G “T 100”, -O 20, -m 20, and –times 2. After all cleaning, a total of 301.2 million read pairs (98.9%) remained. Since the total number of reads was over 300 million, normalization using Trinity v2.2.0 (Grabherr et al. 2011) was performed with the following options: –pairs_together, –max_cov 50, and –SS_lib_type RF. After normalization, 16.3 million read pairs (5.5%) remained and were input into Trinity v2.2.0 (Haas et al. 2013) using the following parameters: –min_kmer_cov 2, –min_contig_length 500, –SS_lib_type RF, and –group_pairs_distance 500. The transcriptome assembly was then filtered, retaining only the longest isoform per transcript, yielding a total of 48,160 transcripts. Finally, using CD-HIT-EST, part of the CD-HIT package v4.6.4 (Li and Godzik 2006; Fu et al. 2012), transcripts with greater than 95% identity were grouped together and only the longest transcript was retained. The final transcriptome assembly included 45,068 transcripts. Expression abundances were estimated for the seven tissues by first mapping the cleaned Illumina reads to the transcriptome assembly using TopHat2 v2.0.14 (Kim et al. 2013) with a minimum intron size of 5, maximum intron size of 10,000, fr-firststrand library type, and a minimum distance between the ends of a read pair (-r) of 300 followed by Cufflinks v2.2.1 (Trapnell et al. 2010; Roberts et al. 2011) with the following options: -I 10000, -G, -b, and fr-firststrand library-type. Functional annotation was assigned by running Blast (Altschul et al. 1990; States and Gish 1994; Zhang et al. 2000) against the Uniprot database (UniProt Consortium 2018) and recovering the highest scoring hit, with an *E*-value threshold of 1 × 10^−3^. Protein family annotation was performed using HMMER v3.1b2 (Eddy 2011, 2015) against PFAM v32 (Finn et al. 2015) database, with an *E*-value cutoff of 1 × 10^−3^.

*Callicarpa americana* transcriptome and expression abundances were obtained from published data (Hamilton et al. 2020) along with the transcriptomes of the other 46 Lamiaceae species and four outgroup species (Mint Evolutionary Genomics Consortium 2018). The *Antirrhinum majus* predicted proteome was obtained from the snapdragon genome assembly v2.0 (Li et al. 2019). Orthologous/paralogous groups were identified with OrthoFinder v0.7.1 (Emms and Kelly 2019) as previously described (Mint Evolutionary Genomics Consortium 2018), the only difference being the update of the *L. album* and *C. americana* transcriptomes and the addition of the *A. majus*-predicted proteins.

### Identification of Enzyme Candidates

To identify putative genes involved in the early iridoid pathways of *C. americana* and *L. album*, we blasted the transcriptome assemblies with sequences of previously reported enzyme candidates (Mint Evolutionary Genomics Consortium 2018) for 1-deoxy-d-xylulose-5-phosphate synthase (DXS), 1-deoxy-d-xylulose-5-phosphate reductoisomerase, 2-C-methyl-d-erythritol 4-phosphate cytidylyltransferase, 4-(cytidine 5′-diphospho)-2-C-methyl-d-erythritol kinase, 2-C-methyl-d-erythritol 2,4-cyclodiphosphate synthase, 4-hydroxy-3-methylbut-2-en-1-yl diphosphate synthase, geranylgeranyl pyrophosphate synthase large subunit (GGPPS-LSU), geraniol synthase, 8-hydroxygeraniol oxidoreductase, iridoid synthase, and iridoid oxidase. The best BLAST hits for each of these genes were selected manually and can be found in the EDMOND repository files, in the Biosynthesis.csv file.

To generate the list of candidate genes from *C. americana* and *L. album* expression data, for each separate species, we took the expression matrix, in fragments per kilobase per million mapped reads, removed zeroes by summing 1 to all the values, scaled by applying logarithm base 2, and generated a *z*-score subtracting the mean and dividing by the standard deviation in a transcript-wise manner. This yields a center-scaled matrix wherein every transcript has a mean of zero and a standard deviation of 1, allowing a comparison of expression patterns, regardless of the expression level. We decided to follow a “guilt-by-association” approach, in which we assume that enzymes involved in the iridoid pathway will have similar expression patterns to early pathway enzymes. Thus, we grouped transcripts by expression patterns in each species by means of a SOM using the *kohonen* library v3.0.10 (Wehrens and Kruisselbrink 2018), with a 20 × 20 grid and hexagonal geometry in a toroidal space. The codebook vectors of the neurons were clustered using Ward’s clustering criterion (Ward 1963; Murtagh and Legendre 2014) on Manhattan distances and divided into ten clusters. We used the above-mentioned best BLAST results for early pathway enzymes as bait and confirmed enrichment in the clusters by a hypergeometric test. Transcripts that were in the cluster enriched (*P* < 0.05) in early biosynthetic genes were considered to be coexpressed candidates. Only candidates with a matching orthogroup in both species and a pfam annotation corresponding to the cytochrome p450 family of enzymes were determined to be of interest, and Uniprot annotations were manually explored. Orthogroup OG0001031 contained four transcripts that had Uniprot annotations belonging to the CYP72 family, to which most of the discovered iridoid oxidizing enzymes belong (Irmler et al. 2000; Salim et al. 2013; Rodríguez-López et al. 2021) and was thus selected for further testing. The commented R code is available in the file Self-Organizing-Maps_CODE.r, along with all the necessary files, deposited in the folder named Gene_Candidate_Selection in the EDMOND database (see the Data Availability section).

### Heterologous Expression and Microsomal preparations

Candidate genes were synthesized by Twist Bioscience (San Francisco, CA, USA) and cloned into the Gal10 MCS of a pESC-Leu2d plasmid containing a Cytochrome P450 reductase (CPR) from *Artemisia annua* (Ro et al. 2008). To confirm the nucleotide sequence of CaAS, RNA was extracted from young leaves of *C. americana* plants from an independent batch than the published transcriptome data using the Qiagen Plant RNeasy kit (Qiagen, Germantown, MD, USA). cDNA was obtained by using SuperScript IV VILO MasterMix (Thermo Fischer Scientific, Waltham, MA, USA), and the target gene was extracted by PCR using primers that contained overhangs with homology to the pESC-Leu2d plasmid Gal10 MCS. The PCR product was gel-purified by the Zymoclean gel DNA recovery kit (Zymo Research Europe GmbH, Freiburg, Germany) and cloned using the ClonExpress II Kit (Nanjing Vazyme Biotech Co, Nanjing, China), and purified plasmids were sent for Sanger sequencing (GENEWIZ Germany GmbH, Leipzig, Germany). Sequences were found to match perfectly to the sequence extracted from the published transcriptome.

*Saccharomyces cerevisiae* YPL 154C:Pep4KO was used for heterologous expression, and microsomes were prepared as stated previously (Ro et al. 2002; Dang et al. 2017). Each batch of microsomes included an empty vector control, containing just the CPR, and a positive control, *Catharanthus roseus* 7-deoxyloganic acid hydroxylase. Microsome reactions were performed using 1 mg of total protein in 50 µl of 50 mM Tris buffer pH 7.4, supplemented with 20 µM substrate and 100 µM NADPH. Reactions were incubated overnight at 30°C in the dark on a shaker (Vortemp56, Labnet) at 300 rpm. All reactions were started by adding NADPH and stopped by adding 50 µl of methanol. Samples were centrifuged at 20,000 RCF for 10 min before being transferred to HPLC vials for injection.

### Iridoid Profiling Using HPLC–MS

Samples were analyzed using an Elute LC system (Bruker Daltonik, Bremen, Germany) equipped with an Acquity UPLC C18 column (2.1 × 50 mm, 1.7 µm, 100 Å; Waters) and coupled via electrospray ionization (ESI) to an Impact II q-TOF (Bruker Daltonik). Iridoids were separated at 40°C using a gradient from water (A) to acetonitrile (B), both modified with 0.1% formic acid, at a flow of 0.3 ml/min, redirecting to waste the first 60 s. The program starts with an isocratic flow of 99% A for 1 min, followed by an isocratic step of 95% A for 1.5 min, and then linearly decreases from 95% A in 2.5 min to 80% A in 6 min, then linearly decreases to 60% A in 8 min, and finally decreases to 0% A in 9 min. The column is then washed with 100% B for 2 min and equilibrated in 1% B for another 2 min before the next injection. Ionization was performed via pneumatic-assisted ESI in the negative mode, with a capillary voltage of 3.5 kV and a nebulizer pressure of 2.5 bar. Nitrogen gas was used as drying gas at 350°C and a flow of 11 l/min. The acquisition was set to 6 Hz, from 100 to 1000 m/z, with data-dependent fragmentation and active exclusion after 3 spectra, released after 0.2 min. Fragmentation was triggered on an absolute threshold of 400 and acquired on the most intense peaks with dynamic collision energy from 20 to 50 eV. At the beginning of each run, whereas the LC input was redirected to waste, a sodium formate–isopropanol solution was injected at 0.2 ml/h for the first 60 s of each run, and the m/z values were recalibrated using the expected cluster ion m/z values. Raw MS files were converted to mzXML using Bruker Data Analysis software (Bruker Daltonik, Bremen, Germany), and, when needed, extracted ion chromatograms (XICs) were exported to csv using MZMine2 v2.40.1 (Pluskal et al. 2010) and plotted using Excel.

### Iridoid Standards

Mussaenosidic acid and 8-*epi*-7-deoxyloganic acid were bought from AnalytiCon Discovery GmbH (Potsdam, Germany); scandoside and geniposidic acid were bought from Aobious Inc. (Gloucester, MA, USA); bartsioside were bought from Sigma-Aldrich/Merck KGaA (Darmstadt, Germany); and aucubin were bought from Extrasynthese (Genay, France). 7-Deoxyloganic acid and a mix of loganin epimers were synthesized as previously reported (Rodríguez-López et al. 2021).

### Data Analysis

Unless otherwise stated, data analysis was performed using R v4.0.3 (R Core Team 2020). Images were made using the *base* (R Core Team 2020), *ggplot2* (Wickman 2016), and *gplots* (Warnes et al. 2020) packages, and Venn diagrams were plotted using the *VennDiagram* library (Chen 2018). Phylogenetic tree analyses were performed using *phangorn* v2.6.3 (Schliep 2011) and *ape* v5.4-1 (Paradis and Schliep 2019) libraries, and dendrogram operations were facilitated with *dendextend* (Galili 2015). Nucleotide and amino acid sequences were handled using *seqinr* v4.2-5 (Charif and Lobry 2007), multiple alignments were performed using ClustalW (Larkin et al. 2007; Madeira et al. 2019), and trees were generated using Geneious Prime v2019.2.1 Tree Builder on a Jukes–Cantor genetic distance model and visualized using the interactive Tree of Life (Letunic and Bork 2019). Graph plots and geodesic calculations were done using the *igraph* package v1.2.6 (Csárdi and Nepusz 2006), handling of sparse adjacency matrices was facilitated by the *Matrix* library (Bates and Maechler 2019), and network metrics were obtained via *QuACN* library v1.8.0 (Mueller et al. 2011). Dimensionality reduction by Uniform Manifold Approximation and Projection (UMAP) was performed using the *uwot* package (Melville 2020), and by SOMs using the *kohonen* library (Wehrens and Kruisselbrink 2018). Parallel processing of data was facilitated by the *parallel* (R Core Team 2020) and *snow* (Tierney et al. 2018) libraries. Molecules were drawn using ChemDraw.

## Supplementary Material

msac057_Supplementary_Data

## Data Availability

The data that support the findings of this study are available as EDMOND collection in the repository link https://edmond.mpdl.mpg.de/imeji/collection/v0lZ4EROIHQYjrop. *Lamium album* raw sequence data are available via the NCBI Short Read Archive under BioProject PRJNA758356 (available upon publication). Aucubin Synthase sequences are available in GenBank under accession numbers OM256537 (*C. americana*), OM256538 (*V. agnus-castus*), and OM256539 (*P. tomentosa*).
