# Supplementary material for: Phylogeny-Aware Chemoinformatic Analysis of Chemical Diversity in Lamiaceae Enables Iridoid Pathway Assembly and Discovery of Aucubin Synthase
Source: Mol Biol Evol. 2022 Mar 17;39(4):msac057. doi: 10.1093/molbev/msac057 (PMC9048965; doi:10.1093/molbev/msac057)
Supplement: msac057_Supplementary_Data [file msac057_supplementary_data.zip › Supplementary_Material_MBE.pdf]

Supplementary Information for the article:

Phylogeny-aware chemoinformatic analysis of chemical diversity in the Lamiaceae enables iridoid pathway assembly and discovery of aucubin synthase.

Carlos E. Rodríguez-López[1*], Yindi Jiang[1], Mohamed O. Kamileen[1], Benjamin R. Lichman[2], Benke Hong[1], Brieanne Vaillancourt[3], C. Robin Buell[4], Sarah E. O'Connor[1]*.

[1] Department of Natural Product Biosynthesis, Max Planck Institute for Chemical Ecology, 07745 Jena, Germany

[2] Department of Biology, University of York, YO10 5DD York, United Kingdom

[3] Center for Applied Genetic Technologies, University of Georgia, Athens, GA 30602, USA

[4] Department of Crop & Soil Sciences, University of Georgia, Athens, GA 30602, USA

Corresponding Authors:

Sarah E. O'Connor: oconnor@ice.mpg.de

Carlos E. Rodríguez-López: clopez@ice.mpg.de

Apendices
Appendix S1. Assignation of values for codifying molecules into vectors
Appendix S2. Calculation of phylogenetic scores
Appendix S3. Initial estimation of sampling and model parameters for the Pathway Evolution Hypothesis
Appendix S4. Selection of network descriptors
Appendix S5. Evaluating the Pathway Reconstitution Algorithm

Supplementary Figures
Supplementary Figure S1. Simplified examples of the implementation.
Supplementary Figure S2. Choosing surrogates for evaluation.
Supplementary Figure S3. Mahalanobis distance distribution.
Supplementary Figure S4. Diagnostic ability of the model.
Supplementary Figure S5. Matthew's Correlation Coefficient of enzymes and metabolites.
Supplementary Figure S6. Pathway Hypothesis for Callicarpa spp. (Callicarpoideae.)
Supplementary Figure S7. Pathway Hypothesis for Westringia spp. (Prostantheroideae.)
Supplementary Figure S8. Pathway Hypothesis for Salvia spp. (Nepetoideae.)
Supplementary Figure S9. Pathway Hypothesis for Nepeta spp. (Nepetoideae.)
Supplementary Figure S10. Pathway Hypothesis for Vitex spp. (Viticoideae.)
Supplementary Figure S11. Pathway Hypothesis for Gmelina spp. (Premnoideae.)
Supplementary Figure S12. Pathway Hypothesis for Premna spp. (Premnoideae.)
Supplementary Figure S13. Pathway Hypothesis for Holmskioldia spp. (Scutellaroideae.)
Supplementary Figure S14. Pathway Hypothesis for Scutellaria spp. (Scutellaroideae.)
Supplementary Figure S15. Pathway Hypothesis for Rotheca spp. (Ajugoideae.)
Supplementary Figure S16. Pathway Hypothesis for Clerodendrum spp. (Ajugoideae.)
Supplementary Figure S17. Pathway Hypothesis for Teucrium spp. (Ajugoideae.)
Supplementary Figure S18. Pathway Hypothesis for Ajuga spp. (Ajugoideae.)
Supplementary Figure S19. Pathway Hypothesis for Betonica spp. (Lamioideae.)
Supplementary Figure S20. Pathway Hypothesis for Phlomis spp. (Lamioideae.)
Supplementary Figure S21. Pathway Hypothesis for Leonurus spp. (Lamioideae.)
Supplementary Figure S22. Pathway Hypothesis for Leonotis spp. (Lamioideae.)
Supplementary Figure S23. Pathway Hypothesis for Ballota spp. (Lamioideae.)
Supplementary Figure S24. Pathway Hypothesis for Lamium spp. (Lamioideae.)
Supplementary Figure S25. Graphical representation of phylogenetic weights.
Supplementary Figure S26. Candidate gene selection.
Supplementary Figure S27. Protein alignment tree of Orthogroup OG0001031.
Supplementary Figure S28. Graphical representation of the difference vectors in two carbons.
Supplementary Figure S29. Graphical representation of reaction vectors.
Supplementary Figure S30. Distance calculations for two sample reactions.
Supplementary Figure S31. Parameter estimation.
Supplementary Figure S32. Selection of Network Descriptors.

Supplementary Figure S33. Principal Component Analysis of Selected Network Descriptors.
Supplementary Figure S34. Evaluating the model.

Supplementary Tables

Supplementary Table S1. Network Descriptors by category, along with their abbreviation.

Algorithms

Algorithm S1. Pseudocode Algorithm of the naïve model
Algorithm S2. Pseudocode Algorithm of the phylogenetic pruning
Algorithm S3. Pseudocode Algorithm of the Pathway Evolution Model
Algorithm S4. Simplified algorithm generating an adjacency matrix from codified molecules

Appendix S1. Assignation of values for codifying molecules into vectors

The concept of representing a molecule as a coordinate in a definite space, following ordinal, independent oxidations in each carbon, presents one major challenge: the presence of double bonds, as they necessarily involve two carbons. Although double bonds can arise directly through the action of C-C oxidoreductases, we reasonably assume that in iridoids, the activation of saturated carbons is through monooxygenases, such as Cytochrome p450 enzymes. Thus, all C-C double bonds in the model arise from either dehydration or keto-enol tautomerism, further challenging the representation as ordinal oxidations. We decided to generalize the representation of molecules to the complex plane, where double bonds are placed in the imaginary axis.

In this representation, we can use a generalized version of the Manhattan distance, where the distance is calculated as the sum of the magnitudes of the vectorial difference in each carbon, as shown in **Eq. S1**:

$$d(p,q) = \sum_{j=1}^{n} \left\| p_j - q_j \right\|$$

$$d(p,q) = \sum_{j=1}^{n} \left| \left\{ \left[\mathrm{Re}(p_j) - \mathrm{Re}(q_j)\right]^2 + \left[\mathrm{Im}(p_j) - \mathrm{Im}(q_j)\right]^2 \right\}^{1/2} \right| \qquad \textbf{(Eq. S1)}$$

Where the distance between points *p* and *q* is the sum of the magnitude of their differences. If the difference involves only real numbers (i.e. sequential oxidations) this is exactly the same as the Manhattan distance (**Eq. S2**).

$$d(p,q) = \sum_{j=1}^{n} \left| \left\{ \left[\mathrm{Re}(p_j) - \mathrm{Re}(q_j)\right]^2 \right\}^{1/2} \right|$$

$$d(p,q) = \sum_{j=1}^{n} \left| p_j - q_j \right| \qquad \textbf{(Eq. S2)}$$

Now, we need to choose the values of the modifications in the imaginary axis that will give a useful connection when **Eq. S1** is applied. This is a molecule with two carbons, one with an alcohol, should connect to a molecule with a double bond in both carbons (**Supplementary figure S28 - blue**), while avoiding connecting a molecule with two completely saturated carbons to a double bond carbon (**Supplementary figure S28 - red**), and other spurious connections. Similarly, a molecule with a double bond should be able to connect to a molecule with an epoxide (**Supplementary figure S28 - green**).

As it is evident from **Supplementary figure S28**, while a value of β can easily be chosen to give a distance of one using **Eq. S1** (β=0.5), due to the triangle inequality no values of γ and α exist that will do so. We chose a value of γ=0 to simplify calculations. This also has the advantage of keeping a relation to oxidation, as the carbon with the alcohol is reduced, being closer to the origin, while the saturated carbon is oxidized, gaining distance from the origin.

Now, while no value of α can give a distance of one, we can choose a value that can give an approximate difference of 1. Thus, if we choose convergent rounding as the approximation function, and we input the blue and red difference vectors into **Eq. S1**, we get that:

$$d_{blue} = |\alpha| + \{1 + \alpha^2\}^{1/2} < 1.5$$

$$d_{red} = 2|\alpha| \leq 0.5$$

We chose α=1/4, which means that all molecules with a rounded, generalized Manhattan difference of 1 are one reaction apart. While this function loses the properties of topological distance, (e.g. the difference between C-C and C=C is approximated to zero,) by exclusively connecting molecules with differences of 1 in a graph, geodesic distance is related to biosynthetic distance.

Two spurious relations arise, however: one inherent to the Manhattan generalization and the other to the rounding estimation. Since the generalized Manhattan difference calculates the magnitude, but not the direction of the vector difference, an implausible superoxidation (**Supplementary figure S29, red**) has the same magnitude as a dehydration (or hydration) reaction (**Supplementary figure S29, blue**). This is easily solved programmatically, as in this cases the difference $p_j - q_j$ has a *tan(φ)*=¼, and thus can be removed with a conditional. The other spurious relation arises from four double bonds having a sum of 1, which is solved programmatically by only keeping reactions in which the imaginary part is affected in either 0 or 2 carbons. A step by step calculation for two reactions, including a keto-enol tautomerism and a 1,4 reduction, is shown in **Supplementary figure S30**.

The algorithm yielding the adjacency matrix for the graph connecting vector-encoded molecules is shown in **Algorithm S4**. Once the algorithm is defined, we can calculate the graph of the chemical space containing all possible iridoid scaffolds, within the assumptions made. For that, we calculate through brute force all possible combinations of values for all Carbons except those in positions 1 and 9, which are not modified in the reported molecules. Then we remove molecule vectors with uneven number of imaginary numbers, and that violate the carbon valence. This process yields a set of 62,384 compounds, to which we applied **Algorithm S4** (see **Supplementary File 2**), producing a graph with 821,876 edges, corresponding to reactions (**Supplementary File 3**). This graph is the representation of the iridoids chemical space.

Appendix S2. Calculation of phylogenetic scores

The phylogenetic correlation matrix C was calculated by **Eq. S3:**

$$C = [\mathrm{diag}(V)]^{-1/2} V [\mathrm{diag}(V)]^{-1/2} \qquad \textbf{(Eq.S3)}$$

Where the covariance matrix V refers to the expected covariance under a random Brownian motion model (Martins and Hansen 1997; Garland and Ives 2000), estimated by **Eq. S4**:

$$V = var[\epsilon_S]_{ij} = \gamma t_r \qquad \textbf{(Eq.S4)}$$

The value of V at position $[i,j]$ ($var[\epsilon_S]_{ij}$) is estimated as the distance (t_r) in the phylogeny between the root and the common ancestor of species i and j, multiplied by a constant γ (Martins and Hansen 1997; Garland and Ives 2000), which we consider as 1 for this calculation. Thus, it assigns a higher value to molecules present in the closest species, which assumes that if the molecule is reported in a close genus, it is likely to be also in the current one (see **Supplementary figure S25**). Felsenstein's weights at the root, (Felsenstein 1985b, a), also called phylogenetic center of mass, was calculated using **Eq. S5:**

$$F_w = \frac{\sum_i V^{-1}}{\sum_{i,j} V^{-1}} \qquad \textbf{(Eq.S5)}$$

Where the weights at the root are calculated as the row sums of the solved covariance matrix (as calculated using Eq. 2), scaled by its total values. These weights assign a higher value to molecules with a higher chance of being at the ancestral pathways (see **Supplementary figure S25b**). As it can be seen in **Supplementary figure S25**, ***C*** is a square matrix, and weights of each molecule will be species specific and depend on how close the reported species is. On the other hand, $\boldsymbol{F_w}$ is a vector, and the weight of the reports will be higher the closest the species is to the common ancestor, regardless of the distance to the species which is being evaluated. Thus, the approaches should offer complementary values, with either higher weights at the tips (***C***) or higher weight at the roots ($\boldsymbol{F_w}$) of the tree.

Appendix S3. Initial estimation of sampling and model parameters for the Pathway Evolution Hypothesis.

To estimate the percentage of molecules sampled, we applied the naïve *Pathway Reconstitution Algorithm* (**Algorithm S1** with n = 0) to the *in natura* dataset, and for each species we compared the number of measured metabolites with the number of predicted metabolites, which estimates the upper limit of sampling. As it can be seen in **Supplementary figure S31a**, we can estimate that the upper limit for most species sampling as being between 10 and 60%; thus, we sampled the multiverse reports by increments of 10% until reaching 60%. To approximate the size of the ancestral pathway, we applied Felsenstein weights at the root to the Pathway Reconstruction Algorithm predictions, to calculate the probability of each molecule to be in the ancestral pathway. Following a strict cutoff of scores > 0.9, we estimate 13 metabolites in the root, and with a relaxed cutoff of 0.5, we estimate 20 metabolites at the root (**Supplementary figure S31b**). Since these are lower limit estimations, we decided to explore from 10 to 40 reactions at the root, in increments of 10. We cannot estimate the maximum number of enzymes the pathway can hold, so we decided to explore thresholds of 25, 50 and 75, removing conflicting combinations (i.e. 30 and 40 reactions at root with a threshold of 25 maximum enzymes). Similarly, we cannot estimate the rounds of innovation per phylogenetic unit of distance, so we explored a wide window from 10^6 divided by powers of 2 from 0 to 9, giving a range from 1.9×10^3 to 1×10^6 novel enzyme activities in the Iridoid chemical space per unit of phylogenetic distance.

Appendix S4. Selection of network descriptors

The main objective of the *Pathway Evolution Hypothesis* is to output surrogate pathways that have a similar network structure as the real biosynthetic pathways. Since comparing network-to-network results virtually impossible given the number of networks generated, we decided to use QuACN (Mueller, et al. 2011) to calculate topological network descriptors. Since this is a novel case, for which we did not know which topological descriptors would better describe the data, we decided to calculate all the computable descriptors in four relevant QuACN categories: descriptors based on distances in a graph (1xxx), based on graph-invariants (2xxx), classical entropy-based descriptors (3xxx) and recent graph complexity measures (4xxx) (Mueller, et al. 2011). To minimize redundancy, which would affect downstream calculations, we calculated the spearman correlation of descriptors in all predictions with all samples, and used Ward's algorithm of hierarchical clustering to group metrics with highly similar results, using a height of 0.05 as a cutoff (**Supplementary figure S32a**). This greatly reduced from 55 to 16 representative graph metrics (**Supplementary figure S32b)** by removing highly redundant measures (**Supplementary Table S1**.)

It is important to note that these were selected by the correlation in the sub-sampled graphs, subject to the naïve Pathway Reconstruction Algorithm. Naturally, the network structures of the predictions are not similar to their parent "real" pathways even with a 100% sampling rate, as evidenced by their PCA (**Supplementary figure S33a**). This is mostly due to predictions having (expectedly) more interconnections, being calculated with the naïve Pathway Reconstruction Algorithm, resulting in higher medium articulation (MA), complexity (CIB), and spanning tree sensitivity (STS, STSD) metrics (**Supplementary figure S33b**). This is the reason we use a

comparison between estimates of *in silico* and *in natura* pathways, because the real *in natura* pathways are unknown, making a comparison with the parent *in silico* graphs impossible.

Appendix S5. Evaluating the Pathway Reconstitution Algorithm

To evaluate how well the Pathway Reconstruction Algorithm works, we need to clearly define what constitutes a False Positive, a False Negative, a True Positive and a True Negative. The general process is shown in **Supplementary figure S34**. We have a pathway for a given species, that we know is true (**Supplementary figure S34a**), and we sampled it (**Supplementary figure S34b**), and reconstructed it using the Pathway Reconstruction Algorithm (**Supplementary figure S34c**). We want to evaluate the predictions, so we do not consider the measured metabolites for any calculation, only the predicted nodes (**Supplementary figure S34c** – white/gray). By extension, the true nodes of the pathway that are not within the origin of the pathway and the measured compounds are also not considered for the calculation, giving preference to conservative models. If a predicted node (or edge) is in the pathway, but not in the prediction, it is considered a **False Negative** (**Supplementary figure S34d** – red), and if it is in the model and the pathway, it is considered a **True Positive** (**Supplementary figure S34d** – cyan). Similarly, if a predicted node (or edge) is not in the pathway, but it is in the model, it is considered a **False Positive** (**Supplementary figure S34d** – yellow). To determine the true negatives, we calculate all the possible minimal routes between each measured compound and the origin (**Supplementary figure S34**– black node) using the Iridoids chemical space, and we considered the nodes (or edges) as **True Negatives** (**Supplementary figure S34d** – green) if they were not in the model. It is important to note that this excludes the chemical space that is not part of the shortest routes between the origin and the sampled compounds; otherwise, the number of true negatives would be inflated.

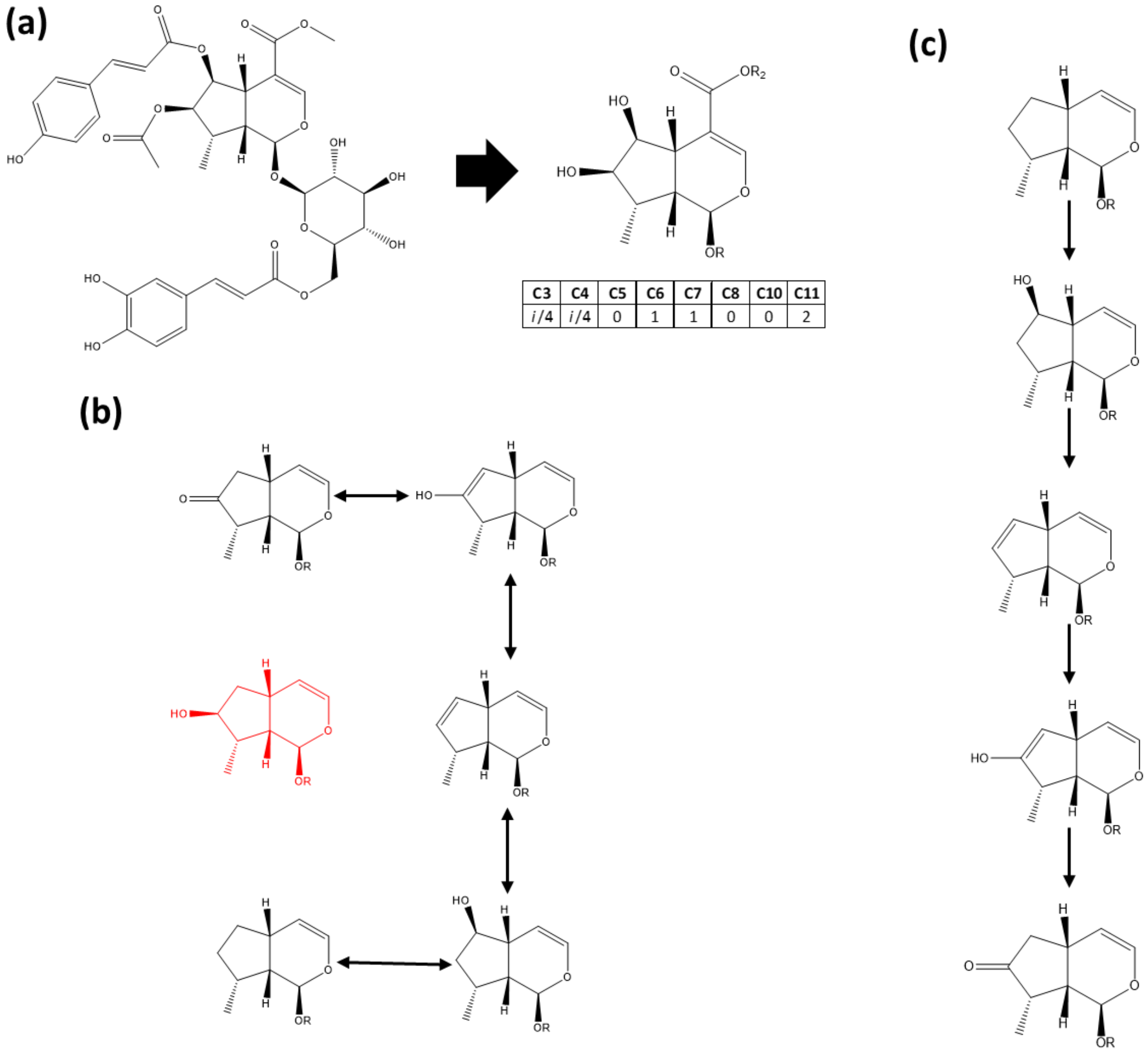

C3	C4	C5	C6	C7	C8	C10	C11
i/4	*i*/4	0	1	1	0	0	2

Supplementary Figure S1. Simplified examples of the implementation. (a) Shows the process of converting the report of a decorated iridoid glucoside (left) to its scaffold (right) and to the coordinate system. Note that no assumption is made on the esters in C11 or the glucoside status in C1. (b) Exemplifies why taking the shortest geodesic distance is not equivalent to the minimum number of reactions (which is of two reactions, via the non-reported red intermediary), but to the minimal number of reactions connecting all reports (four). The result of this is shown in the sample pathway in (c).

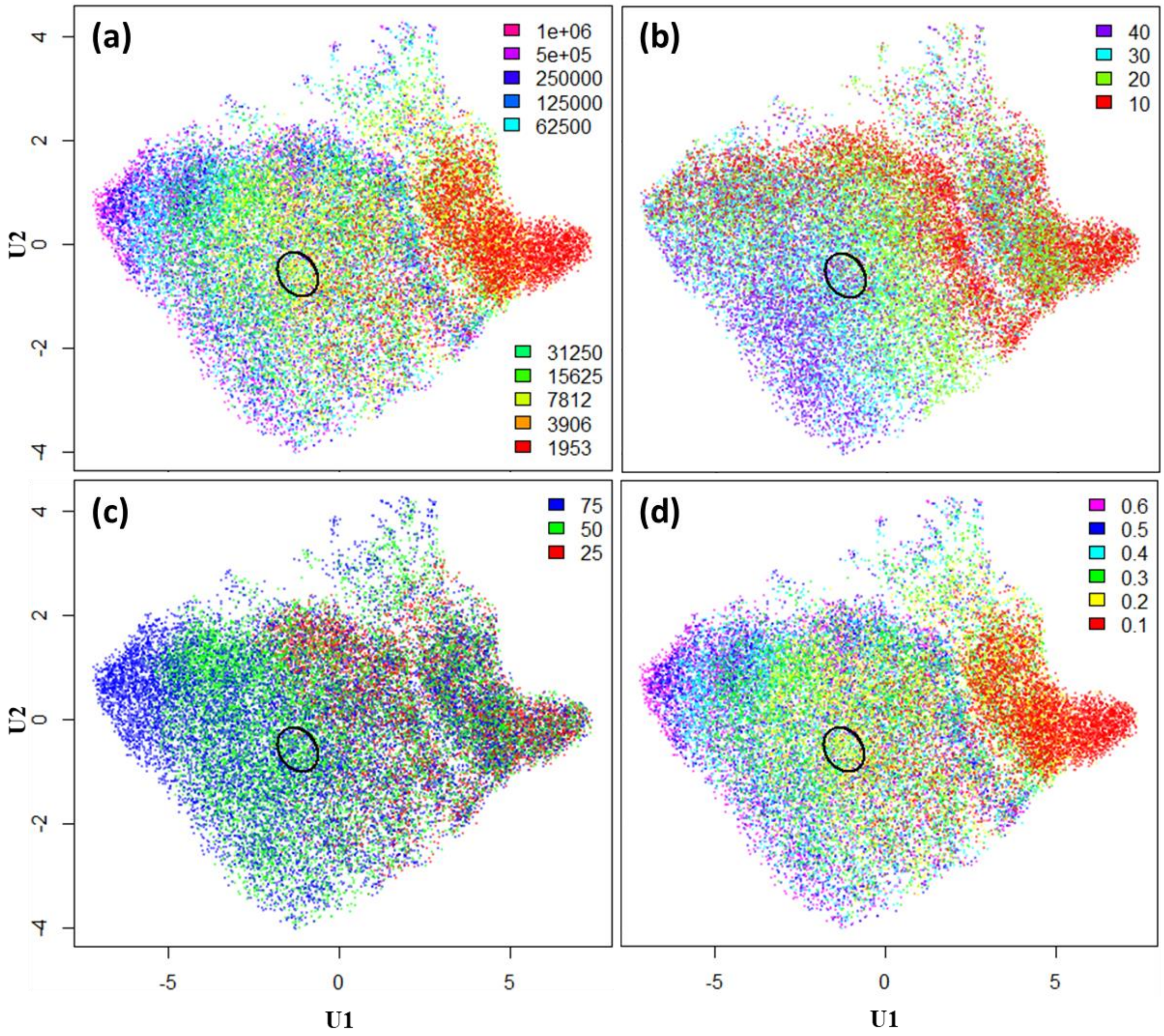

Supplementary Figure S2. Choosing surrogates for evaluation. The figure shows the results of an UMAP projection of a subset of 16 network descriptors for the 21 genus considered, after $\log_{10}$ transformation and z-score scaling. Each point represents a set of 21 networks, one for each genus, generated by **Algorithm 3** with a different combination of parameters. The same projection is shown four times with different coloring depending on (a) number of rounds of exploration per unit of distance in the phylogenetic tree, (b) number of reactions of the ancestral pathway, (c) the maximum number of enzymes allowed, and (d) sampling percentage. The sets of pathways whose projection falls within the Mahalanobis distance from the projection of the *in natura* naïve model equivalent to a $\chi_2^2 < 0.99$ (black ellipse) were chosen as surrogates.

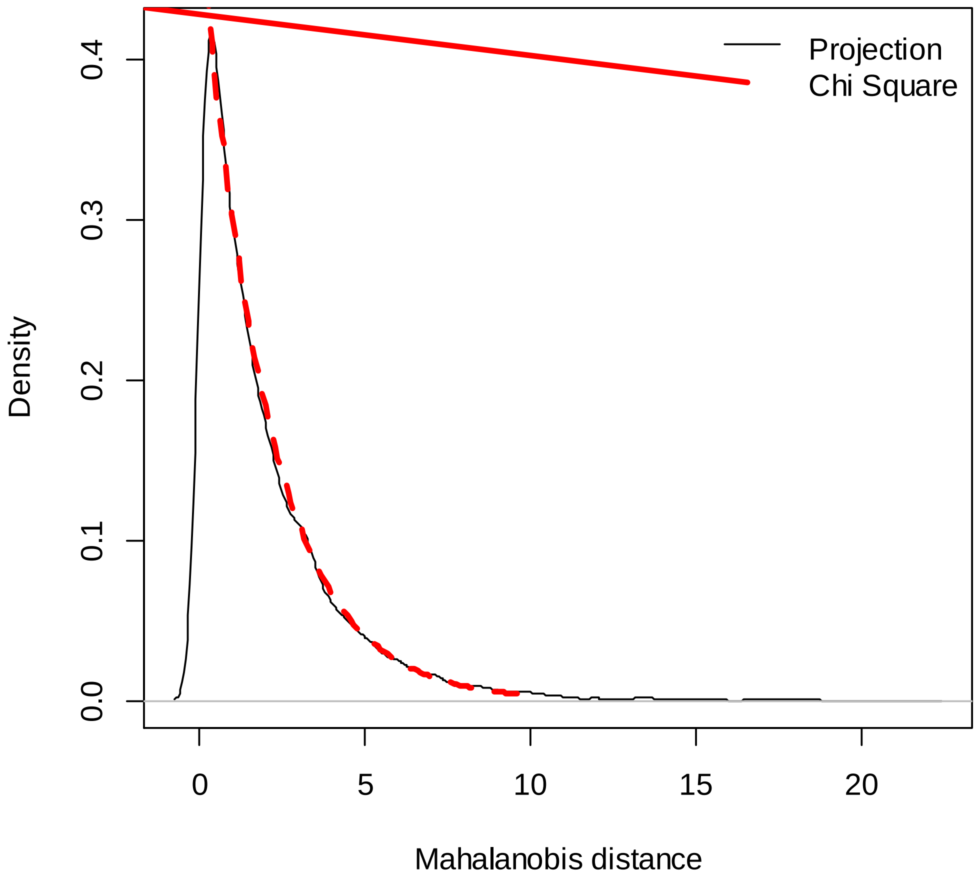

Supplementary Figure S3. Mahalanobis distance distribution. In black, the Mahalanobis distances of 10,000 UMAP projections of *in natura* iridoids naïve prediction, and in red, a Chi-square distribution with 2 degrees of freedom (χ_2^2). It can be clearly seen that the distributions overlap. We chose as surrogates models with projections having a $\chi_2^2 < 0.99$.

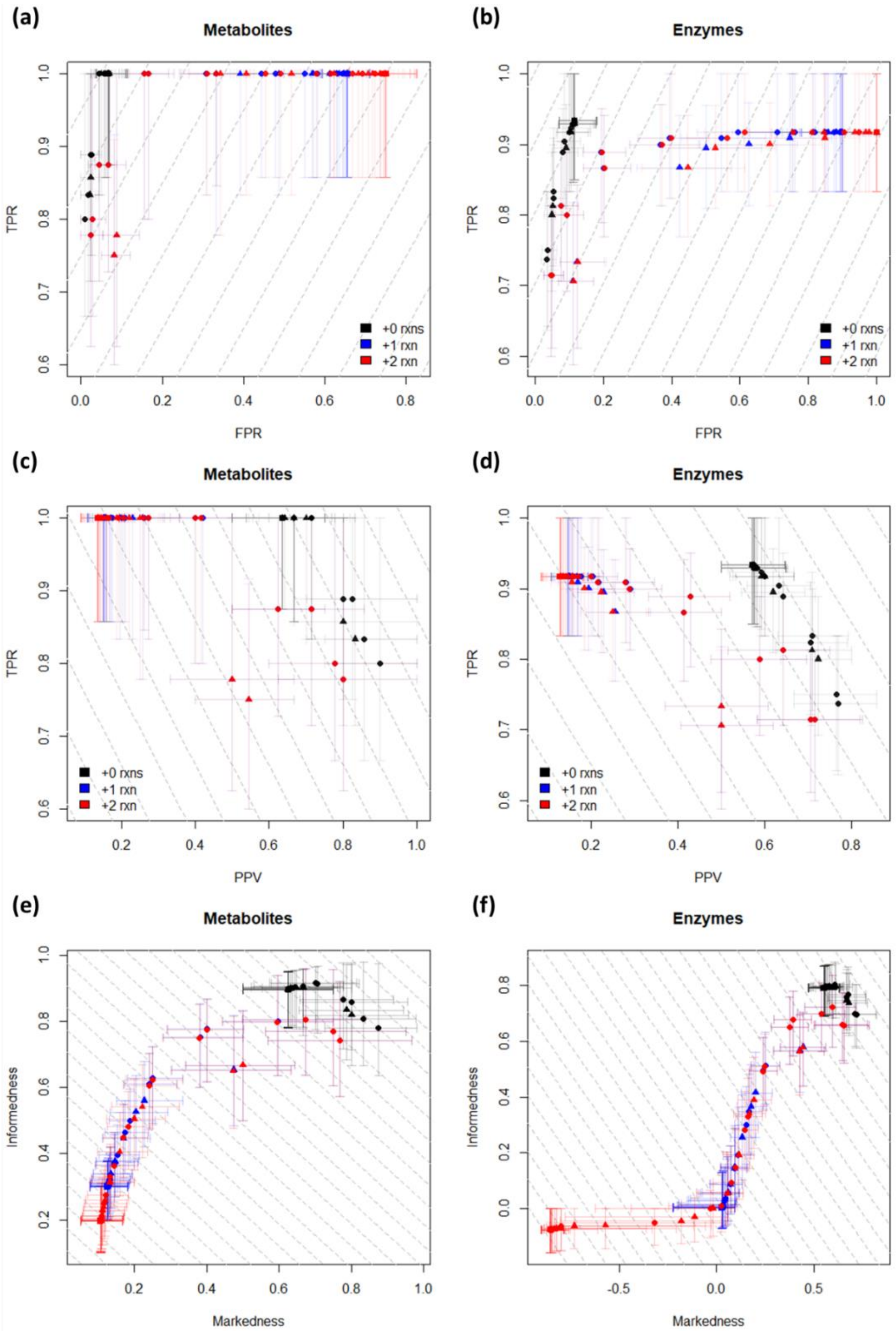

Supplementary Figure S4. Diagnostic ability of the model. Plots of True Positive Rate (TPR, recall) against False Positive Rate (FPR), also known as ROC curve, are shown for metabolite (a) and enzyme (b) predictions. Similarly, plots of Positive Predictive Value (PPV, precision) against TPR, display the precision-recall trade-off of the metabolite (c) and enzyme (d) predictions. Finally, plots of bookmaker informedness against markedness, components of Matthew's Correlation Coefficient, for metabolites (e) and enzymes (f). In black, models with no reactions added; in blue, reports were extended by one reaction, and in red, by two. The points show the median, and the bars show the interquartile range (25th and 75th percentile), and isocost diagonals in dotted lines.

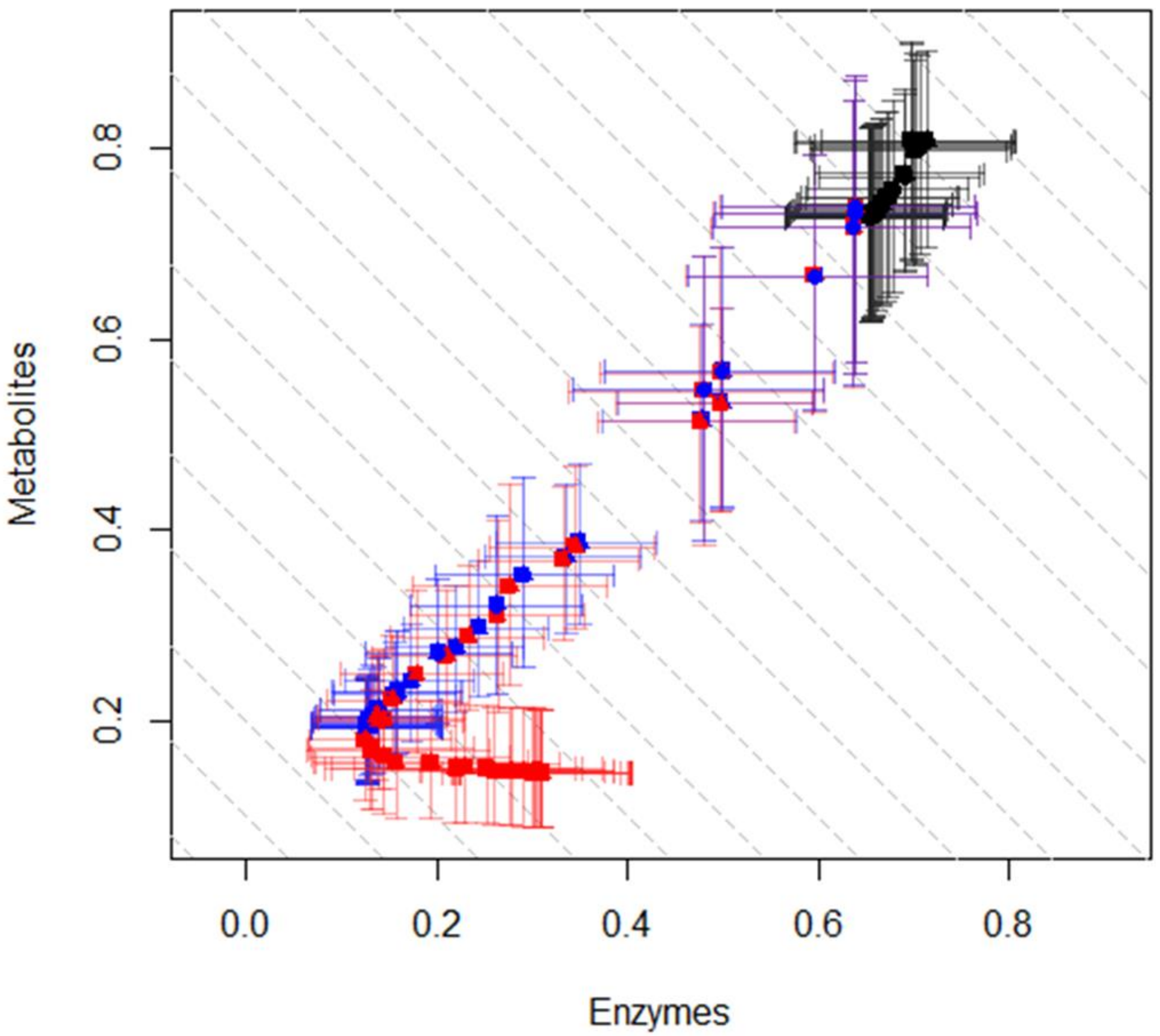

Supplementary Figure S5. Matthew's Correlation Coefficient of enzymes and metabolites. In black, models with no reactions added; in blue, reports were extended by one reaction, and in red, by two. The points show the median, and the bars show the interquartile range (25th and 75th percentile), and isocost diagonals are shown in dotted lines.

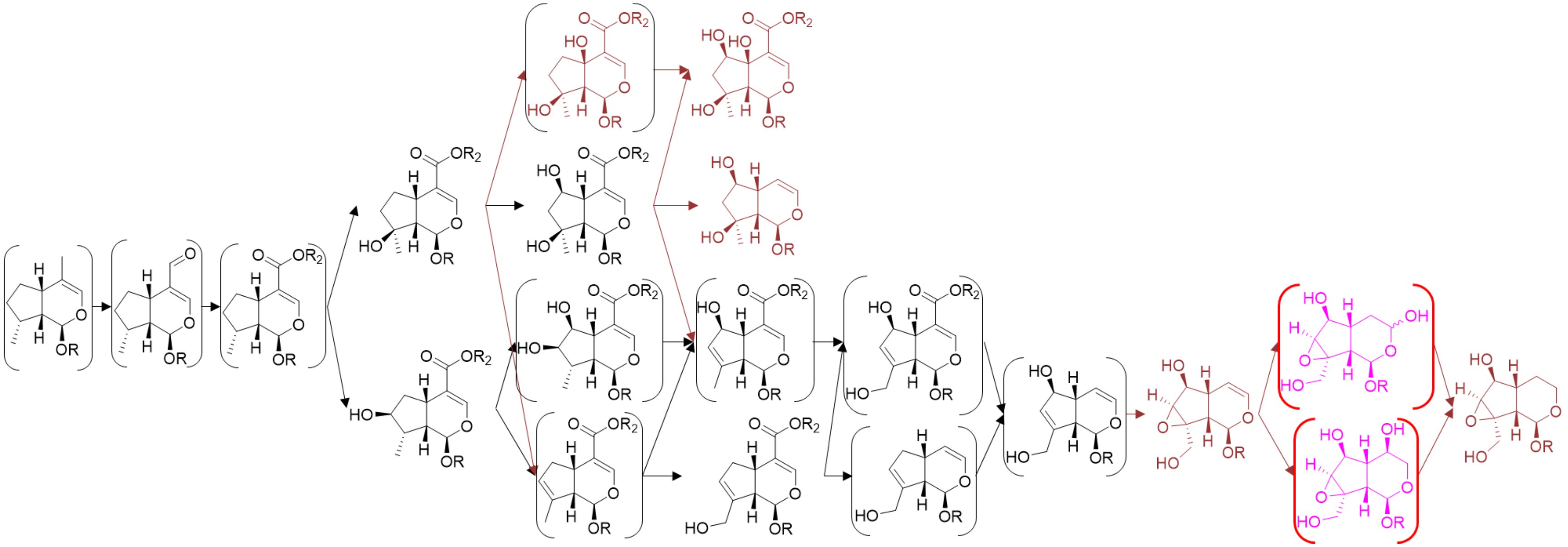

Supplementary Figure S6. Pathway Hypothesis for Callicarpa spp. (Callicarpoideae.) The metabolites and reactions expected to be present in the ancestral pathway are shown in black; metabolites reported in this genus, but not expected to be ancestral, are shown in Crail Red; and completely theoretical metabolites are shown in pink. Metabolites predicted by our model, but that are not reported in the genus, are shown brackets.

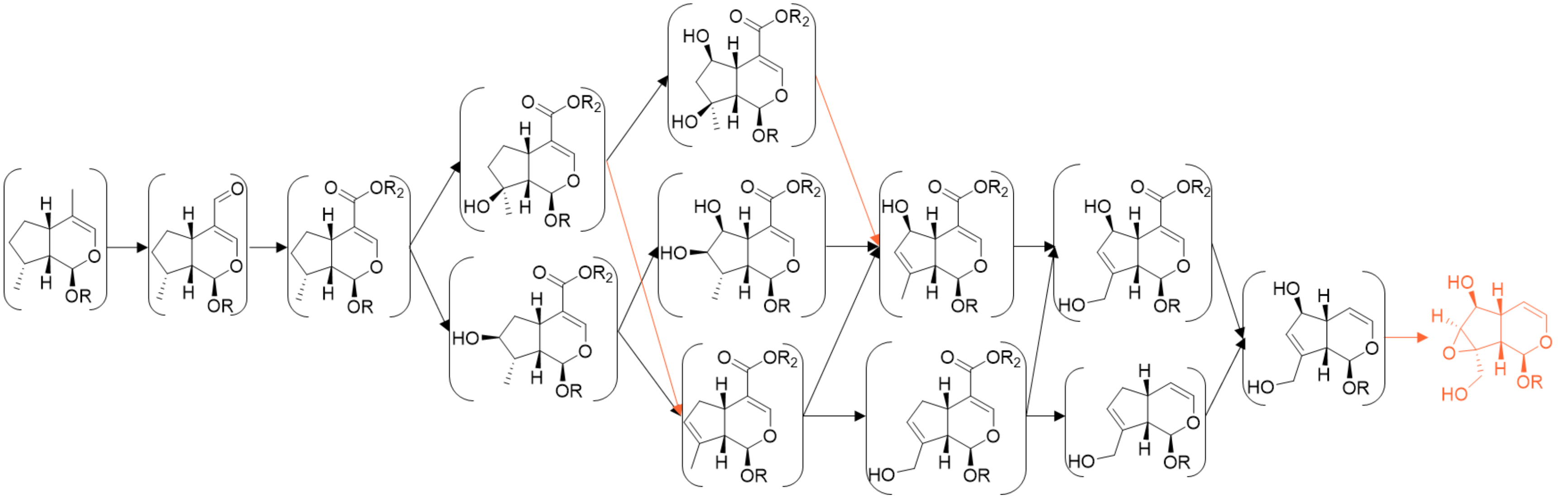

Supplementary Figure S7. Pathway Hypothesis for Westringia spp. (Prostantheroideae.) The metabolites and reactions expected to be present in the ancestral pathway are shown in black, and metabolites reported for this genus, and not expected to be ancestral, are shown in Vermilion. Metabolites predicted by our model, but that are not reported in the genus, are shown brackets.

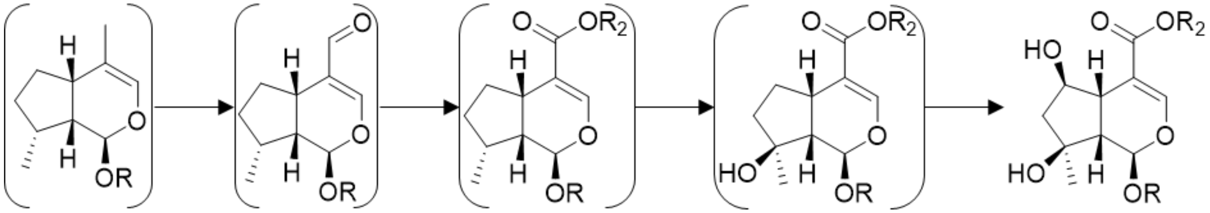

Supplementary Figure S8. Pathway Hypothesis for Salvia spp. (Nepetoideae.) Only metabolites expected in the ancestral pathway are reported in this genus. Metabolites predicted by our model, but that are not reported in the genus, are shown brackets.

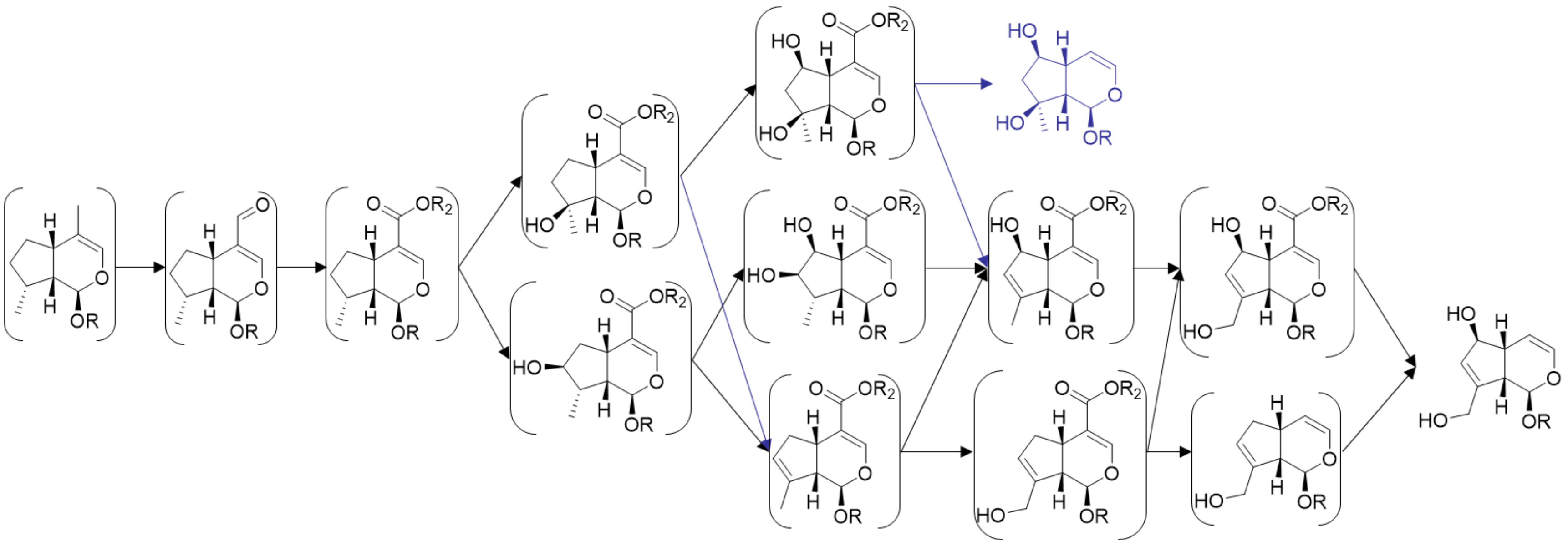

Supplementary Figure S9. Pathway Hypothesis for Nepeta spp. (Nepetoideae.) The metabolites and reactions expected to be present in the ancestral pathway are shown in black; metabolites reported in this genus, but not expected to be ancestral, are shown in Sapphire Blue. Metabolites predicted by our model, but that are not reported in the genus, are shown brackets.

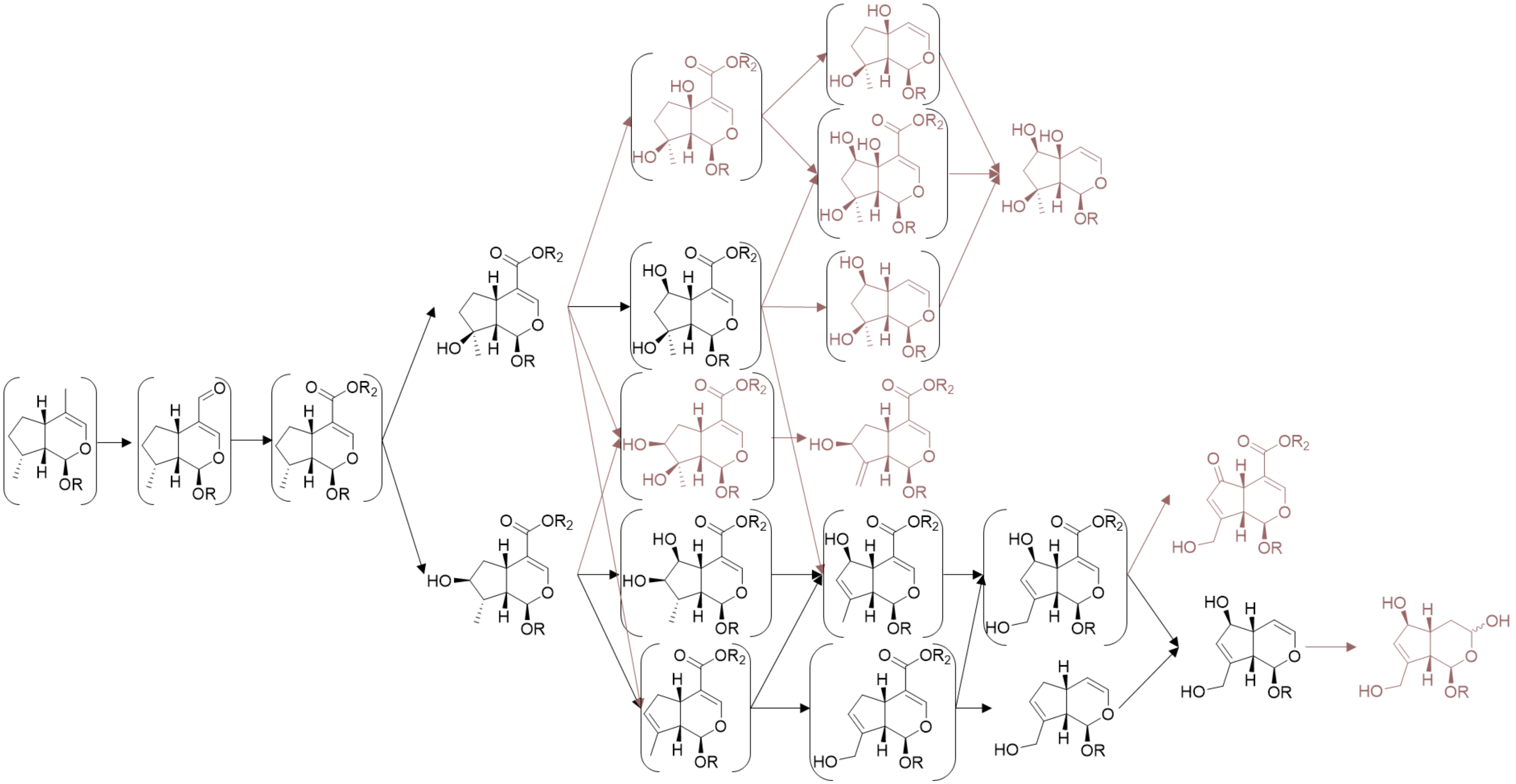

Supplementary Figure S10. Pathway Hypothesis for Vitex spp. (Viticoideae.) The metabolites and reactions expected to be present in the ancestral pathway are shown in black; metabolites reported in this genus, but not expected to be ancestral, are shown in Copper Rose. Metabolites predicted by our model, but that are not reported in the genus, are shown brackets.

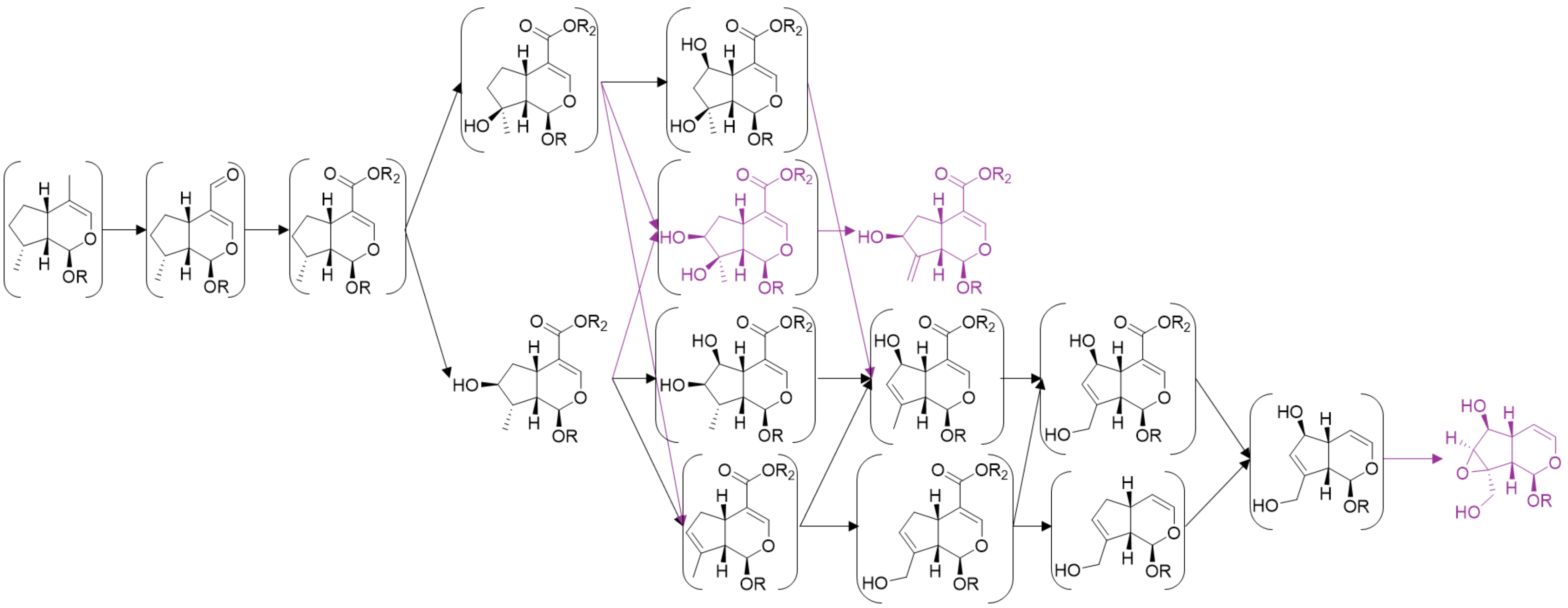

Supplementary Figure S11. Pathway Hypothesis for Gmelina spp. (Premnoideae). The metabolites and reactions expected to be present in the ancestral pathway are shown in black; metabolites reported in this genus, but not expected to be ancestral, are shown in Plum Violet. Metabolites predicted by our model, but that are not reported in the genus, are shown brackets.

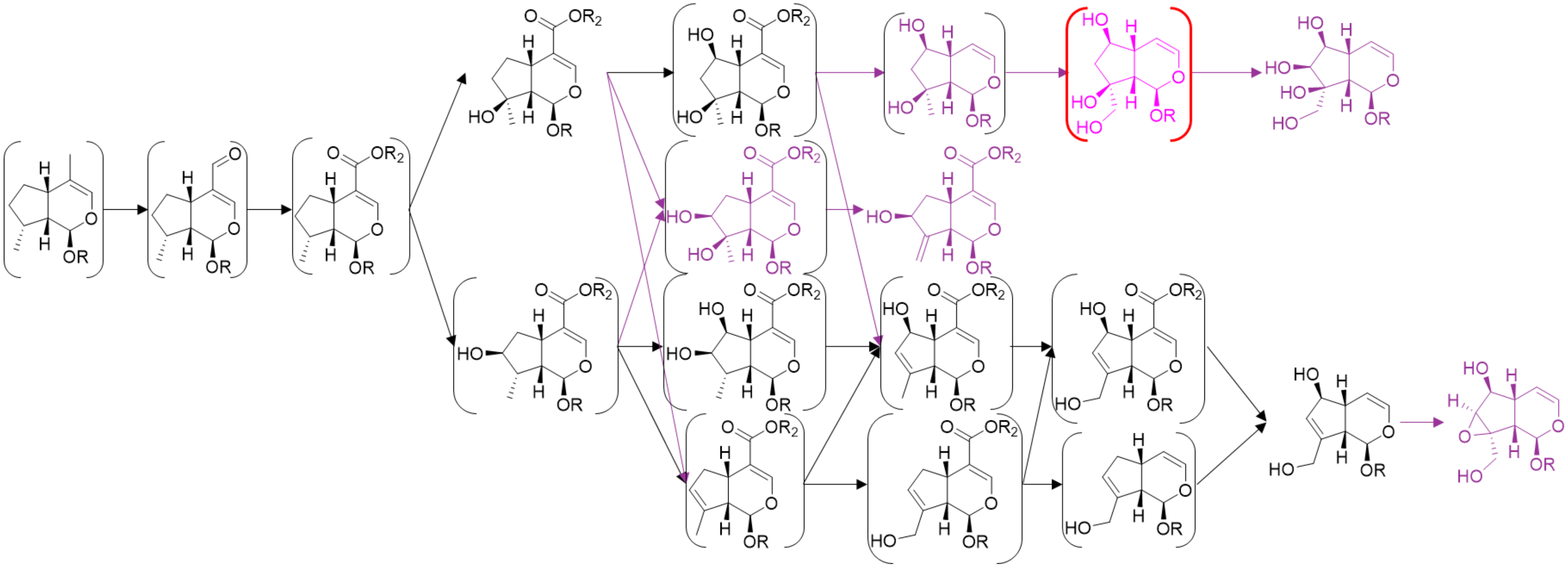

Supplementary Figure S12. Pathway Hypothesis for Premna spp. (Premnoideae). The metabolites and reactions expected to be present in the ancestral pathway are shown in black; metabolites reported in this genus, but not expected to be ancestral, are shown in Plum Violet; and completely theoretical metabolites are shown in pink. Metabolites predicted by our model, but that are not reported in the genus, are shown brackets.

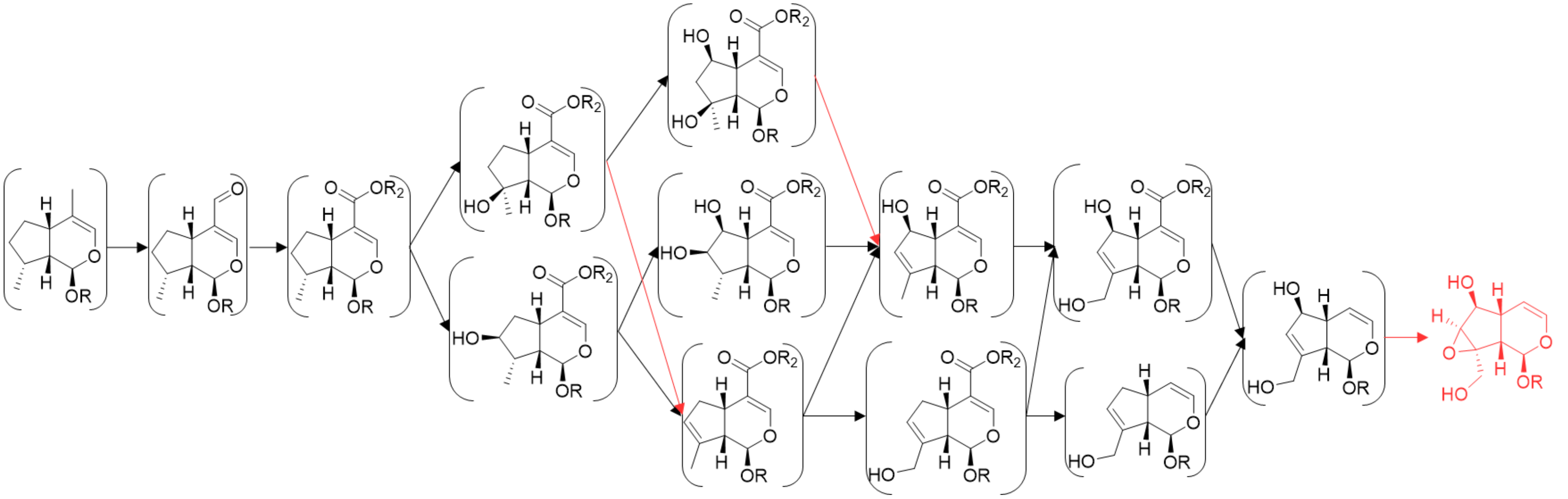

Supplementary Figure S13. Pathway Hypothesis for Holmskioldia spp. (Scutellaroideae). The metabolites and reactions expected to be present in the ancestral pathway are shown in black; metabolites reported in this genus, but not expected to be ancestral, are shown in Pomegranate Red. Metabolites predicted by our model, but that are not reported in the genus, are shown brackets.

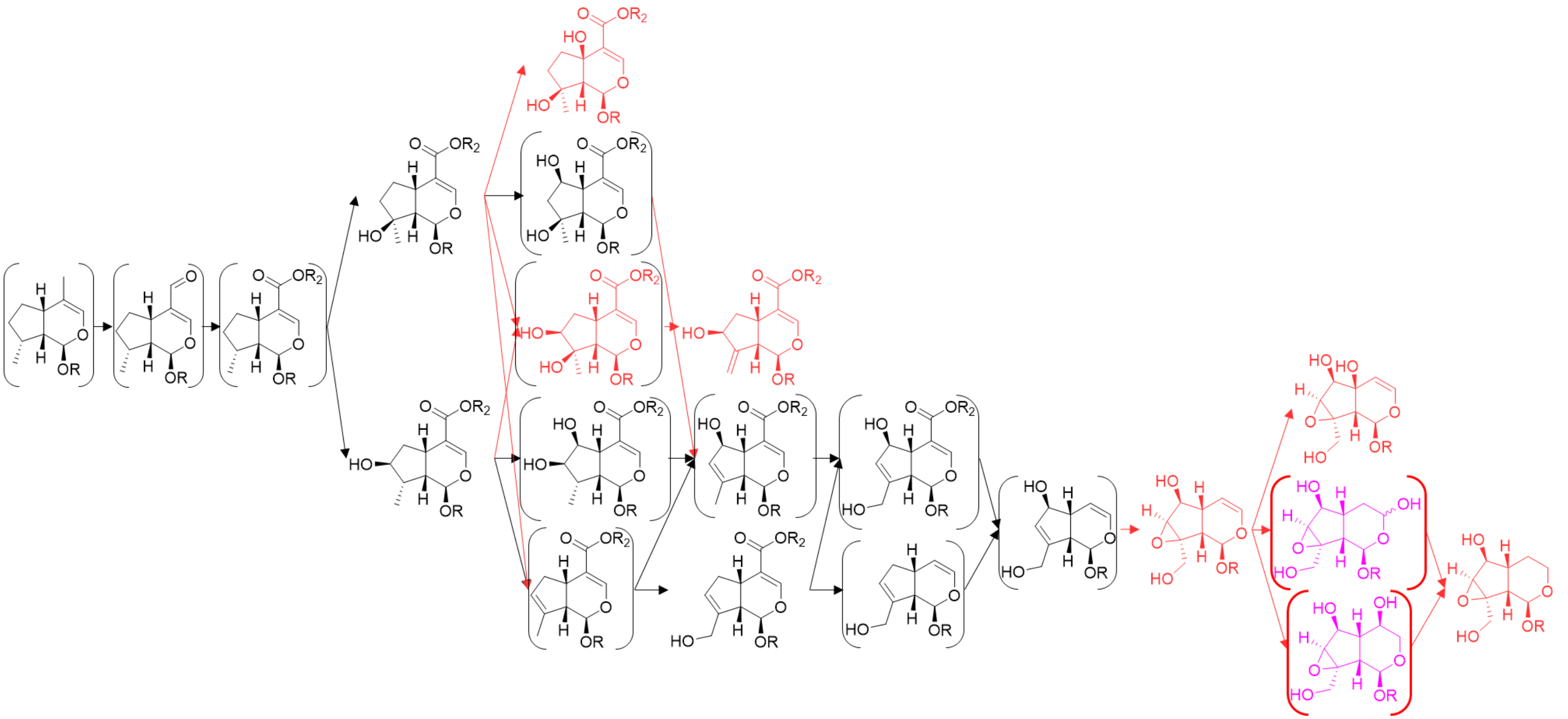

Supplementary Figure S14. Pathway Hypothesis for Scutellaria spp. (Scutellaroideae). The metabolites and reactions expected to be present in the ancestral pathway are shown in black; metabolites reported in this genus, but not expected to be ancestral, are shown in Pomegranate Red; and completely theoretical metabolites are shown in pink. Metabolites predicted by our model, but that are not reported in the genus, are shown brackets.

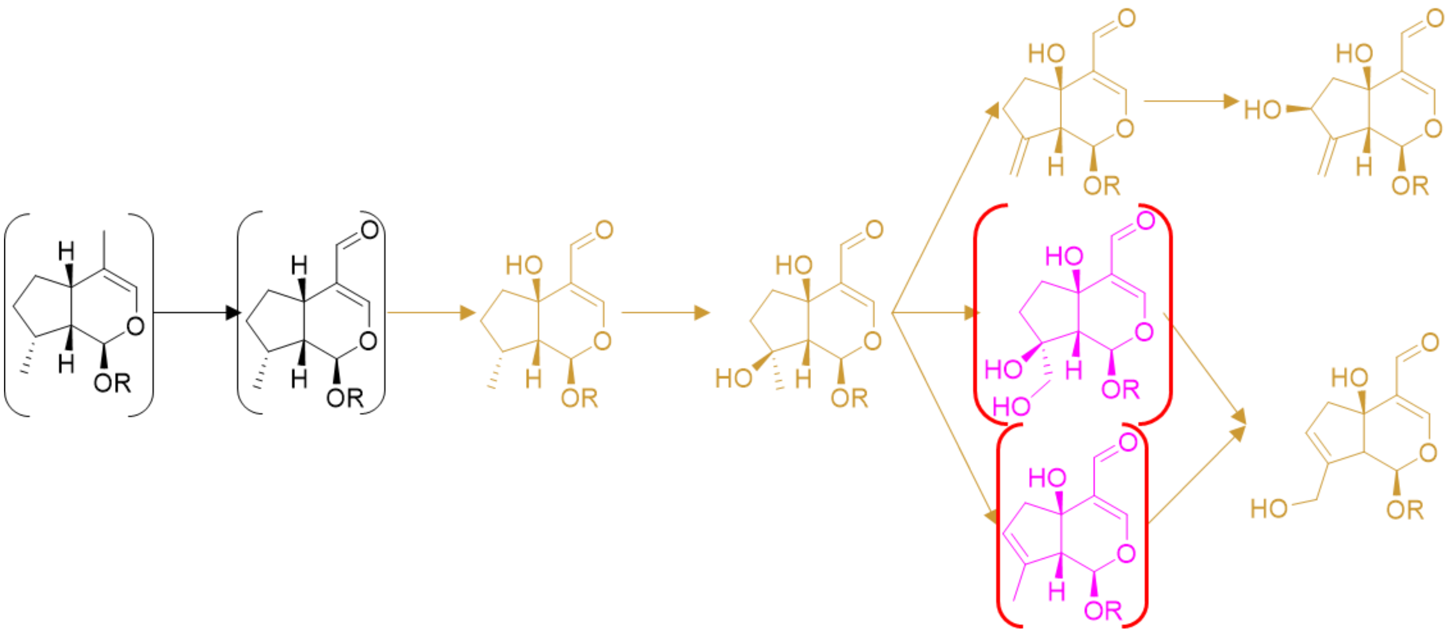

Supplementary Figure S15. Pathway Hypothesis for Rotheca spp. (Ajugoideae). The metabolites and reactions expected to be present in the ancestral pathway are shown in black; metabolites reported in this genus, but not expected to be ancestral, are shown in Tussock Yellow; and completely theoretical metabolites are shown in pink. Metabolites predicted by our model, but that are not reported in the genus, are shown brackets.

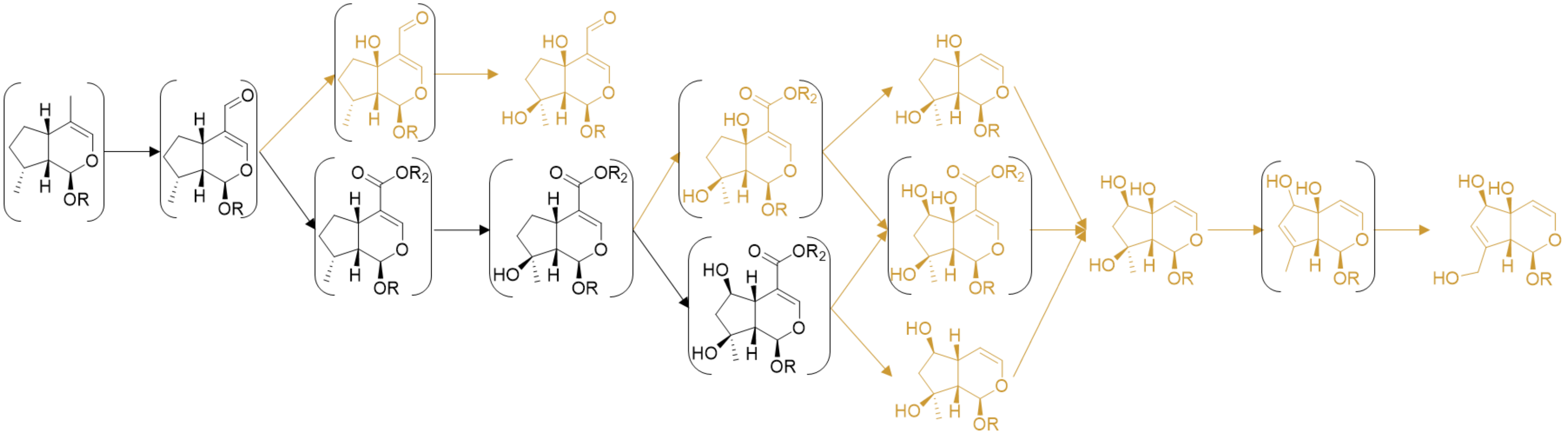

Supplementary Figure S16. Pathway Hypothesis for Clerodendrum spp. (Ajugoideae). The metabolites and reactions expected to be present in the ancestral pathway are shown in black; metabolites reported in this genus, but not expected to be ancestral, are shown in Tussock Yellow. Metabolites predicted by our model, but that are not reported in the genus, are shown brackets.

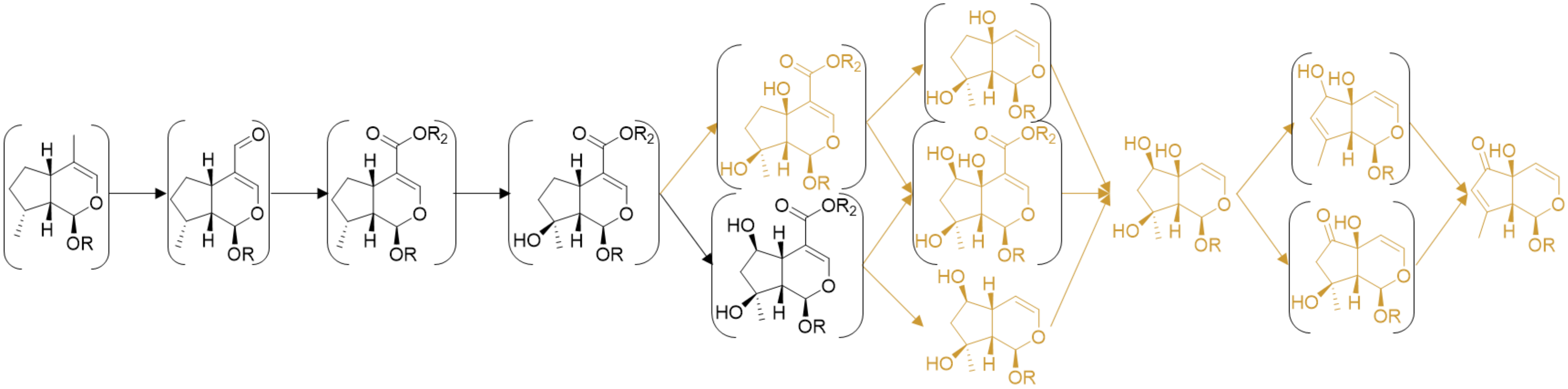

Supplementary Figure S17. Pathway Hypothesis for Teucrium spp. (Ajugoideae). The metabolites and reactions expected to be present in the ancestral pathway are shown in black; metabolites reported in this genus, but not expected to be ancestral, are shown in Tussock Yellow. Metabolites predicted by our model, but that are not reported in the genus, are shown brackets.

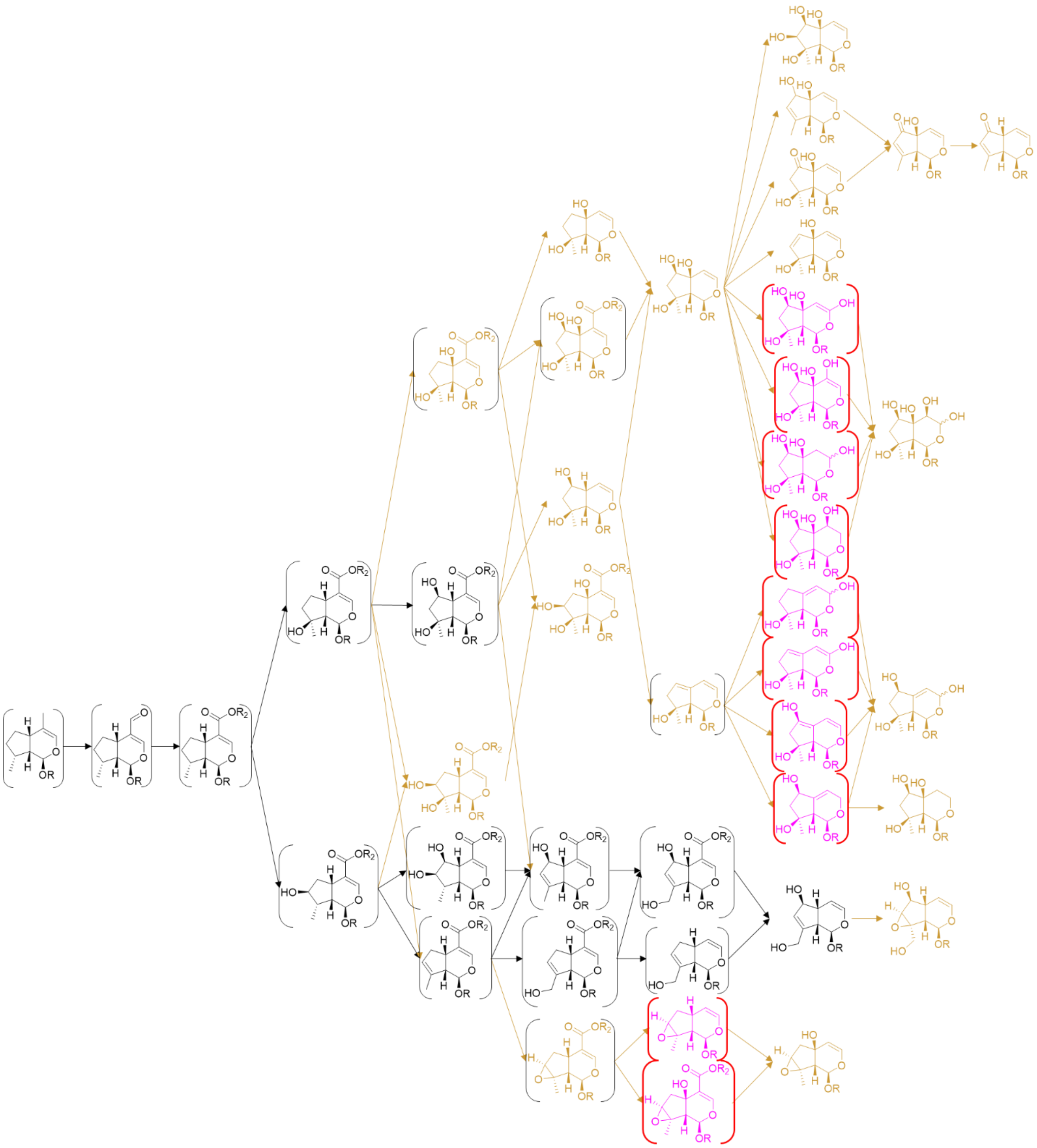

Supplementary Figure S18. Pathway Hypothesis for Ajuga spp. (Ajugoideae). The metabolites and reactions expected to be present in the ancestral pathway are shown in black; metabolites reported in this genus, but not expected to be ancestral, are shown in Tussock Yellow; and completely theoretical metabolites are shown in pink. Metabolites predicted by our model, but that are not reported in the genus, are shown brackets.

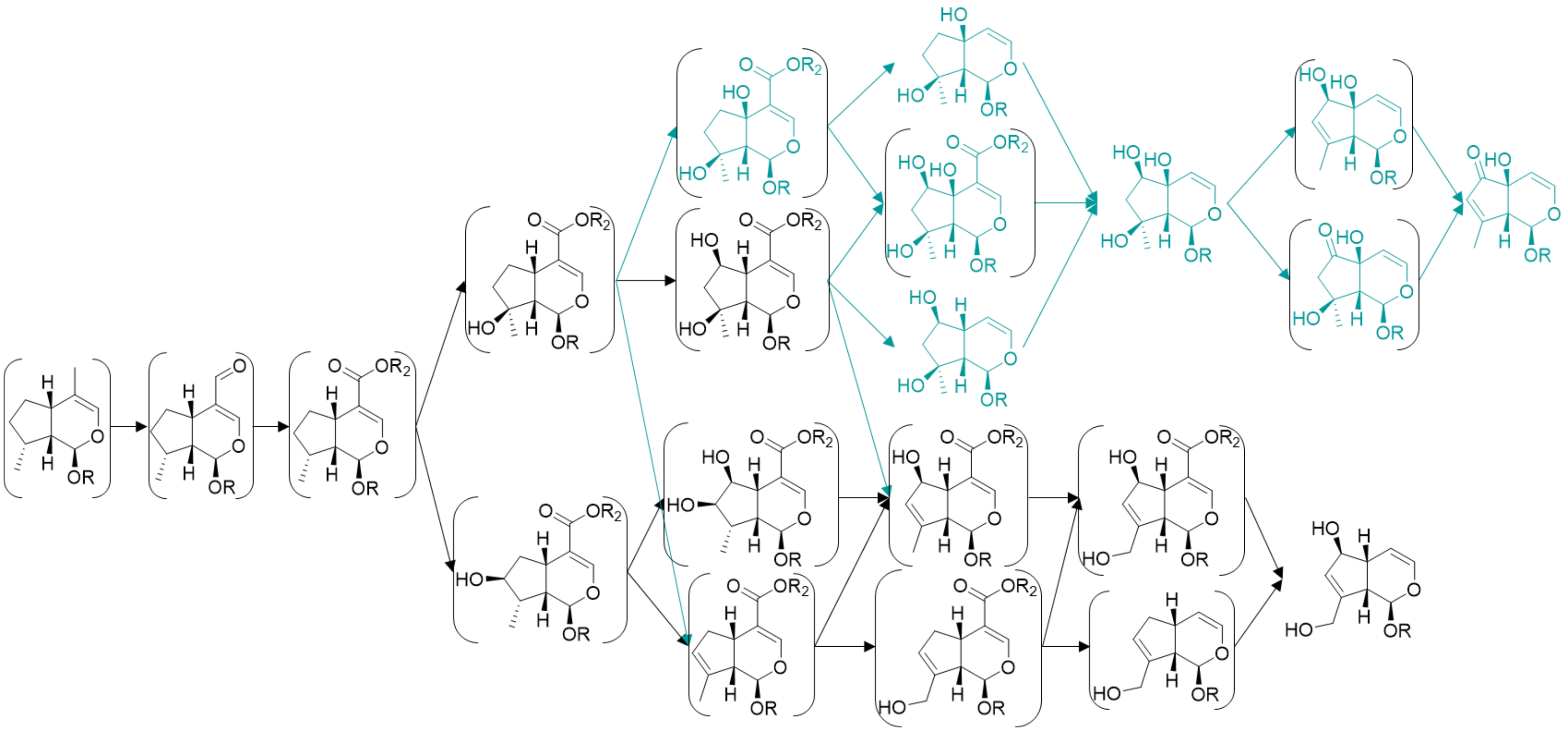

Supplementary Figure S19. Pathway Hypothesis for Betonica spp. (Lamioideae). The metabolites and reactions expected to be present in the ancestral pathway are shown in black; metabolites reported in this genus, but not expected to be ancestral, are shown in Persian Green. Metabolites predicted by our model, but that are not reported in the genus, are shown brackets.

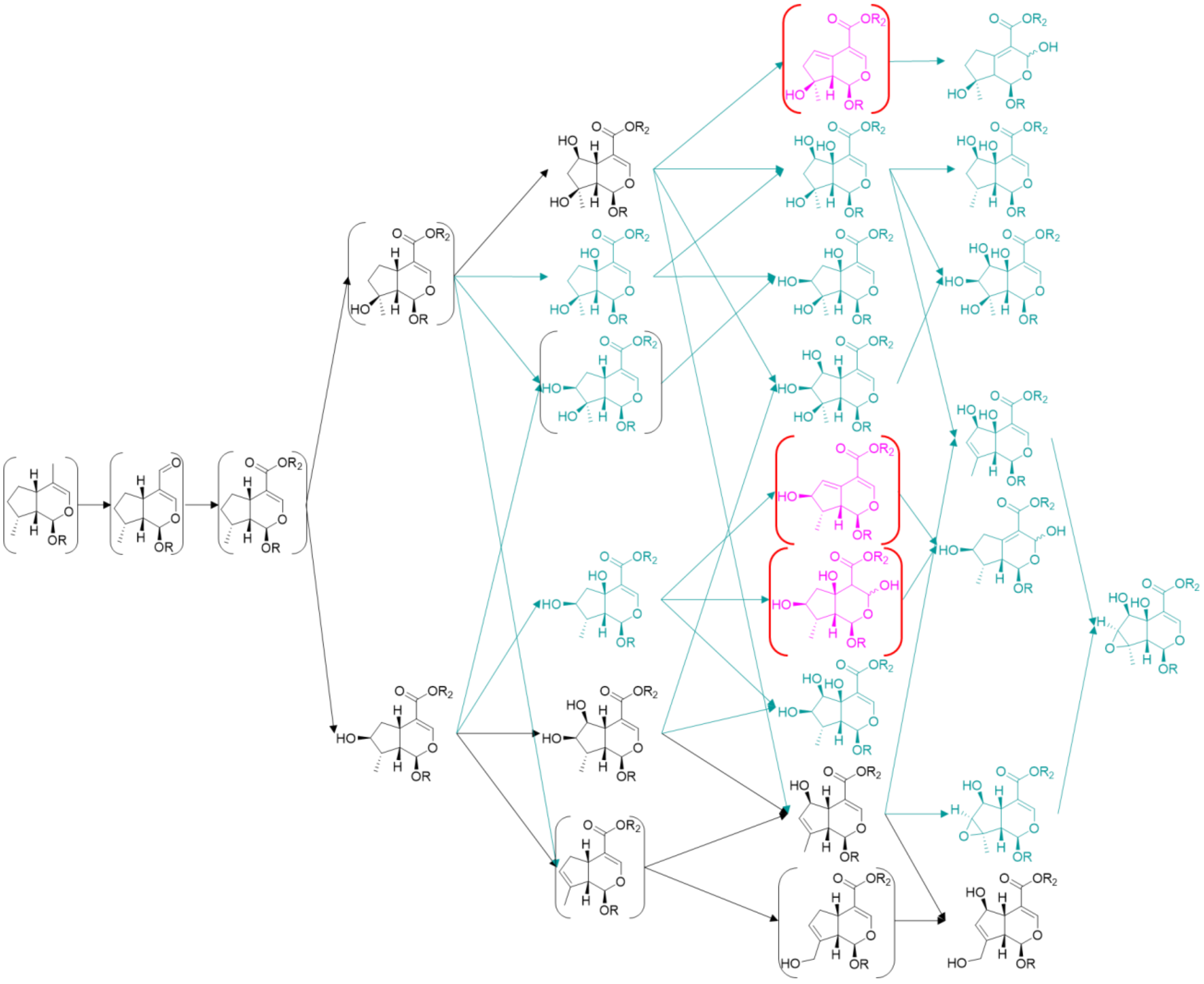

Supplementary Figure S20. Pathway Hypothesis for Phlomis spp. (Lamioideae). The metabolites and reactions expected to be present in the ancestral pathway are shown in black; metabolites reported in this genus, but not expected to be ancestral, are shown in Persian Green; and completely theoretical metabolites are shown in pink. Metabolites predicted by our model, but that are not reported in the genus, are shown brackets.

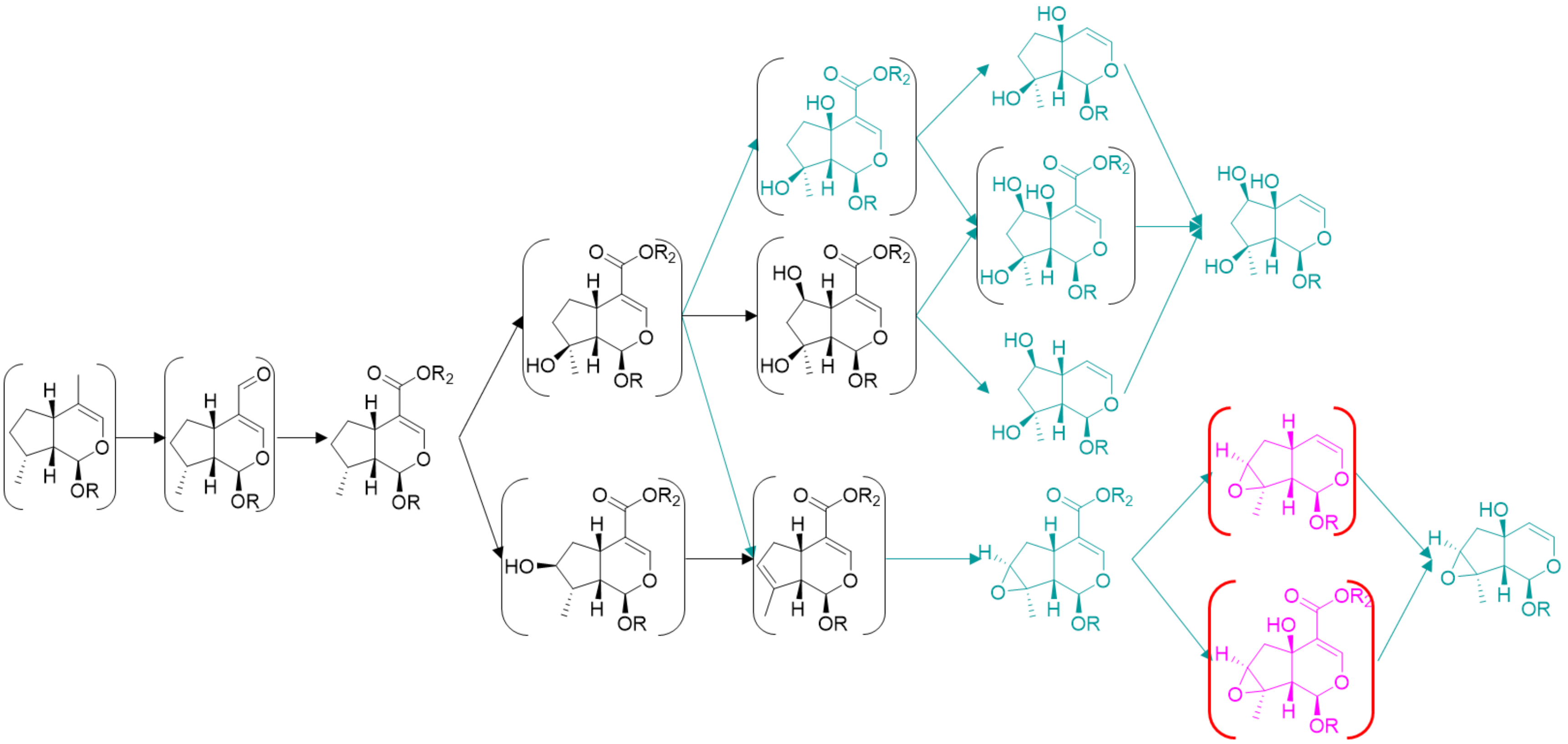

Supplementary Figure S21. Pathway Hypothesis for Leonurus spp. (Lamioideae). The metabolites and reactions expected to be present in the ancestral pathway are shown in black; metabolites reported in this genus, but not expected to be ancestral, are shown in Persian Green; and completely theoretical metabolites are shown in pink. Metabolites predicted by our model, but that are not reported in the genus, are shown brackets.

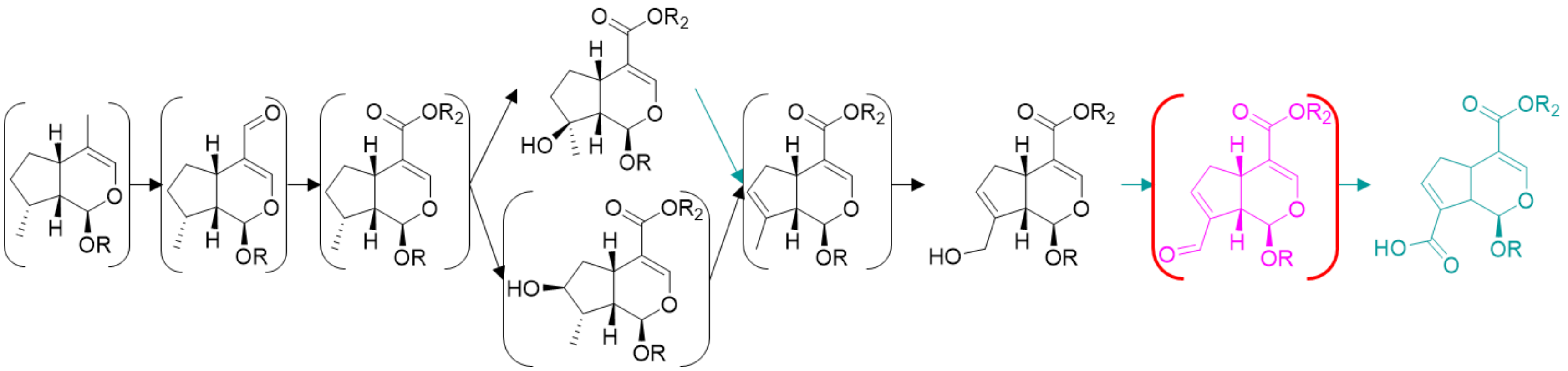

Supplementary Figure S22. Pathway Hypothesis for Leonotis spp. (Lamioideae). The metabolites and reactions expected to be present in the ancestral pathway are shown in black; metabolites reported in this genus, but not expected to be ancestral, are shown in Persian Green; and completely theoretical metabolites are shown in pink. Metabolites predicted by our model, but that are not reported in the genus, are shown brackets.

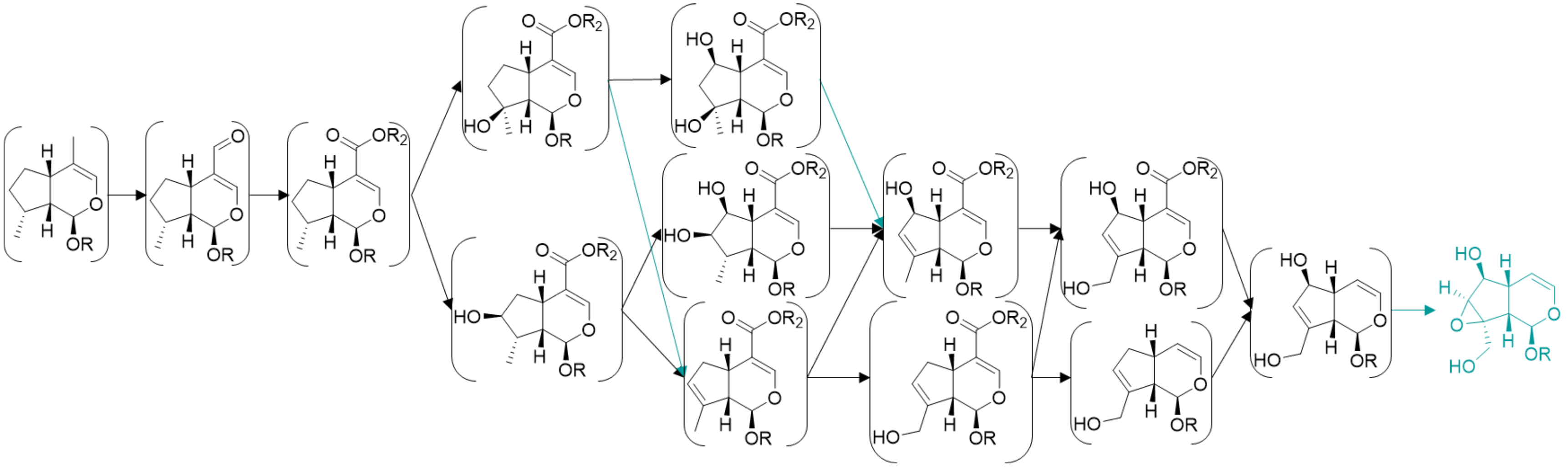

Supplementary Figure S23. Pathway Hypothesis for Ballota spp. (Lamioideae). The metabolites and reactions expected to be present in the ancestral pathway are shown in black; metabolites reported in this genus, but not expected to be ancestral, are shown in Persian Green. Metabolites predicted by our model, but that are not reported in the genus, are shown brackets.

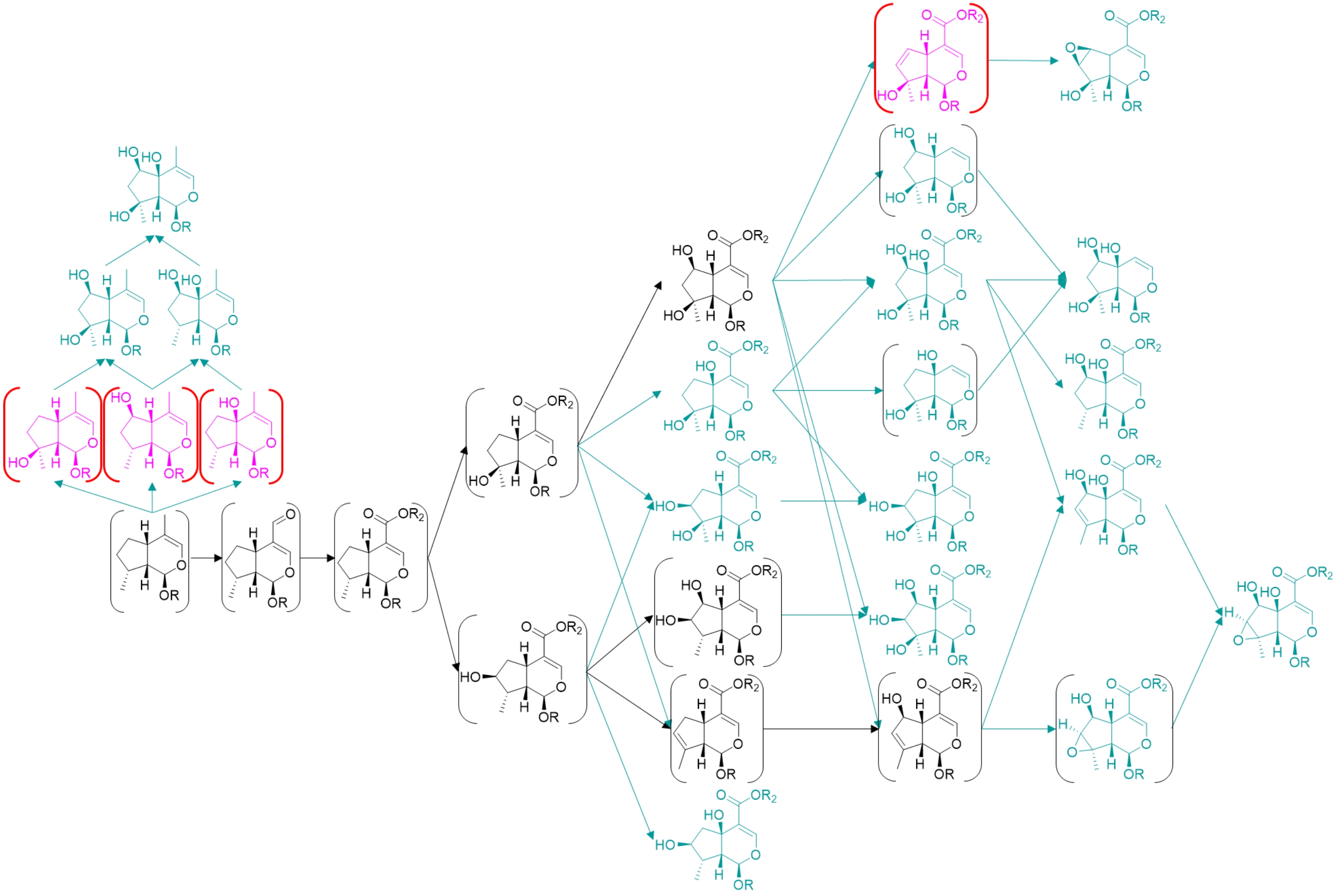

Supplementary Figure S24. Pathway Hypothesis for Lamium spp. (Lamioideae). The metabolites and reactions expected to be present in the ancestral pathway are shown in black; metabolites reported in this genus, but not expected to be ancestral, are shown in Persian Green; and completely theoretical metabolites are shown in pink. Metabolites predicted by our model, but that are not reported in the genus, are shown brackets.

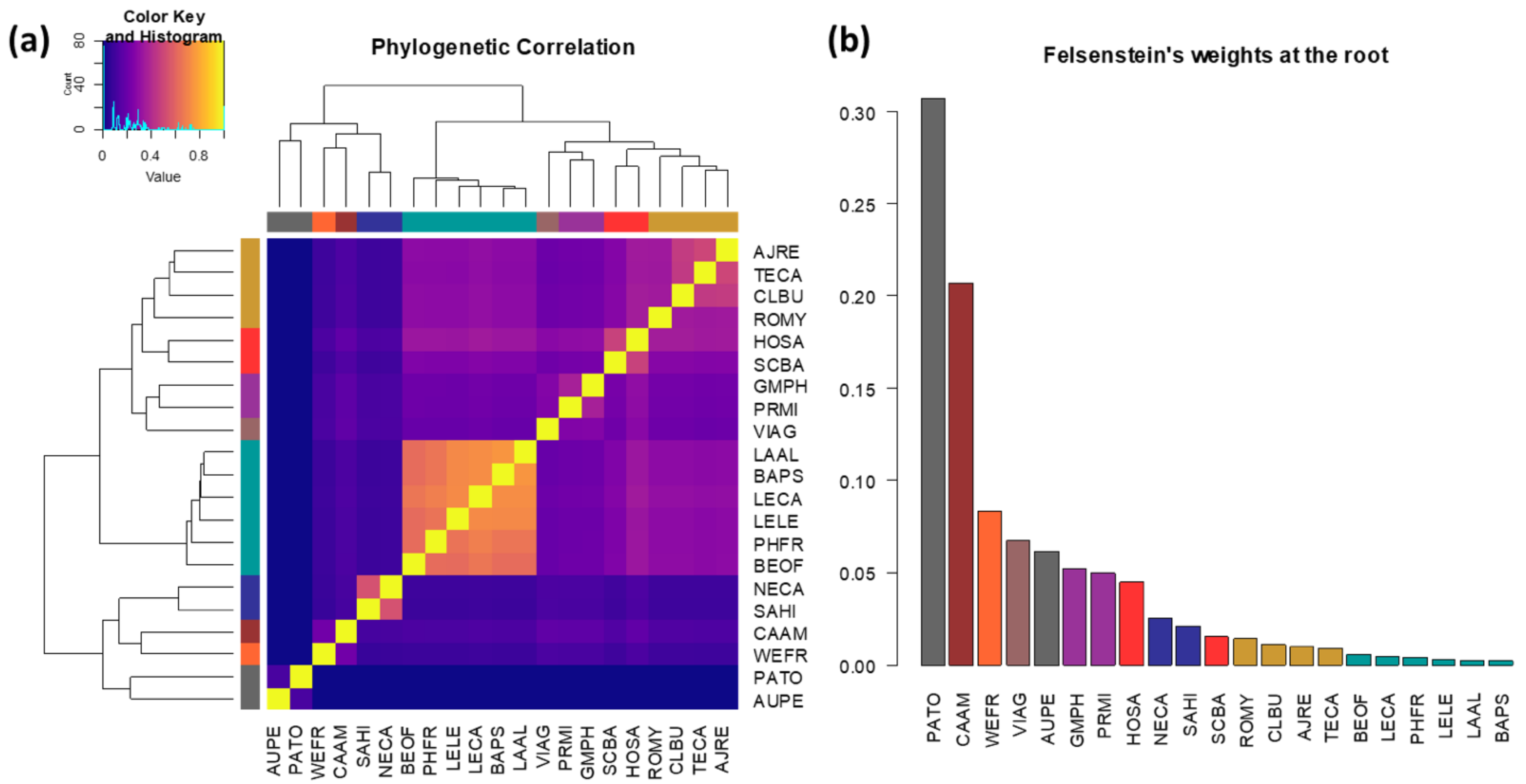

Supplementary Figure S25. Graphical representation of phylogenetic weights. A heatmap is shown in (a), corresponding to the correlation matrix, with the side dendrograms corresponding to the phylogenetic tree. For Felsenstein's weights at the root (b) a barplot is shown, with the labels ordered by weight. Four letter codes correspond to representative species for each genus, and colors to the clades, as in the main text.

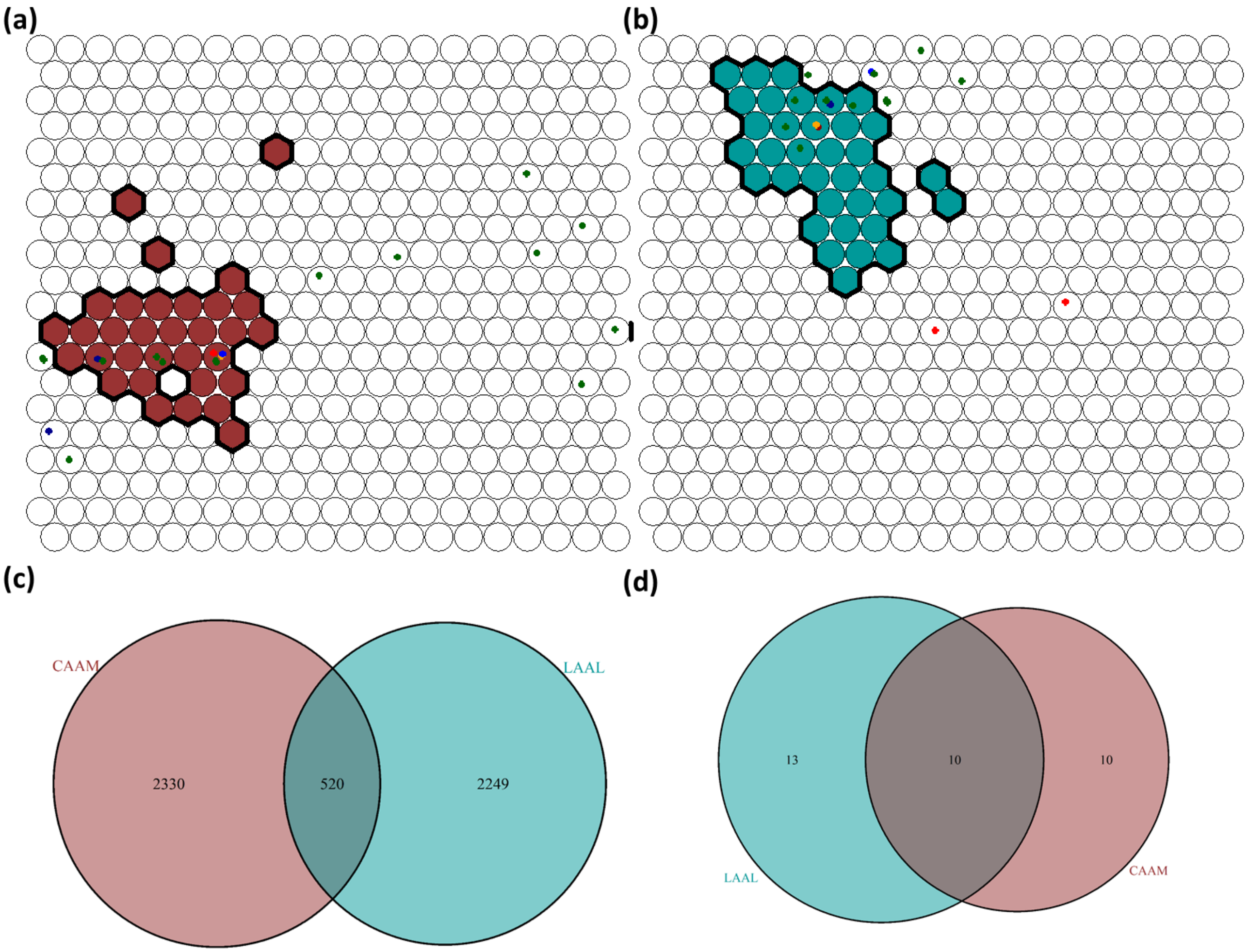

Supplementary Figure S26. Candidate gene selection. Self-Organizing maps are shown, clustering the expression patterns of *Callicarpa americana* (**a**) and *Lamium album* (**b**) tissue panels. Neurons belonging to the clusters enriched in biosynthetic genes ($p<1x10^{-5}$ for *C. americana* and $p<0.02$ for *L. album*) are shown in solid colors. Early pathway iridoid biosynthetic gene candidates (DXS, DXR, MCT, CMK, MDS, HDS, GGPPS) are shown as dark green dots, GES in blue, HGOA in dark blue, ISY in red and IO in Orange. Venn diagrams of orthogroup classifications are shown for all candidates (**c**) and candidates that had a pfam assignation as p450 enzymes; *C. americana* is shown in Crail Red, and *L. album* in Persian Green.

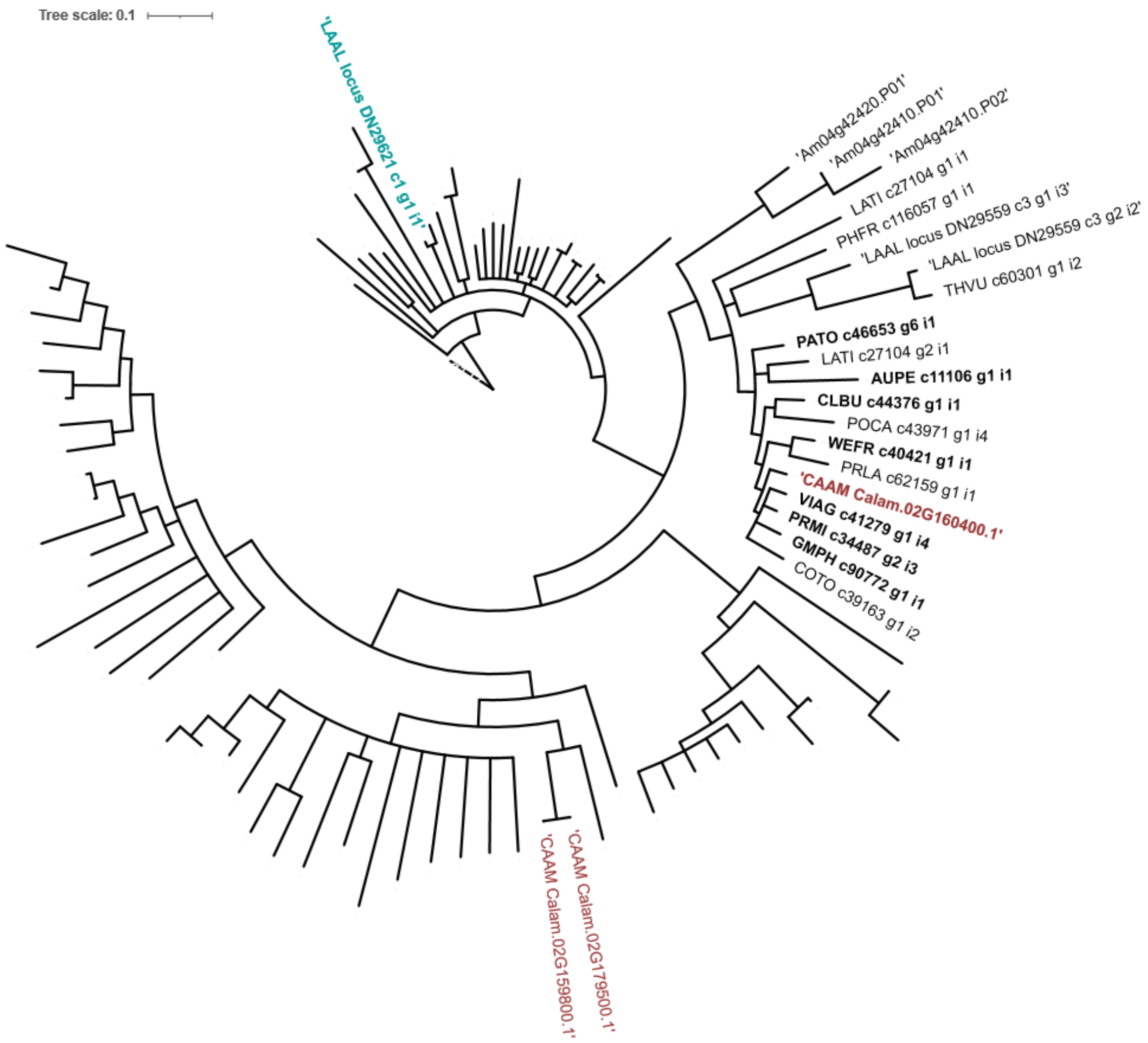

Supplementary Figure S27. Protein alignment tree of Orthogroup OG0001031. Protein alignment tree, using ClustalW for multiple alignment and Jukes-Cantor genetic distance model for tree generation, of all members of orthogroup OG0001031, enriched in CYP72 annotations. In bold, species that are predicted to have bartsioside, and in color, candidates that coexpress with iridoid biosynthetic enzymes, selected by SOM clustering.

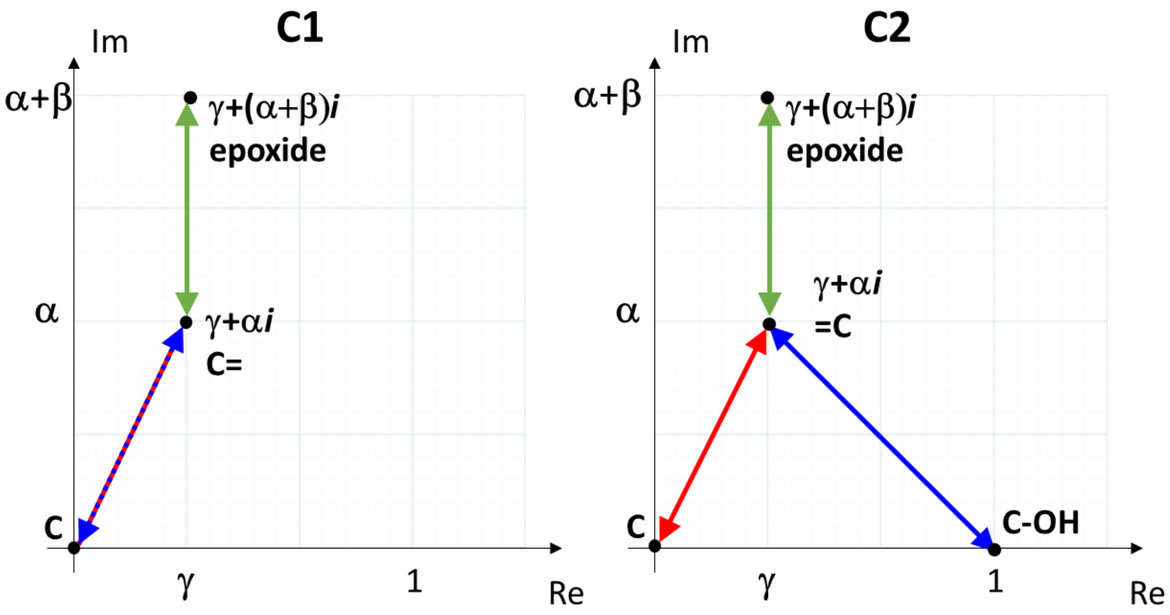

Supplementary Figure S28. Graphical representation of the difference vectors in two carbons. The x-axis represents the real axis, and the y-axis, the imaginary axis. Values of α, β and γ should be selected so the sum of the green vectors in both carbons should give a value of 1, while the sum of both red vectors should not. That is not possible for the sum of the blue vectors, due to the triangle inequality, so an approximation was used.

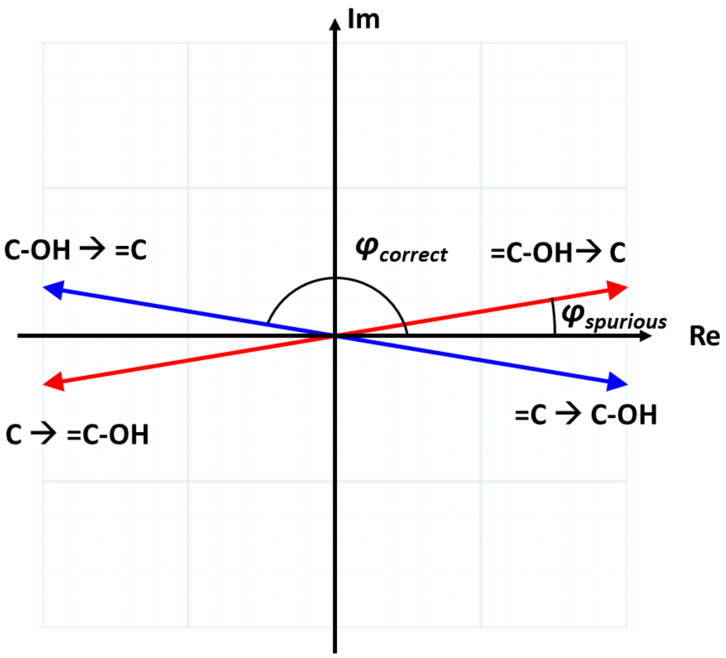

Supplementary Figure S29. Graphical representation of reaction vectors. The x-axis represents the real axis, and the y-axis, the imaginary axis. In blue, two vector representing dehydration (quadrant II) and hydration (quadrant IV) reactions, which are correct. In red, two vectors representing spurious "superoxidation" reactions that have the same magnitude, but different direction (quadrants I and III).

(a)

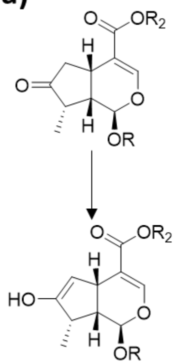

p	C3	C4	C5	C6	C7	C8	C10	C11
	i/4	*i*/4	0	0	2	0	0	2

q	C3	C4	C5	C6	C7	C8	C10	C11
	i/4	*i*/4	0	*i*/4	1+*i*/4	0	0	2

(b)

p-q	C3	C4	C5	C6	C7	C8	C10	C11
	0	0	0	-*i*/4	1-*i*/4	0	0	0

tan(φ)	C3	C4	C5	C6	C7	C8	C10	C11
	NA	NA	NA	-∞	-1/4	NA	NA	NA

|p-q|	C3	C4	C5	C6	C7	C8	C10	C11
	0	0	0	1/4	1.031	0	0	0

$$\sum_{j=1}^{n} \|p_j - q_j\| = 1.280776$$

(c)

Difference has 0 or 2 complex values

$tan(\varphi_i) \neq ¼$

$$\sum_{j=1}^{n} \|p_j - q_j\| \approx 1$$

(d)

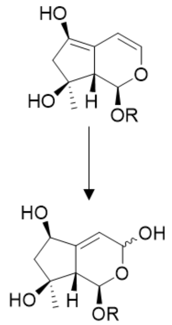

p	C3	C4	C5	C6	C7	C8	C10	C11
	i/4	*i*/4	*i*/4	1+*i*/4	0	1	0	3

q	C3	C4	C5	C6	C7	C8	C10	C11
	1	*i*/4	*i*/4	1	0	1	0	3

(e)

p-q	C3	C4	C5	C6	C7	C8	C10	C11
	-1+*i*/4	0	0	*i*/4	0	0	0	0

tan(φ)	C3	C4	C5	C6	C7	C8	C10	C11
	-1/4	NA	NA	∞	NA	NA	NA	NA

|p-q|	C3	C4	C5	C6	C7	C8	C10	C11
	1.031	0	0	1/4	0	0	0	0

$$\sum_{j=1}^{n} \|p_j - q_j\| = 1.280776$$

(f)

Difference has 0 or 2 complex values

$tan(\varphi_i) \neq ¼$

$$\sum_{j=1}^{n} \|p_j - q_j\| \approx 1$$

Supplementary Figure S30. Distance calculations for two sample reactions. An example is shown of of a double bond emerging from keto-enol tautomerism (a) and a 1,4 reduction (d). Step by step calculations are shown in (b) and (d) for each molecule, including the difference between both molecule vectors (top), the tangent of the phase (middle) and the modulus of each difference, and the generalized Manhattan distance (bottom). Finally, (c) and (f) show the checklist confirming that each pair of molecules fulfills the requirements set to be connected in our model.

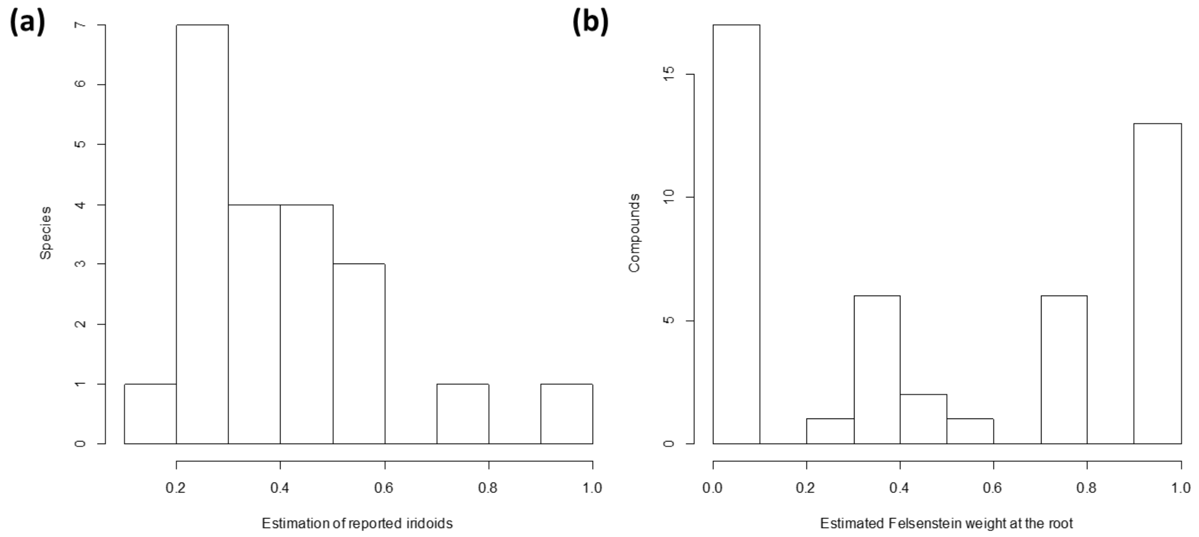

Supplementary Figure S31. Parameter estimation. Estimation of reported iridoids (a) after a naïve (n=0) reconstruction of the pathway, via **Algorithm S1**. (b) Shows a histogram of Felsenstein's weights at the root values, calculated for the *in natura* iridoid reports.

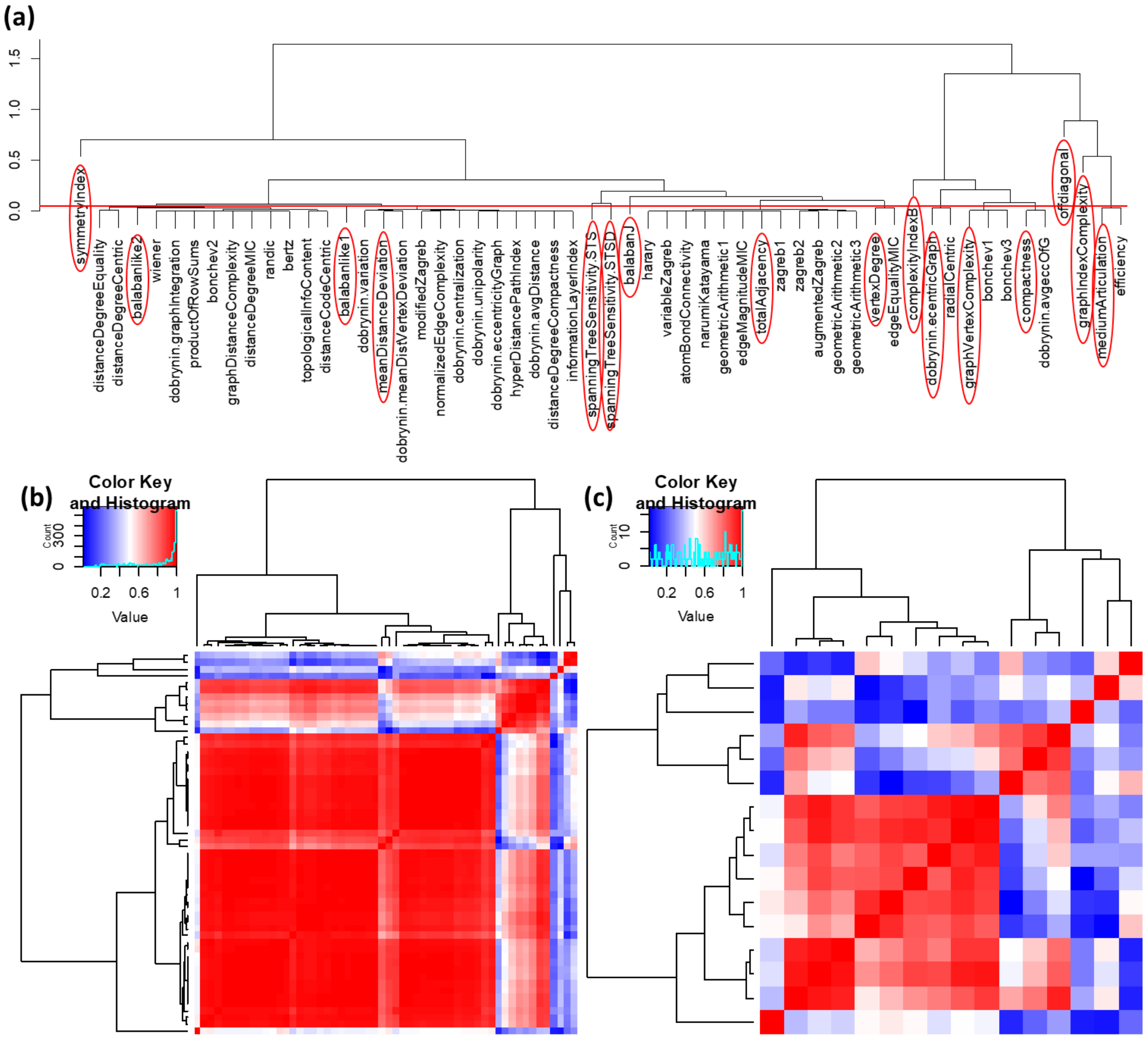

Supplementary Figure S32. Selection of Network Descriptors. Hierarchical clustering of correlation coefficients of network descriptors (a) showing, in red circles, the representative metric for each one of the 16 clusters selected. The original 55 descriptors (b) were reduced to 16 selected descriptors (c) greatly reducing redundancy. Representative metrics were selected by complementarity and interpretability.

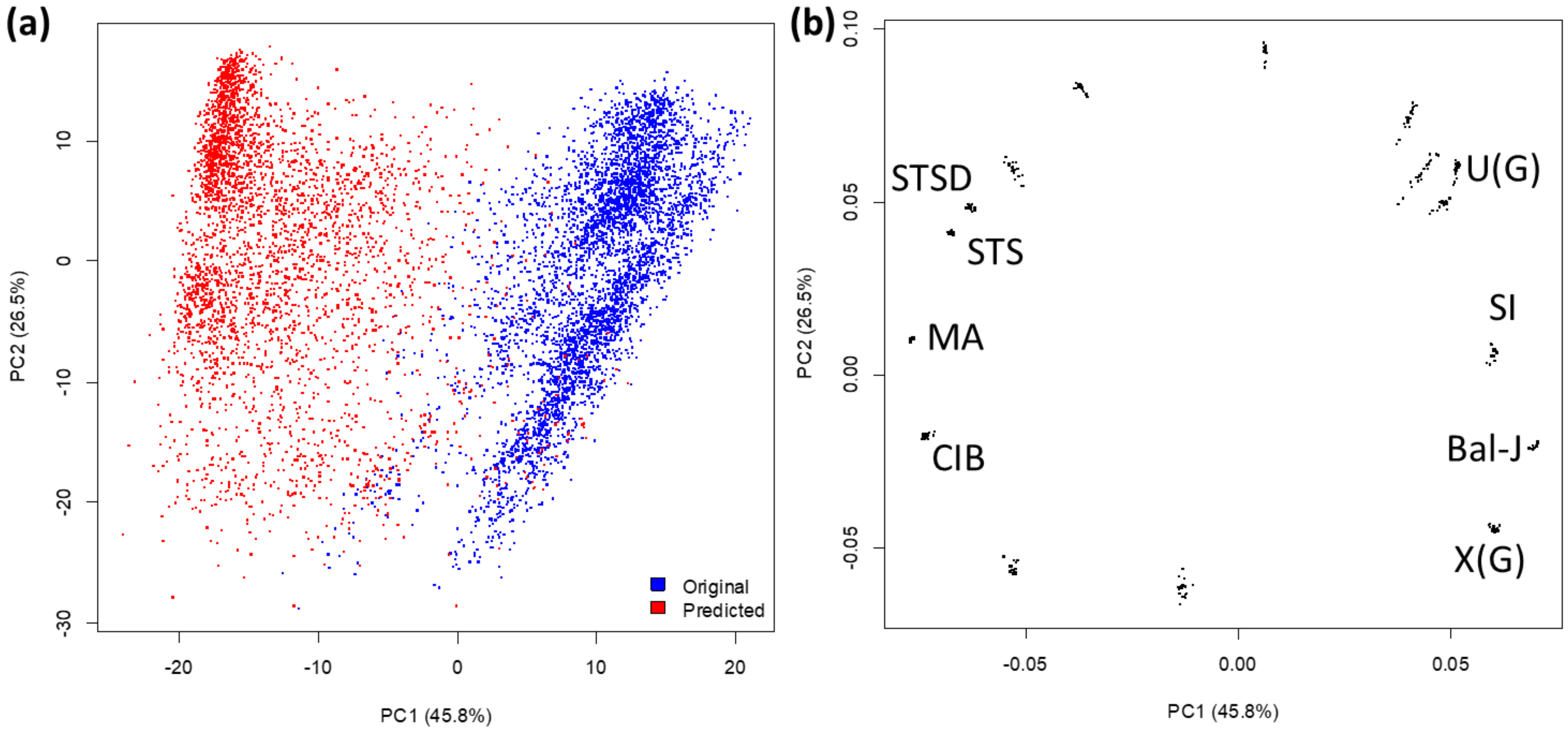

Supplementary Figure S33. Principal Component Analysis of Selected Network Descriptors. The loadings (a) and scores plots (b) are shown for the original *in silico* networks (blue) and their naïve reconstruction (red) predicted with 100% sampling. It can be seen that the original *in silico* pathways have a higher symmetry index (SI), Balaban J (Bal-J) and Balaban-like Information Indices U(G) and X(G). On the other hand, naïve predictions have more interconnections, resulting in higher medium articulation (MA), complexity (CIB), and spanning tree sensitivity (STS, and STSD).

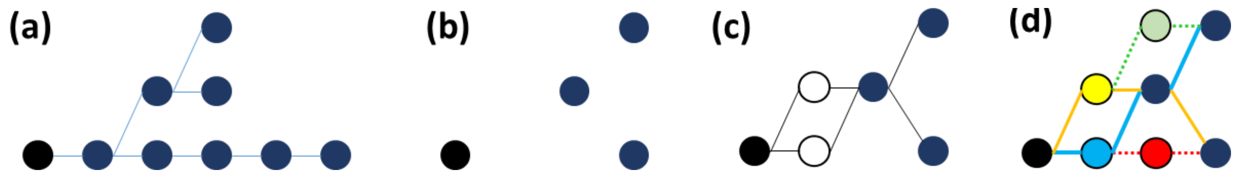

Supplementary Figure S34. Evaluating the model. A schematic is shown for the general reconstruction process, where (a) depicts a biosynthetic pathway for a given species, (b) is the sampled chemical diversity and (c) would be the pathway hypothesis. An example of the evaluation parameters is shown in (d), where false positives are shown in red, true positives in cyan, false positives in yellow, and true negatives in green.

Supplementary Table S1. Network Descriptors by category, along with their abbreviation.

**Category**	**Network Descriptors**
1xxx – Based on distances in a graph	Mean Distance Deviation ("meanDistanceDeviation")<br>Balaban J Index ("balabanJ")<br>Graph Compactness (“compactness")<br>The eccentric of the graph ("dobrynin.ecentricGraph")
2xxx – Based on graph invariants	Index of Total Adjacency ("totalAdjacency")<br>The Complexity Index B ("complexityIndexB")
3xxx – Classical entropy-based descriptors	Balaban-like Information Indices U(G) and X(G) ("balabanlike1","balabanlike2")<br>Symmetry Index ("symmetryIndex")<br>Vertex Degree Equality-based Information Index ("vertexDegree")
4xxx – Graph complexity measures	Graph Index Complexity ("graphIndexComplexity")<br>Offdiagonal complexity ("offdiagonal")<br>Spanning tree sensitivity ("spanningTreeSensitivity.STS", and "spanningTreeSensitivity.STSD")<br>Medium Articulation ("mediumArticulation")<br>Graph Vertex Complexity Index ("graphVertexComplexity")

Algorithm S1 Pseudocode Algorithm of the naïve model

**Input:**	Matrix ***X*** with 8 columns, each representing a Carbon, and *64* rows, each being a molecule
**Input:**	List ***reported_molecules*** relating which rows of **X** have been reported in each species
**Input:**	Integer ***n***, stating the number of reactions to extend the reported molecules
**Note:**	all functions on rows are applied vectorially (as in the R programming language)

```
begin
     if n>0 then do
          for i = 1 to n do
               F = sum_reactions(X)
               R = subtract_reactions(X)

               New = append(X,F,R)

               remove_duplicates(New)
               remove_negatives(New)
               remove_invalid_C_valence(New)

               X = New
          end for
     end if

     adjacency = apply Algorithm S4 to X
     graph = make graph from adjacency

     declare list of graphs results
     for each species do
          results[species] = all_shortest_paths(in = graph,
          from = nepetalactol, to = reported_molecules[species])
     end for

     Output:   results
end
```

Algorithm S2 Pseudocode Algorithm of the phylogenetic pruning

Input: List of graphs ***G*** with as many graphs as species; the output of **Algorithm S1**

Input: Boolean matrix ***reported_molecules*** with as many columns as species, and as many rows as unique nodes in ***G***. A cell is **TRUE** if the molecule in that row is reported in the species in that column.

Input: Boolean matrix ***predicted_molecules*** with as many columns as species, and as many rows as unique nodes in ***G***. A cell is **TRUE** if the molecule in that row is predicted in the species in that column.

Input: Phylogenetic tree ***tree***, with the phylogenetic relationships between species

Input: Boolean ***predicted*** which states whether to use predicted molecules in addition to the reported molecules

Input: Character ***method*** with values "C" or "F" states which method to use when calculating weights (Correlation or Felsenstein)

Input: Numeric ***tol***, a value between 0 and 1, stating the percentage of the maximum score to be allowed

Note: all functions on rows are applied vectorially (as in the R programming language)

Note: The operator %*% denotes Matrix Multiplication (as in the R programming language)

```
begin

     if (predicted) then do
          M = reported_molecules or predicted_molecules;
     else then do
          M = reported_molecules;
     end if

     V = apply Eq.S2 to tree
     C = apply Eq.S1 to V
     F = apply Eq.S3 to V

     if (method == "F") then do
          weights = M %*% F
     end if

     for each species do
          if (method == "C") then do
               weights = M %*% C[,species]
          end if
```

```
        for each reported_molecules[,species]
             G.path = all_shortest_paths(in = G[species],
                    from = nepetalactol, to = current_molecule)
             for each G.path
                  G.path.score = sum(weights[nodes(path)])
             end for

             keep = G.path.score > (max(G.path.score)*(1-tol))
             G.new.paths = G.path[keep]

        end for

        G[species] = graph_from_paths(G.new.paths)
    end for

    Output:   G
end
```

Algorithm S3 Pseudocode Algorithm of the Pathway Evolution Model

**Input:**	Phylogenetic tree ***tree***, of 21 selected species, as shown in **Fig. 1a**
**Input:**	Graph ***Space*** of 6.24x10^4 iridoid scaffolds (nodes) connected by 8.22x10^5 reactions (as edges). This graph is in **Supplementary File 3**, saved as an rds file (R).
**Input:**	Integer ***root_rxn*** stating the number of reactions present in the common ancestor
**Input:**	Integer ***max_enzymes*** stating the number of enzymes per species to which limit the exploration
**Input:**	Integer ***new_activities*** stating the number of rounds of exploration per unit of distance in **tree**
**Note:**	all functions on rows are applied vectorially (as in the R programming language)

```
begin function random_pathway
     Input:    Graph Space
     Input:    Integer max_enzymes
     Input:    Integer limit
     Input:    Character type stating whether the limit is on
          number of edges ("E") or exploration rounds ("X")
     Input:    Graph graph containing the pathway to be extended

     counter = 0
     while counter < limit
          new_edge = sample_random_edge(Space)
          if new_edge.tail is in graph.nodes then do
               origin = add_edge(new_edge, to = graph)

               if length(graph.nodes)>max_enzymes then do
                    if has_unconnected_edges(graph) then do
                         remove_unconnected_edge(graph)
                    else
                         remove_random_edge(graph)
                    end if
               end if

               if limit == "E" then do
                    counter = counter + 1
               end if

          end if

          if limit == "X" then do
               counter = counter + 1
          end if
```

```
    end while
    return(graph)
end function

begin

    root = random_pathway(graph = nepetalactol, limit=root_rxn,
          type = "E")

    define list of graphs results of length nodes(tree)+1

    results[1] = root
    for each tree.node
        results[current_node]= random_pathway(
              graph = results[parent_node],
              limit = new_activities * distance(tree,
                   from = current_node, to = parent_node),
              type = "X")
    end for

    Output:   results
end
```

Algorithm S4 Simplified algorithm generating an adjacency matrix from codified molecules

```
Input:    Matrix X with N columns, each representing a Carbon, and
          M rows, each being a codified molecule
Note:     all functions on rows are applied vectorially (as in
          the R programming language)
```

```
begin
     declare numeric matrix D of M columns and M rows
     declare boolean matrix V of M columns and M rows
     for j = 1 to (M-1) do
          for k = (j+1) to M do
               D[j,k]= D[k,j]=round( sum( M,d(X[j,]-X[k,]) ) )
               if any ( tan(Arg(X[j,]-X[k,]))==0.25 ) then do
                    V[j,k]= V[k,j]= FALSE
               else if sum(Im(X[j,]-X[k,])!=0)>2
                    V[j,k]= V[k,j]= FALSE
               else if (sum((Im(X[j,])>0)&!(Im(X[k,])>0))!=0) or
                    (sum((Im(X[k,])>0)&!(Im(X[j,])>0))!=0) or
                    (sum((Im(X[j,])>0)&!(Im(X[k,])>0))!=2) or
                    (sum((Im(X[k,])>0)&!(Im(X[j,])>0))!=2)then do
                    V[j,k]= V[k,j]= FALSE
               else
                    V[j,k]= V[k,j]= TRUE
               end if
          end for
     end for
     adjacency = (D==1)& V
     Output:   adjacency
end
```

References

Felsenstein J. 1985a. Confidence Limits on Phylogenies: An Approach Using the Bootstrap. Evolution 39:783-791.

Felsenstein J. 1985b. Phylogenies and the Comparative Method. The American Naturalist 125:1-15.

Garland T, Jr., Ives AR. 2000. Using the Past to Predict the Present: Confidence Intervals for Regression Equations in Phylogenetic Comparative Methods. Am Nat 155:346-364.

Martins EP, Hansen TF. 1997. Phylogenies and the Comparative Method: A General Approach to Incorporating Phylogenetic Information into the Analysis of Interspecific Data. The American Naturalist 149:646-667.

Mueller LA, Kugler KG, Dander A, Graber A, Dehmer M. 2011. QuACN: an R package for analyzing complex biological networks quantitatively. Bioinformatics 27:140-141.
